# Insight into the Course of the Ferrier Rearrangement Used to Obtain Untypical Diosgenyl Saponins

**DOI:** 10.1021/acs.joc.4c01756

**Published:** 2024-10-05

**Authors:** Grzegorz Detlaff, Magdalena Zdrowowicz, Małgorzata Paduszyńska, Magdalena Datta, Daria Grzywacz, Wojciech Kamysz, Janusz Rak, Andrzej Nowacki, Henryk Myszka, Beata Liberek

**Affiliations:** † Faculty of Chemistry, University of Gdańsk, Wita Stwosza 63, 80-308 Gdańsk, Poland; ‡ Faculty of Pharmacy, Medical University of Gdańsk, Hallera 107, 80-416 Gdańsk, Poland

## Abstract

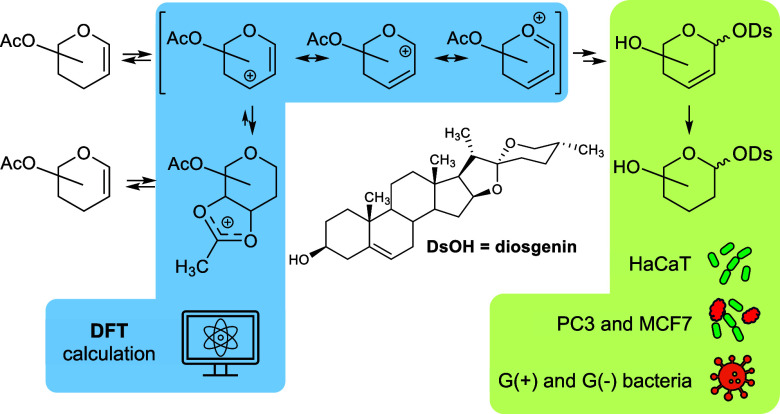

The Ferrier rearrangement was utilized to obtain 2,3-unsaturated diosgenyl glycosides. This reaction proceeded with high stereoselectivity, yielding mostly saponins with an α configuration (hexoses) or predominantly with a β configuration (pentoses). The diversity of the glycals used and the glycosides obtained enabled a deep discussion of the Ferrier rearrangement mechanism. The mechanism was supported by DFT calculations concerning the intermediate ions. It was concluded that the vinylogous anomeric effect may influence the reactivity of the glycals. Two possible Ferrier rearrangement intermediates, dioxolenium and allyloxycarbenium ions, were hypothesized to exist in thermodynamic equilibrium that shifted toward the former. The allyloxycarbenium ion participates in the final rearrangement step and determines the reaction regioselectivity. Furthermore, the conformational stability of the 2,3-unsaturated pyranose ring determines the stereoselectivity of the reaction. Factors influencing this stability, as well as the NMR data enabling recognition of the ^0^H_5_ and ^5^H_0_ conformations, were identified. Chemoselective hydrogenation of 2,3-unsaturated diosgenyl glycosides provided a series of 2,3-dideoxy analogues. The anticancer, hemolytic, and antibacterial activities of the synthesized saponins are presented alongside a discussion of the structure–activity relationships.

## 1 Introduction

Diosgenin is a phytosteroidal sapogenin found in nature in the form of glycosides.^1^ Due to its impressive pharmacological profile,^2−7^ diosgenin is commonly isolated from plants.^8−13^ Methods of its synthesis have also been investigated.^14^ To enhance its pharmacological properties, diosgenin is often chemically modified.^15−20^

Diosgenyl glycosides constitute a group of intensively explored diosgenin derivatives. Much attention is devoted to dioscin, a natural diosgenyl trisaccharide,^21−26^ but other glycosides of diosgenin have also been investigated.^27−31^ Among them, diosgenyl glucosaminosides are of great interest because it was found that diosgenyl 2-amino-2-deoxy-β-d-glucopyranoside hydrochloride induces apoptosis and necrosis in some leukemic B-cells.^32^ The amino group in glucosamine creates the possibility of chemical modifications, which is why diosgenin is often glycosylated with this sugar.^33^

This work presents the application of the Ferrier rearrangement to the synthesis of 2,3-unsaturated diosgenyl glycosides. The Ferrier rearrangement, introduced to sugar chemistry in 1962,^34^ is a useful route leading to 2-enopyranoses that hold a high synthetic potential, as described in subsequent reviews.^35−38^ The structural diversity of the obtained 2,3-unsaturated glycosides and their substrates allowed for a thorough discussion of Ferrier rearrangement mechanism and provided novel insight into its course and the factors influencing it. The obtained saponins have an atypical pyranose ring structure and, in most cases, α configuration of the anomeric carbon atom, which have not been reported in the literature so far. Chemoselective hydrogenation of 2,3-unsaturated diosgenyl glycosides provided 2,3-dideoxy diosgenyl glycosides. The cytotoxic and antimicrobial activities of all synthesized saponins are presented along with a discussion on the structure–activity relationships.

## 2 Results and Discussion

### 2.1 Synthesis

**Scheme 1:**
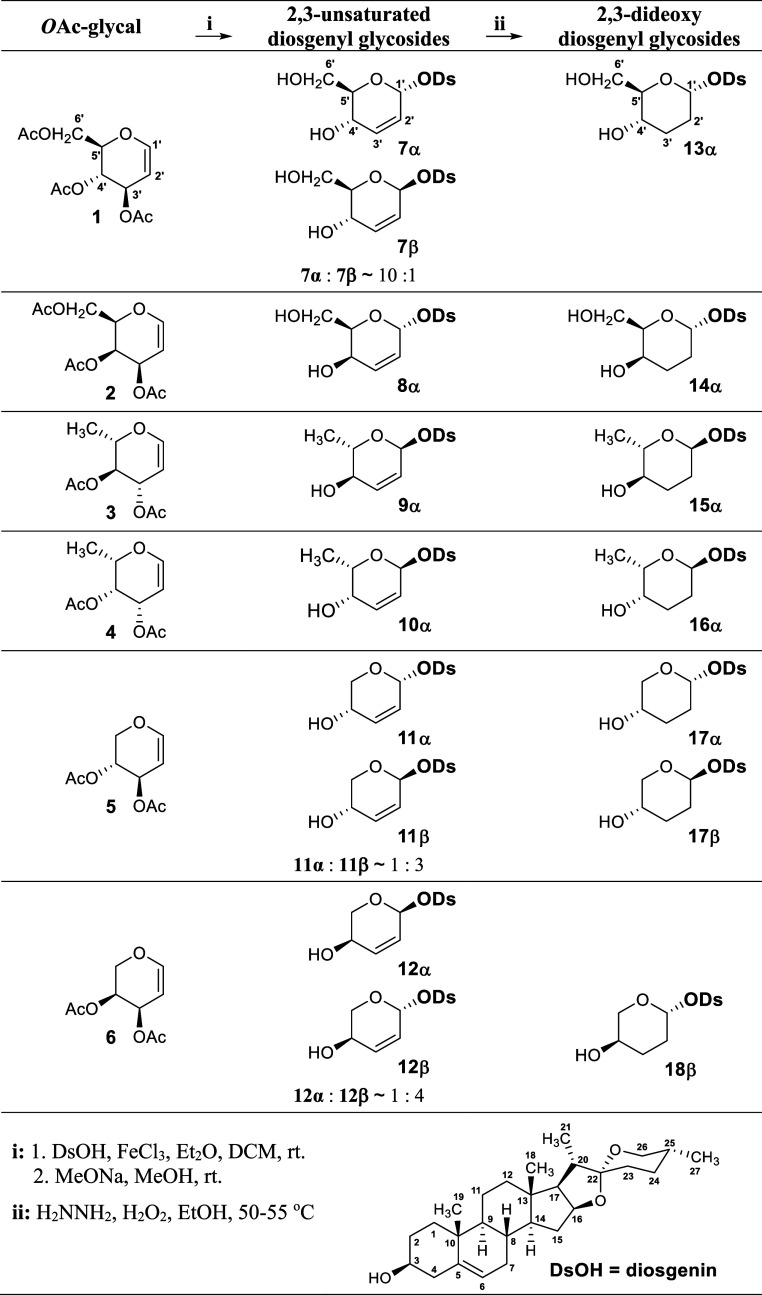
Synthesis of 2,3-Unsaturated (**7**–**12**) and 2,3-Dideoxy (**13**–**18**) Diosgenyl Glycosides along with the Atom Numbering System Used in This Paper

Six *O*-acetylated glycals, namely, d-glucal (**1**), d-galactal (**2**), l-rhamnal (**3**), l-fucal (**4**), d-xylal (**5**), and l-arabinal (**6**), were prepared by adopting the procedures described in the literature.^39^ These were then subjected to Ferrier rearrangement, where diosgenin acted as a nucleophile and subsequently de-*O*-acetylated (Scheme 1). Two catalysts, boron trifluoride etherate (BF_3_ × OEt_2_) and iron(III) chloride (FeCl_3_), were tested for coupling glycals **1**–**6** with diosgenin. FeCl_3_ was found to be more efficient in these reactions. Among the tested solvents, the best yields were obtained in a 2:1 mixture of diethyl ether (Et_2_O) and dichloromethane (DCM).

The reactions of glycals with diosgenin, carried out in the presence of FeCl_3_, took 5 min for acetylated d-glucal (**1**), l-rhamnal (**3**), d-xylal (**5**), and l-arabinal (**6**). Acetylated d-galactal (**2**) and l-fucal (**4**) required 15 min to complete the reaction. All these reactions were highly stereoselective, yielding 2,3-unsaturated glycosides with the α configuration solely in the case of **8**–**10**, the highly predominant α configuration in the case of **7** (α/β ∼ 10:1), and the clearly predominant β configuration in the case of **11** (α/β ∼ 1:3) and **12** (α/β ∼ 1:4).

To obtain 2,3-dideoxy diosgenyl glycosides (**13**–**18**), hydrogenation of **7** using palladium on activated charcoal under a hydrogen atmosphere was tested first. Unfortunately, this procedure led to several compounds, including diosgenin hydrogenation products and the products of hydrogenolysis of the glycosidic bond. The latter reaction is justified because the anomeric carbon in a 2,3-unsaturated glycoside is an allylic carbon. It is known that allylic alcohols,^40^ ethers, and esters^41^ tend to undergo hydrogenolysis. A similar unwanted reaction was observed for 2,3-unsaturated sugar 1-*O*-pivalate when subjected to transition-metal-catalyzed hydrogenation of a double bond.^42^

Next, the azo compound diimide (HN=NH) was selected for the transformation of **7**–**12** glycosides into **13**–**18**. Diimide is a reactive species used for hydrogenation of multiple bonds and is known to react more efficiently with disubstituted rather than with trisubstituted olefins.^43^ Notably, diimide did not cause hydrogenolysis of the C1–OPiv bond in 2,3-unsaturated sugar.^42^ Hydrazine hydrate, oxidized by hydrogen peroxide,^44^ was used for the in situ generation of diimide. We found this reagent to be efficient and selective, causing hydrogenation of the double bond in the sugar part only without disturbing the glycosidic bond or the double bond in diosgenin.

### 2.2 Course of the Ferrier Rearrangement

#### 2.2.1 General Considerations

**Scheme 2:**
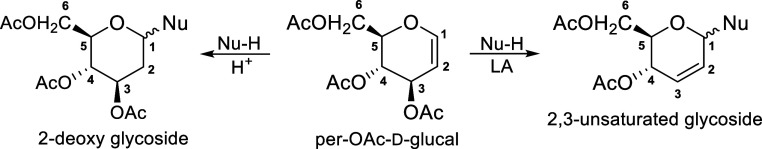
Course of the Reaction of the *O*-Acetylated d-Glucal with a Nucleophile (Nu-H) Dependent on the Nature of the Acid Used (LA—Lewis Acid)

Acetylated glycals treated with nucleophiles in the presence of protic acid tend to undergo acid-catalyzed electrophilic addition, where protonation of the double bond is followed by the attack of the nucleophile at the C1 carbon atom, leading to the formation of 2-deoxyglycosides (Scheme 2).^35^ The same glycals treated with a nucleophile in the presence of a Lewis acid undergo a reaction, leading to 2,3-unsaturated glycosides. This reaction is named the Ferrier rearrangement after its researcher.^34^ While the Ferrier rearrangement can be carried out with the use of protic acids, this procedure is often limited in terms of the chemical yields due to a competitive addition reaction. Similarly, synthesizing 2-deoxyglycosides from glycals in the presence of a protic acid can yield the formation of 2,3-unsaturated glycosides as byproducts. Unlike a protic acid, a Lewis acid cannot initiate electrophilic addition; instead, it cleaves the 3-OAc group, leading to the Ferrier rearrangement.

**Scheme 3:**
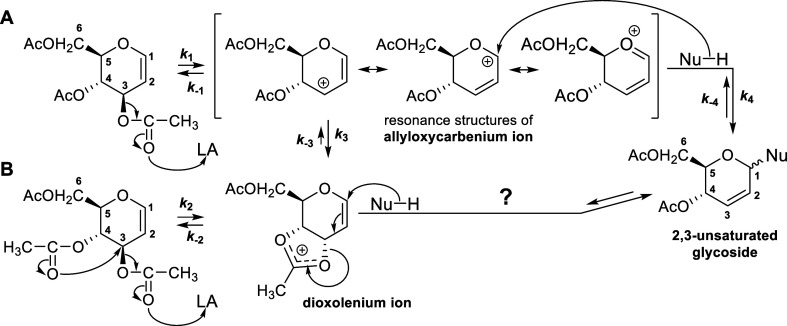
Considered Pathways of the Ferrier Rearrangement by the Example of 3,4,6-Tri-*O*-acetyl-d-glucal: without (A) and with (B) Anchimeric Assistance (LA—Lewis Acid)

Two pathways of the Ferrier rearrangement are considered, both involving a two-step process. The first pathway (Scheme 3A) should be formally classified as a nucleophilic substitution reaction of the S_N_1′ type. It involves an allylic carbon atom and is characterized by the nucleophile attacking a position different from where the nucleofuge leaves.^45^ In the first step of this pathway of the Ferrier rearrangement, a Lewis acid cleaves the 3-OAc group in the acetylated glycal, leading to the formation of a resonance-stabilized allyloxycarbenium ion (Scheme 3A). Cleavage of any other OAc group in the acetylated glycal would not result in the formation of such a stable cation^46^ and, therefore, does not occur. In the second step of the first pathway, nucleophilic attack occurs in the position where the partial positive charge of the formed cation is the greatest, namely, on the anomeric carbon atom (C1). This regioselectivity leads exclusively to the formation of 2,3-unsaturated glycosides. Rare cases of substitution at the C3 carbon atom occur when using soft nucleophiles, e.g., RSH, N_3_^–^.^47^

The second considered pathway of the Ferrier rearrangement (Scheme 3B) assumes that the departure of the 3-OAc group is anchimerically assisted by the attack of the neighboring 4-OAc group. In this case, the mechanism involves a combination of two nucleophilic substitutions. The first one is an S_N_2 substitution where the trans-oriented 4-OAc group acts as a nucleophile, pushing out the 3-OAc group to form a dioxolenium ion. In the second step of this pathway of the Ferrier rearrangement, the dioxolenium ion should undergo an S_N_2′ substitution where the nucleophile attacks the less hindered C1 carbon atom. Importantly, anchimeric assistance requires the 3-OAc and 4-OAc groups in glycal to be antiperiplanarly oriented, which excludes the possibility of such assistance in the cases of acetylated galactal, fucal, and arabinal.

#### 2.2.2 Influence of the Glycal Structure on the Course of the Reaction

Regardless of which mechanism of the Ferrier rearrangement (Scheme 3) is more likely, it is the first stage of the reaction that determines its rate. This step involves breaking of the C3–O3 bond and may be influenced by the structure of the glycal.

**Figure 1:**
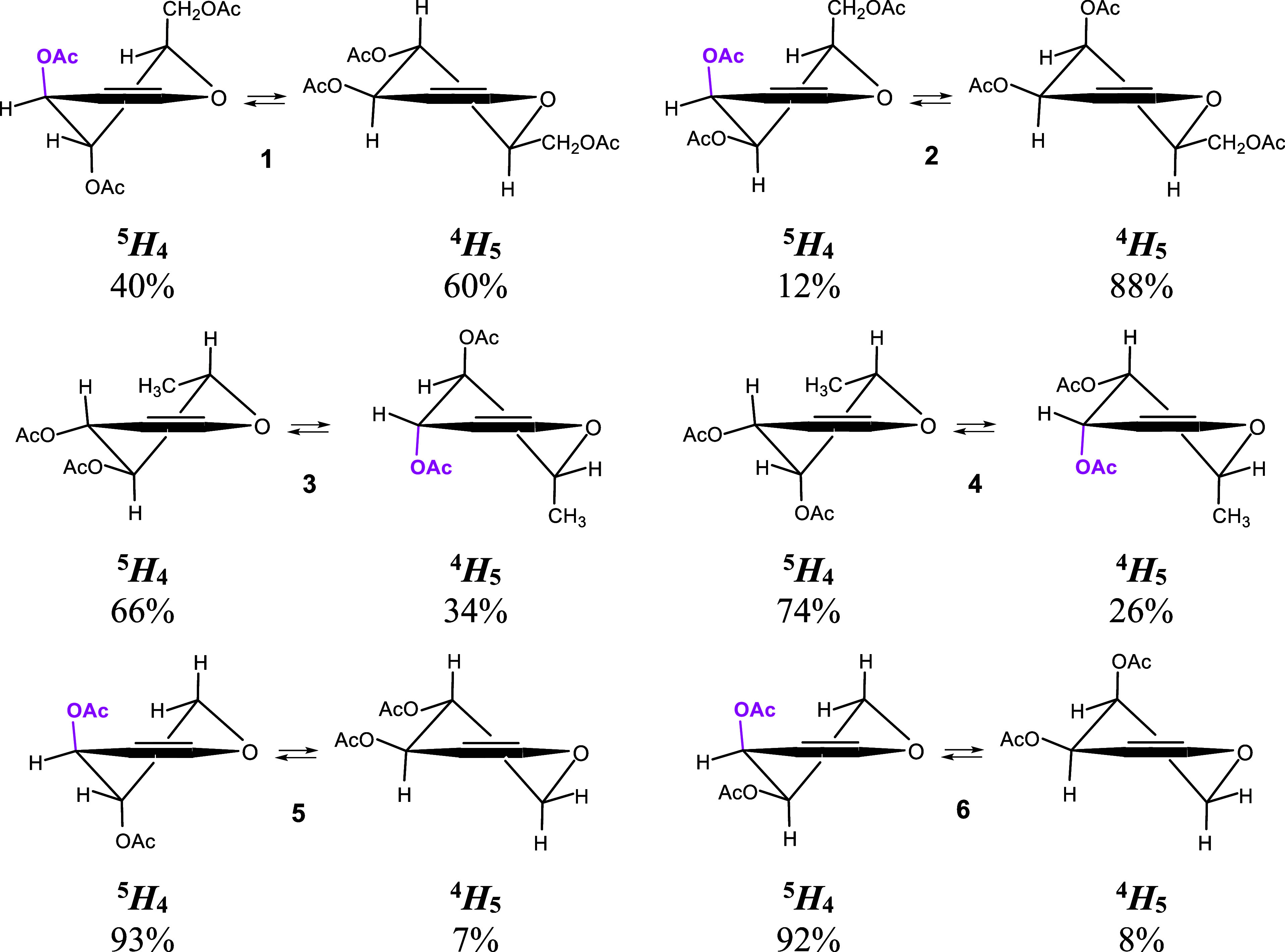
Contribution of the respective half-chair form to the conformational equilibrium of glycals **1**–**6**. The pseudoaxial orientation of the 3-OAc group illustrating the VAE is marked in purple.

The double bond of glycals, positioned between the C1 and C2 carbon atoms of the pyranoid ring, forces the half-chair conformation (H), where the O5, C1, C2, and C3 atoms lie in one plane. The remaining two ring atoms (C4 and C5) are located either above (superscript) or below (subscript) this plane. This means that glycals remain in the ^4^H_5_ ⇄ ^5^H_4_ conformational equilibrium (Figure 1).

**Figure 2:**
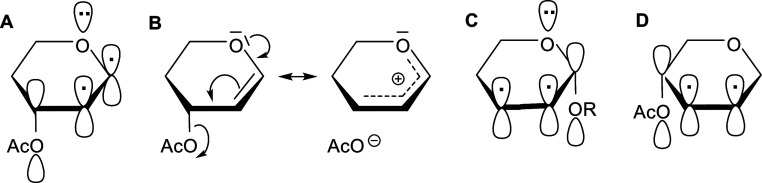
Overlapping orbitals in acetylated glycals resulting in the VAE (A). Resonance structures result from the VAE (B). Overlapping orbitals in 2,3-unsaturated glycosides result in anomeric and allylic effects (C). Overlapping orbitals in 2,3-unsaturated glycosides result in the allylic effect (D).

The vinylogous anomeric effect (VAE), independently introduced by Curran and Suh^48^ and Denmark and Dappen,^49^ is an important factor influencing the conformational equilibrium of acetylated glycals.^50,51^ Actually, this is an anomeric effect extended to the C3 carbon atom via a double bond. Thus, VAE favors the pseudoaxial orientation of the allylic ester group at the C3 carbon in acetylated glycals, and it is rationalized by the n → π → σ* delocalization of electrons (Figure 2A). Importantly, VAE not only stabilizes a given glycal conformation (Figure 1) but it also weakens the C3–O3 bond (Figure 2B), facilitating its dissociation and thereby promoting the Ferrier rearrangement.^52^ The ease of undergoing the Ferrier rearrangement is indeed correlated with the population of conformations in which the VAE operates. The populations of the respective conformations for glycals (Figure 1) were established based on the ^1^H NMR spectra for **1** and **2**(53) and based on the DFT calculations for **3**–**6**.^51^ Comparing the stereoisomers in pairs, glucal (**1**) with a population of 40% of the conformer is one in which VAE operates reacts more readily than galactal (**2**) with a population of 12%. Similarly, rhamnal (**3**) with 34% population reacts faster than fucal (**4**) with a population of 26%. Both xylal (**5**) and arabinal (**6**) reacted equally easily with our reaction, and both adopt mainly the conformation in which VAE operates (93% and 92%, respectively).

An appropriate glycal structure is undoubtedly necessary to enable anchimeric assistance (Scheme 3A). Stereodirecting participation of the ester neighboring group has a rich history concerning the typical glycoside synthesis.^54^ In the case of the Ferrier rearrangement, the occurrence of anchimeric assistance is cited to explain why acetylated glucal undergoes reaction more readily than the acetylated galactal.^55^ However, it is evident that not all glycals require such assistance from the 4-OAc group for effective Ferrier rearrangement.^37,56^ The Ferrier rearrangements presented herein also do not fully confirm the involvement of the anchimeric assistance in the course of the reaction mechanism. Such anchimeric assistance would be expected in the cases of acetylated d-glucal (**1**), l-rhamnal (**3**), and d-xylal (**5**), which possess trans-oriented 3-OAc and 4-OAc groups (Figure 1). Indeed, reactions under identical conditions proceed more rapidly for **1**, **3**, and **5** compared to **2** and **4**. However, there was no difference in the reaction time between acetylated d-xylal (**5**) and l-arabinal (**6**); both were completed within 5 min, suggesting that anchimeric assistance does not determine these reaction times. This conclusion, however, remains uncertain, as we did not examine the kinetics of these reactions minute by minute.

#### 2.2.3 Influence of the Intermediate’s Structure on the Course of the Reaction

Two different intermediates are considered in the Ferrier rearrangement (Scheme 3): the allyloxycarbenium ion and the dioxolenium ion. The allyloxycarbenium ion derived from acetylated glucal was identified and fully characterized by using a superacid.^57^ In turn, respective dioxolenium ions were identified when both acetylated glucal and galactal were subjected to nanoelectrospray ionization followed by in-source fragmentation of sodiated glycal precursor ions.^58^ Meanwhile, anchimeric assistance is impossible in the case of the acetylated galactal, where the 3-OAc and 4-OAc groups are cis-oriented. Therefore, the interconversion of the allyloxycarbenium ion into the dioxolenium ion is the only explanation for the formation of the latter in the case of the acetylated galactal. This equilibrium state means that the formation of the dioxolenium ion is not synonymous with anchimeric assistance.

**Figure 3:**
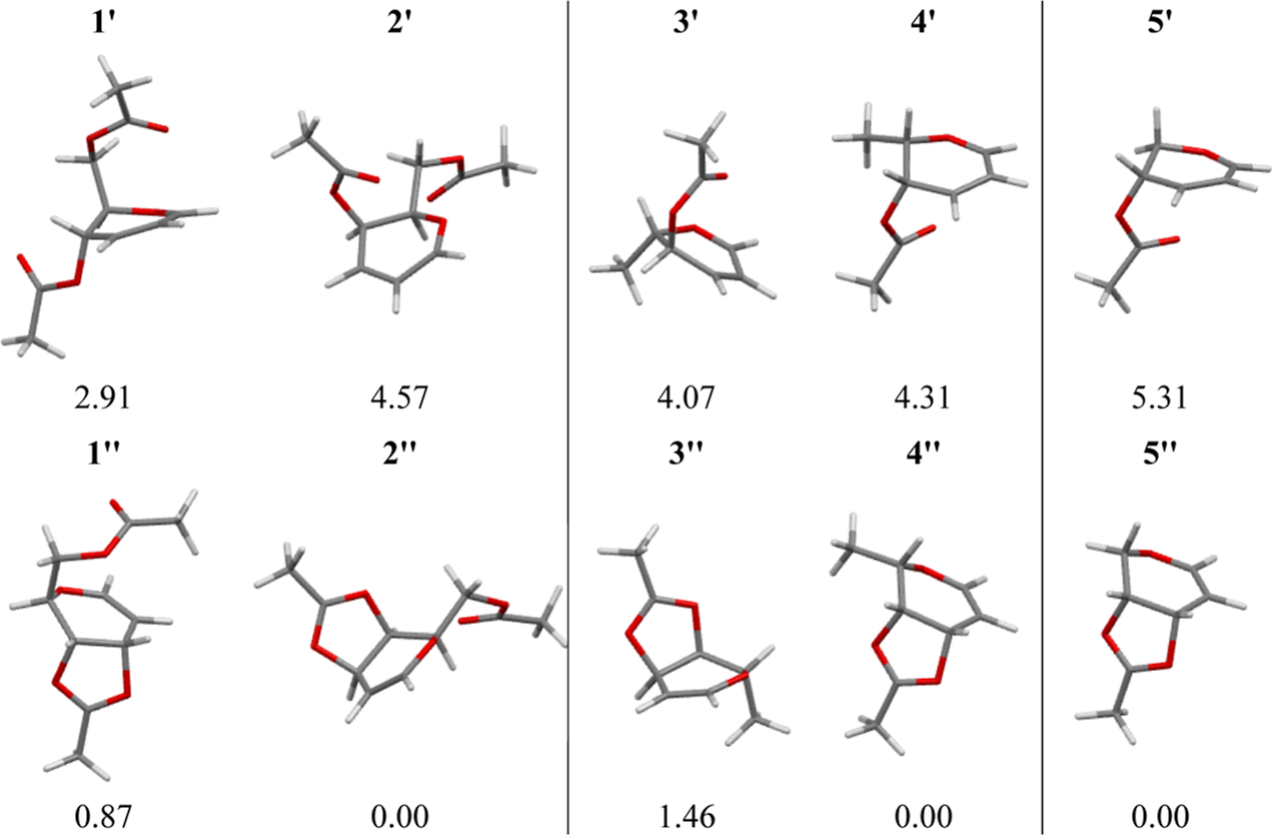
Lowest-energy structures of the allyloxycarbenium (**1′–5′**) and the dioxolenium ions (**1″–5″**) and their relative Gibbs free energies [kcal/mol], related to the most stable form within a given group of stereoisomers.

To gain knowledge about the stability of the intermediate products of the Ferrier rearrangement, both the allyloxycarbenium ions and the dioxolenium ions derived from glycals **1**–**5** were optimized by using DFT methods. Glycal **6** was excluded from these calculations because the intermediates formed from it are enantiomers of the analogous intermediates formed from glycal **5**. All possible rotations of the exocyclic substituents in both ions and in both the ^5^H_4_ and ^4^H_5_ conformations were considered in these calculations. The optimized, lowest-energy structures of the allyloxycarbenium ions (**1′–5′**) and the dioxolenium ions (**1″–5″**) along with their relative Gibbs free energies are presented in Figure 3. Cartesian coordinates of the optimized structures are given in Table S3.

According to the results of DFT calculations (Figure 3), all dioxolenium ions (**1″–5″**) are more stable than their allyloxycarbenium counterparts (**1′–5′**), the difference being 2.04 (**1″**/**1′**), 4.57 (**2″**/**2′**), 2.61 (**3″**/**3′**), 4.31 (**4″**/**4′**), and 5.31 (**5″**/**5′**) kcal/mol. However, the most stable dioxolenium ions cannot be formed directly from acetylated galactal (**2**), fucal (**4**), nor arabinal (**6**). In these cases, if the dioxolenium ion exists in the reaction mixture, it is a product of the transformation of the respective allyloxycarbenium ion (Scheme 3). This process is justified by the greater stability of the dioxolenium ion compared to that of the allyloxycarbenium ion. An analogous state of equilibrium was postulated between the acyloxonium ion (product of the anchimeric assistance) and oxacarbenium ion (product of the glycosidic bond dissociation) in the proposed mechanism of acid hydrolysis of methyl glucosides.^59^ The question is whether the dioxolenium ion can react with diosgenin. Formally, it would be a nucleophilic substitution of the S_N_2′ type, which is possible in the case of steric hindrance at the allylic C3 carbon atom. However, such a reaction requires a strong nucleophile, while diosgenin is not one. The S_N_1′ mechanism seems more likely in this situation. According to this mechanism, in the first stage, the C3–O3 bond in the dioxolenium ion dissociates, leading to the formation of the allyloxycarbenium ion, which is attacked by the nucleophile in the second stage. Thus, the presence of the allyloxycarbenium ion seems inevitable and necessary for Ferrier rearrangement to occur, although the existence of the dioxolenium ion in the reaction mixture cannot be excluded. On the other hand, dioxolenium ions may not form at all in the case of acetylated galactal (**2**), fucal (**4**), and arabinal (**6**) if it is assumed that the rate constant of glycoside formation is higher than the rate constant of the allyloxycarbenium ion transformation into the dioxolenium ion (*k*_4_ > *k*_3_).

#### 2.2.4 Influence of the Product’s Structure on the Course of the Reaction

**Scheme 4:**
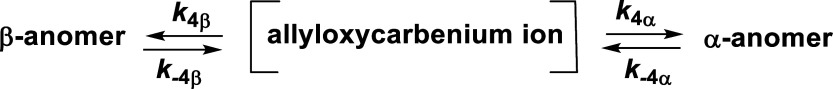
Equilibrium of the Anomerization in the Ferrier Rearrangement

The attack of the nucleophile on the C1 carbon atom in the intermediate product of the Ferrier rearrangement led to the formation of the α anomer and/or the β anomer of the 2,3-unsaturated glycoside (Scheme 4). As shown below, the ratio of the two anomers formed is closely correlated with their conformational stability.

**Figure 4:**
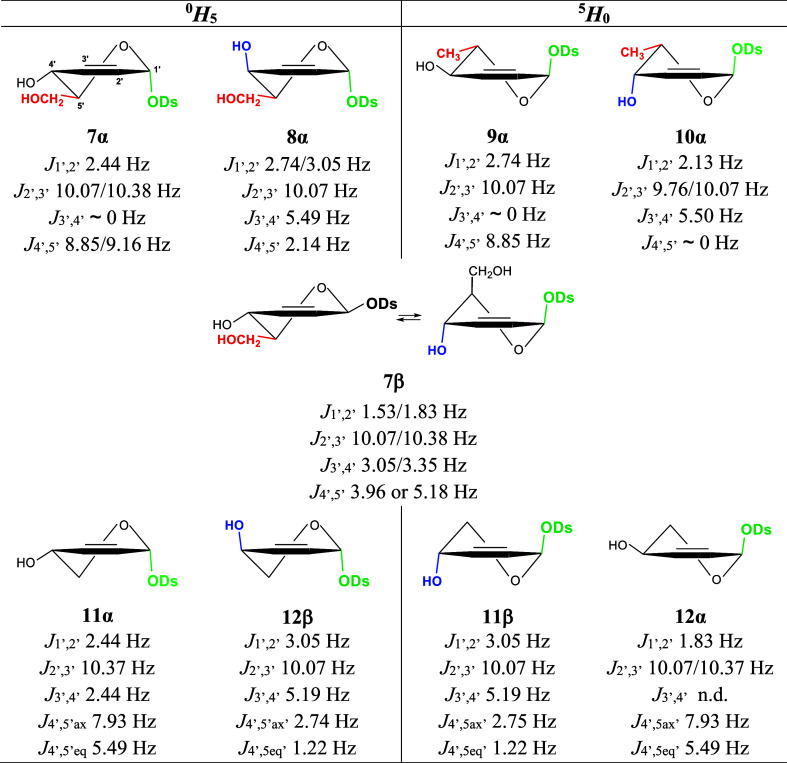
Conformations adopted by **7**–**12** and their characteristic coupling constants. Factors stabilizing conformation: the equatorial orientation of the terminal group (red), the anomeric effect (green), and the allylic effect (blue). The slash “/” separates two recorded values for the same coupling constant (the digital resolution of the ^1^H NMR spectra is 0.305 Hz/point). Subscripts “ax” and “eq” mean axial and equatorial protons, respectively.

The double bond in the Ferrier rearrangement products causes the C1, C2, C3, and C4 carbon atoms to lie in one plane. Therefore, 2,3-unsaturated glycosides adopt the ^0^H_5_ or ^5^H_0_ half-chair conformation (Figure 4). The conformational stability of such a sugar ring depends on three factors. First, to avoid unfavorable 1,3 pseudodiaxial interactions, the molecule tends to favor the equatorial orientation of the terminal group (red in Figure 4). Second, due to the anomeric effect (Figure 2C), a molecule tends to favor the pseudoaxial orientation of the aglycone (green in Figure 4). Third, according to the allylic effect, introduced and named by Ferrier and Sankey,^60^ the allylic ester group in 2,3-unsaturated pyranoses favors the pseudoaxial orientation (Figure 2D). As proved below, not only the allylic ester group but also the allylic hydroxyl group prefers the pseudoaxial orientation (blue in Figure 4). Probably, the allylic effect also applies to the pseudoaxial orientation of the aglycone and overlaps with the anomeric effect (Figure 2C).

All favorable factors mentioned above are found in the ^0^H_5_ conformation identified for **8α** and in the ^5^H_0_ conformation identified for **10α** (Figure 4). These 2,3-unsaturated glycosides were formed by the reactions of per-*O*-acetyl-d-galactal (**2**) and per-*O*-acetyl-d-fucal (**4**), respectively. The accumulation of favorable factors makes the α glycosides **8α** and **10α** particularly stable and causes the equilibrium of the anomerization (Scheme 4) to be clearly shifted toward them. It is worth noting that galactal (**2**) and fucal (**4**) react more slowly than other glycals (kinetic aspect), but reactions involving them are highly stereoselective and lead to the thermodynamically most stable products.

It appears that the equatorial orientation of the terminal group and the pseudoaxial orientation of the aglycone are enough to make the half-chair conformation stable. This statement concerns the ^0^H_5_ conformation identified for **7α** and the ^5^H_0_ conformation identified for **9α**. Due to this conformational stability, the equilibrium between the formed anomers in the reaction of glucal (**1**) and rhamnal (**3**) with diosgenin is again shifted toward the α anomer (**7α** and **9α**), but not as strongly as in the case of galactal (**2**) and fucal (**4**). Small amounts of **7β** are also obtained in the reaction of glucal (**1**) with diosgenin. This anomer, however, is not thermodynamically favored because it is conformationally unstable. In the ^0^H_5_ conformation of **7β**, the terminal group is oriented favorably, but the aglycone and 4-OH groups are oriented unfavorably. It is exactly the opposite in the ^5^H_0_ conformation of **7β**. None of the half-chair conformations of **7β** are stable enough to be fully adopted. Therefore, **7β** remains in the ^0^H_5_ ⇄ ^5^H_0_ conformational equilibrium, which was proven based on its ^1^H NMR spectrum (see Section 2.3). The fact that **7β** is in conformational equilibrium indicates that the equatorial orientation of the terminal hydroxymethyl group is the most important factor in determining the conformational preference of 2,3-unsaturated glycosides. This effect in the ^0^H_5_ conformation of **7β** is offset by both the anomeric effect and the allylic effect in the ^5^H_0_ conformation of **7β**. The lack of this important factor in **11** and **12** completely changes the anomeric preferences in the Ferrier rearrangement in the case of acetylated xylal (**5**) and arabinal (**6**). The products of their reactions are dominated by β anomers, which can adopt a conformation where the other two favorable effects occur. This is the ^5^H_0_ conformation in the case of **11β** and the ^0^H_5_ conformation in the case of **12β** (it should be added that the sugar parts of **11β** and **12β** are mirror images). The α configuration of 2,3-unsaturated glycosides **11α** and **12α**, unlike the β configuration of **11β** and **12β**, does not allow for the aglycone and 3-OH groups to be pseudoaxially oriented at the same time. As a result, the α anomers are thermodynamically less stable than the β anomers, which shifts the anomerization equilibrium in favor of the latter. The fact that α anomers adopt a conformation in which the aglycone is pseudoaxially oriented may mean that the anomeric effect is stronger than the allylic effect caused by the 3-OH group. It is also possible that both the anomeric and allylic effects act in the pseudoaxial arrangement of the aglycone. On the other hand, the fact that the β anomers (**11β** and **12β**) dominate over the α anomers (**11α** and **12α**) in the products of reaction of xylal (**5**) and arabinal (**6**) is evidence of the allylic effect action caused by the 3-OH group.

### 2.3 Identification of the ^0^H_5_ and ^5^H_0_ Conformations

The *J*_4′,5′_ coupling constant is the most diagnostic for distinguishing between the ^5^H_0_ and ^0^H_5_ conformations. If the *J*_4′,5′_ is in the range of 8–9 Hz, it means that the H5 proton is axially oriented and the H4 proton is pseudoaxially oriented. Such a situation takes place in the ^0^H_5_ conformation of **7α** and the ^5^H_0_ conformation of **9α** (Figure 4). The *J*_3′,4′_ ∼ 0 Hz recorded for **7α** and **9α** is an additional confirmation of the pseudoaxial orientation of the H4 proton. This value means that the H3–C3–C4–H4 torsion angle is close to 90°. In the half-chair conformations where the C1, C2, H2, C3, H3, and C4 atoms are arranged in one plane, such a value of the torsion angle occurs with the pseudoaxial orientation of the H4 proton. The pseudoequatorial orientation of the H4 proton is in accordance with the allylic effect and takes place in the most stable conformations, i.e., the ^0^H_5_ for **8α** and the ^5^H_0_ for **10α**. In these cases, the *J*_4′,5′_ coupling constants are 2.14 or close to zero. These values, in particular *J*_4′,5′_ ∼ 0 Hz, clearly confirm the conformation assigned to compounds **8α** and **10α** because these are characteristic of the antiperiplanar orientation of the H4 proton and the ring oxygen atom.^61^ In turn, *J*_3′,4′_ = 5.49 Hz is typical for a torsion angle of about 10°.^62^

There is no terminal group on the C5 carbon atom in pentopyranoses; thus, two different *J*_4′,5′_ coupling constants are recorded for them. The larger constant is for the axial H5 proton, and the smaller one is for the equatorial H5 proton. If one of these constants is 7.93 Hz and the other is 5.49, then the H4 proton is pseudoaxially oriented. This situation occurs in the ^0^H_5_ conformation of **11α** and the ^5^H_0_ conformation of **12α**. If one of these constants is 2.75 Hz and the other is 1.22 Hz, then the H4 proton is pseudoequatorially oriented. This situation takes place in the ^5^H_0_ conformation of **11β** and the ^0^H_5_ conformation of **12β**. The coupling constant *J*_3′,4′_ = 5.19 Hz additionally confirms the pseudoequatorial orientation of the H4 proton in **11β** and **12β**.

The coupling constants recorded for **7β**, particularly *J*_3′,4′_ = 3.05/3.35 Hz and *J*_4′,5′_ = 3.96 or 5.50 Hz, indicate the conformational equilibrium of the 2,3-unsaturated sugar ring.

### 2.4 Cytotoxic Activity

The MTT assay was performed to specify the cytotoxicity of **7**–**12** and **13**–**18** against prostate cancer cells (PC3 line), breast cancer cells (MCF7 line), and human keratinocytes (HaCaT line). The mentioned cell lines were assayed by exposing them for 48 h to a medium containing the studied compounds over a wide range of concentrations. The viabilities of PC3 (A), MCF7 (B), and HaCaT (C) cells with the tested compounds are presented in Figure S16. The IC_50_ values (dose of the compound which caused a 50% reduction of cell viability) and the selectivity indexes (SI), which show the ratio of IC_50_ for normal cell line to IC_50_ for cancer cell line, are shown in Table S2.

The majority of the presented saponins, both 2,3-unsaturated (**7**–**12**) and 2,3-dideoxy (**13**–**18**), are effectively active against PC3 cancer cells at concentrations above 18–19 μM (Figure S16). The effective action of these saponins on MCF7 cancer cells requires rather high concentrations. The 2,3-unsaturated diosgenyl glycoside with the α-d-*erythro* configuration (**7α**), which is derived from d-glucal (**1**) and adopts the ^0^H_5_ conformation, is the most active saponin against PC3 cancer cells. The respective IC_50_ value for **7α** is 16.84 μM (Table S2). This means that **7α** exhibits larger cytotoxicity against PC3 cancer cells than diosgenyl β-d-glucopyranoside (**7β**) and diosgenin alone, both tested on cervical HeLa and CaSki cancer cells.^31^ Importantly, saponin **7α** has a slightly stronger effect on PC3 cancer cells than on the healthy cells tested (SI = 1.20).

Among the 2,3-unsaturated diosgenyl glycosides, saponin **12β** with β-l-*glycero* configuration and the ^0^H_5_ conformation exhibits the best selectivity (SI 2.08). It has relatively good activity against PC3 cells (IC_50_ = 27.85 μM). The selectivity of **12β** is twice as high as the selectivity of **8α**. The latter saponin differs from the former only in the presence of a terminal hydroxymethyl group. It seems that the lack of a terminal hydroxymethyl group is a good predictor of the selectivity of the anticancer effect of saponins. Those that have a favorable SI value (Table S2) for both cancer cell lines (ca. 2) have hydrogen (**12β**, **18β**) or methyl (**15α**) in place of the CH_2_OH group.

The configuration of the anomeric carbon atom of 2,3-unsaturated saponin **11** influences its anticancer activity. While the α anomer (**11α**) exhibits activity against the PC3, MCF7, and HaCaT cell lines at the IC_50_ level of 24.44, 40.59, and 24.48 μM, respectively, the β anomer (**11β**) is completely inactive in the case of all tested cell lines. This completely inactive saponin **11β** is a diastereoisomer of saponin **12β**, which is active against the tested cell lines at the IC_50_ level of 27.85, 35.61, and 57.87 μM, respectively. In diastereoisomers **11β** and **12β**, the sugar parts are mirror images. This means that the absolute configuration (d or l) of the sugar part is relevant to the saponin activity. This situation changes when we consider the analogous anomers of diosgenyl 2,3-dideoxy glycosides (**17**). In their case, the β anomer (**17β**) is definitely more active against all tested cell lines than the α anomer (**17α**). In turn, the pair of diastereoisomers **17β** and **18β**, in which the sugar parts are mirror images, do not differ significantly in activity toward the tested cancer cell lines. The presented results indicate that the presence or absence of a double bond, which, among other things, determines the shape of the pyranose ring, half-chair in the case of **11**, and chair in the case of **17**, has a significant impact on the activity of the tested compounds.

### 2.5 Hemolytic Activity

Regardless of numerous pharmacological properties, saponins are known to induce hemolysis, which limits their potential.^63^ Therefore, the hemolytic activities of compounds **7**–**12** and **13**–**18** were determined. The results revealed that the tested compounds have no hemolytic activity. The level of hemolysis for samples exposed to the compounds at the highest concentrations was not higher than 1% as compared to the positive controls.

### 2.6 Antimicrobial Activity

2,3-Unsaturated (**7**–**12**) and 2,3-dideoxy (**13**–**18**) diosgenyl glycosides were tested for their in vitro antimicrobial activity against seven strains of Gram-positive bacteria and four strains of Gram-negative bacteria. The measured minimum inhibitory concentration (MIC) values for G(+) bacteria are summarized in Table S2. The MIC values for G(−) bacteria are not presented because these are >256 μg/mL in each case, which means a lack of activity at the highest tested concentration.

Lack of activity against G(−) bacteria is typical for diosgenyl glycosides. None of the diosgenyl glycosaminosides previously tested by us showed such activity.^64−66^ However, it is surprising that **7**–**12** and **13**–**18** show such low activity against G(+) bacteria (Table S2). In the best cases, some of the 2,3-unsaturated saponins (**7**–**12**) are active against some of the bacterial strains and cultures used only when the compounds are applied at the highest tested concentration (256 μg/mL). The same can be said about 2,3-dideoxy saponins (**13**–**18**), although compounds **14α** and **17α** are an exception here. These inhibit the growth of the *Staphylococcus aureus* strains tested, including methicilin-resistant *S. aureus* (ATCC codes 43,300 and 33,591). The antistaphylococcal activity was achieved when **14α** and **17α** were applied at concentrations of 64–256 and 128–256 mg/L, respectively (Table S2). *S. aureus* is one of the most health-threatening bacteria due to its ability to develop resistance to antimicrobials and form biofilms on biomaterials. The results obtained for compounds **14α** and **17α** encourage further study of their antistaphylococcal activity.

## 3 Summary

This work presents an efficient and stereoselective synthesis of 2,3-unsaturated diosgenyl glycosides mainly with the atypical α configuration of the anomeric carbon atom. These saponins were subsequently chemoselectively hydrogenated to 2,3-dideoxy diosgenyl glycosides.

The configurational diversity of the used glycals and obtained 2,3-unsaturated glycosides enabled an in-depth discussion of the Ferrier rearrangement mechanism. This discussion led to the following conclusions: (1) the VAE in glycals may have a beneficial impact on the reaction course. (2) Although the structure of glucal, rhamnal, and xylal allows for anchimeric assistance, the latter is not necessary for the formation of the dioxolenium ion. (3) The intermediate product of the Ferrier rearrangement represents an equilibrium mixture of the dioxolenium ion and allyloxycarbenium ion, shifted in favor of the former. (4) It is the allyloxycarbenium ion that undergoes the farther reaction with the nucleophile. (5) The high stereoselectivity of the Ferrier rearrangement is clearly correlated with the conformational stability of the products. (6) Factors influencing this stability are ranked as the equatorial orientation of the terminal group > pseudoaxial orientation of the aglycone > pseudoaxial orientation of the 3-OH group.

The new type of saponins presented exhibits moderate anticancer activity and almost no antibacterial activity. The structure–activity relationship discussion indicates the following: (1) the absence of a terminal hydroxymethyl group in a sugar moiety is a good predictor of cytotoxic selectivity. (2) The anomeric carbon atom configuration influences anticancer activity. (3) Both the configuration and conformation of the pyranose ring affect the activity of the tested saponins.

## 4 Experimental Section

### 4.1 Synthetic Procedures and Results of Analysis

#### 4.1.1 General Methods

Solvents and chemical reagents were purchased and used without further purification. The ^1^H and ^13^C NMR spectra were recorded on the Bruker Avance III 500 MHz (500.13/125.76 MHz), using CDCl_3_ as a solvent with internal Me_4_Si. Structural assignments were made with additional information from COSY and HSQC experiments. The digital resolution of the ^1^H NMR spectra was 0.305 Hz/point. Positive-ion mode MALDI-TOF mass spectra were obtained using a Bruker Biflex III spectrometer with 4-cyano-4-hydroxycinnamic acid or 2,5-dihydroxybenzoic acid matrixes. High-resolution mass spectrometry (HRMS) spectra were recorded using a Nexera X2 ultra high performance liquid chromatographyinstrument coupled to a Triple TOF 5600+ (SCIEX) mass spectrometer equipped with a duoelectrospray interface, operated in the negative ionization mode as the monoisotopic formate adducts. Thin-layer chromatography (TLC) was performed on aluminum plates coated with E. Merck Kieselgel 60 F_254_, using the following eluent systems (v/v): A, 2:1 toluene/AcOEt; B, 6:1 toluene/AcOEt; C, 9:1 toluene/AcOEt; D, 1:2 toluene/AcOEt; E, 2:1 *n*-heptane/AcOEt; F, 4:1 *n*-heptane/AcOEt. For the compounds detection, the dry plates were sprayed with 1% H_2_SO_4_ in MeOH and heated at ca. 200 °C in a stream of hot air. Filtration through silica gel was performed on MN Kieselgel 60 (<0.08 mm). Column flash chromatography was conducted using a PuriFlash 450 instrument with a UV-DAD detector on PuriFlash SI-HP 50 μm columns, using one of the above-mentioned eluent systems.

#### 4.1.2 Per-*O*-acetylated Glycals (**1–6**)

These were obtained according to the known procedures in which the bromoacetate of the respective sugar was subjected to a reductive elimination reaction (Zn, AcOH, CuSO_4_).^39^

#### 4.1.3 2,3-Unsaturated Diosgenyl Glycosides (**7–12**)

General procedure: Diosgenin (1.0–1.1 mmol) and the respective per-*O*-acetylated glycal (**1**–**6**) (1.0 mmol) were dissolved in a mixture of anhydrous Et_2_O and DCM (2:1) with the addition of freshly dried, ground molecular sieves 4 Å. Next, anhydrous FeCl_3_ was added (0.05–0.1 mmol). The mixture was stirred at rt for 5–15 min and monitored with TLC (eluent A). Then, the reaction mixture was filtered through silica gel under reduced pressure using DCM as an eluent. Next, the solvents were evaporated, and the residue was dissolved in an anhydrous solution of MeONa (0.3–0.5 mmol) in MeOH (35 mL). The mixture was stirred at rt for 0.5–3 h, monitored with TLC (eluent A), and finally passed through silica gel using CHCl_3_ as an eluent. The solvents were evaporated, and the residue was purified using flash chromatography (eluent B or C).

##### 4.1.3.1 Diosgenyl 2,3-Dideoxy-α-d-*erythro*- (**7α**) and -β-d-*erythro*-Hex-2-enopyranoside (**7β**)

Reaction of diosgenin (0.85 g, 2.05 mmol) with **1** (0.51 g, 1.87 mmol) followed by flash chromatography (eluent B) gave first **7α** (0.29 g, 29%, a white amorphous solid); *R*_*f*_ 0.40 (eluent D); ^1^H NMR (CDCl_3_, 500 MHz): δ 5.96 (d, 1H, *J* = 10.07 Hz, H3′), 5.75 (dt, 1H, *J* = 10.07/10.38 Hz, *J* = 2.44 Hz, H2′), 5.35 (d, 1H, *J* = 5.18 Hz, H6), 5.19 (br s, 1H, H1′), 4.41 (q, 1H, *J* = 7.32/7.63 Hz, H16), 4.22 (d, 1H, *J* = 8.85/9.16 Hz, H4′), 3.87 (br, 2H, 2 × H6′), 3.77 (dt, 1H, *J* = 8.85/9.16 Hz, *J* = 4.27/4.58 Hz, H5′), 3.53 (tt, 1H, *J* = 10.98 Hz, *J* = 5.19 Hz, H3), 3.47 (ddd, 1H, *J* = 10.98 Hz, *J* = 4.27 Hz, *J* = 1.83 Hz, H26_a_), 3.38 (t, 1H, *J* = 10.98 Hz, H26_b_), 2.38 (ddd, 1H, *J* = 13.13 Hz, *J* = 4.89 Hz, *J* = 1.52 Hz, H4_a_), 2.34 (tq, 1H, *J* = 13.13 Hz, *J* = 2.14/2.44 Hz, H4_b_), 2.00 (m, 1H, H7_a_), 1.98 (ddd, 1H, *J* = 12.51 Hz, *J* = 7.32 Hz, *J* = 5.80 Hz, H15_a_), 1.90 (m, 1H, H2_a_), 1.86 (m, 2H, H1_a_, H20), 1.78 (dd, 1H, *J* = 8.54 Hz, *J* = 7.02 Hz, H17), 1.74 (dt, 1H, *J* = 12.21 Hz, *J* = 3.05/3.35 Hz, H12_a_), 1.68 (m, 1H, H23_a_), 1.64 (m, 1H, H25), 1.63 (m, 1H, H8), 1.62 (m, 1H, H24_a_), 1.60 (m, 1H, H23_b_), 1.53 (m, 1H, H7_b_), 1.52 (m, 1H, H2_b_), 1.51 (m, 1H, H11_a_), 1.47 (m, 1H, H11_b_), 1.45 (dt, 1H, *J* = 12.82 Hz, *J* = 4.57 Hz, H24_b_), 1.29 (ddd, 1H, *J* = 13.73 Hz, *J* = 11.90 Hz, *J* = 6.41 Hz, H15_b_), 1.18 (td, 1H, *J* = 12.82 Hz, *J* = 4.58 Hz, H12_b_), 1.10 (ddd, 1H, *J* = 13.74 Hz, *J* = 10.68 Hz, *J* = 5.49 Hz, H14), 1.04 (m, 1H, H1_b_), 1.02 (s, 3H, H19), 0.97 (d, 3H, *J* = 7.02 Hz, H21), 0.94 (dd, 1H, *J* = 11.29/11.60 Hz, *J* = 4.88/5.19 Hz, H9), 0.79 (s, 3H, H18), 0.79 (d, 3H, *J* = 4.58 Hz, H27); ^13^C{^1^H} NMR (CDCl_3_, 125 MHz): δ 140.8 (C5), 133.0 (C3′), 127.0 (C2′), 121.6 (C6), 109.3 (C22), 92.6 (C1′), 80.8 (C16), 77.9 (C3), 71.3 (C5′), 66.9 (C26), 64.5 (C4′), 63.0 (C6′), 62.1 (C17), 56.5 (C14), 50.1 (C9), 41.6 (C20), 40.5 (C13), 40.3 (C4), 39.8 (C12), 37.1 (C1), 36.8 (C10), 32.1 (C7), 31.8 (C15), 31.4 (C23, C25), 30.3 (C8), 28.8 (C24), 28.3 (C2), 20.8 (C11), 19.4 (C19), 17.1 (C27), 16.3 (C18), 14.5 (C21); MALDI-TOF-MS *m*/*z*: [M + H]^+^ calcd for C_33_H_51_O_6_, 543.4; found, 543.4, [M + Na]^+^ calcd for C_33_H_50_O_6_Na, 565.4; found, 565.4, [M + K]^+^ calcd for C_33_H_50_O_6_K, 581.3; found, 581.5; HRMS (ESI) *m*/*z*: [M + HCOO]^−^ calcd for C_34_H_51_O_8_, 587.3584; found, 587.3577. Eluted second was the 7:1 mixture of **7α** and **7β** (0.57 g, 56%); *R*_*f*_ 0.30 (solvent D) for **7β**; ^1^H NMR for sugar part of **7β** (CDCl_3_, 500 MHz): δ 6.02 (ddd, 1H, *J* = 10.07/10.38 Hz, *J* = 3.05/3.35 Hz, *J* = 1.53/1.83 Hz, H3′), 5.78 (dt, 1H, *J* = 10.07/10.38 Hz, *J* = 1.53/1.83 Hz, H2′), 5.27 (q, 1H, *J* = 1.53/1.83 Hz, H1′), 4.19 (br, 1H, H4′), 3.80 (dd, 1H, *J* = 10.68 Hz, *J* = 5.19 Hz, H6_b_), 3.74 (td, 1H, *J* = 5.18 Hz, *J* = 3.96 Hz, H5′).

##### 4.1.3.2 Diosgenyl 2,3-Dideoxy-α-d-*threo*-hex-2-enopyranoside (**8α**)

Reaction of diosgenin (0.78 g, 1.88 mmol) with **2** (0.49 g, 1.78 mmol) followed by flash chromatography (eluent B) provided **8α** (0.30 g, 31%, a white amorphous solid); *R*_*f*_ 0.30 (eluent D); ^1^H NMR (CDCl_3_, 500 MHz) δ 6.14 (dd, 1H, *J* = 10.07 Hz, *J* = 5.49 Hz, H3′), 5.91 (dd, 1H, *J* = 10.07 Hz, *J* = 3.05 Hz, H2′), 5.36 (d, 1H, *J* = 5.17 Hz, H6), 5.20 (d, 1H, *J* = 2.74 Hz, H1′), 4.41 (q, 1H, *J* = 7.33/7.63 Hz, H16), 4.13 (td, 1H, *J* = 5.50/5.19 Hz, *J* = 2.14 Hz, H5′), 3.97 (dd, 1H, *J* = 11.91 Hz, *J* = 5.49 Hz, H6_a_), 3.93 (dd, 1H, *J* = 5.50 Hz, *J* = 2.14 Hz, H4′), 3.89 (dd, 1H, *J* = 11.91 Hz, *J* = 4.88 Hz, H6_b_), 3.57 (tt, 1H, *J* = 10.98 Hz, *J* = 4.88 Hz, H3), 3.47 (ddd, 1H, *J* = 10.68 Hz, *J* = 4.27 Hz, *J* = 1.53 Hz, H26_a_), 3.38 (t, 1H, *J* = 10.99 Hz, H26_b_), 2.39 (ddd, 1H, *J* = 13.43 Hz, *J* = 4.88 Hz, *J* = 1.22 Hz, H4_a_), 2.33 (br t, 1H, H4_b_), 2.01 (m, 1H, H7_a_), 1.98 (ddd, 1H, *J* = 12.51 Hz, *J* = 7.63 Hz, *J* = 5.19 Hz, H15_a_), 1.89 (m, 1H, H2_a_), 1.87 (m, 1H, H20), 1.86 (m, 1H, H1_a_), 1.78 (dd, 1H, *J* = 8.54 Hz, *J* = 7.02 Hz, H17), 1.74 (dt, 1H, *J* = 12.21 Hz, *J* = 3.05/3.35 Hz, H12_a_), 1.69 (m, 1H, H23_a_), 1.63 (m, 1H, H8), 1.61 (m, 2H, H24_a_, H25), 1.60 (m, 1H, H23_b_), 1.54 (m, 1H, H7_b_), 1.52 (m, 1H, H11_a_), 1.50 (m, 1H, H2_b_), 1.47 (m, 1H, H11_b_), 1.45 (dt, 1H, *J* = 12.82 Hz, *J* = 3.97 Hz, H24_b_), 1.29 (ddd, 1H, *J* = 13.73 Hz, *J* = 12.21 Hz, *J* = 6.41 Hz, H15_b_), 1.18 (td, 1H, *J* = 12.51 Hz, *J* = 4.58 Hz, H12_b_), 1.11 (ddd, 1H, *J* = 13.74 Hz, *J* = 10.68 Hz, *J* = 5.49 Hz, H14), 1.05 (m, 1H, H1_b_), 1.02 (s, 3H, H19), 0.97 (d, 3H, *J* = 7.02 Hz, H21), 0.94 (dd, 1H, *J* = 11.60 Hz, *J* = 5.19 Hz, H9), 0.79 (s, 3H, H18), 0.79 (d, 3H, *J* = 5.19 Hz, H27); ^13^C{^1^H} NMR (CDCl_3_, 125 MHz): δ 140.7 (C5), 129.2 (C2′), 129.1 (C3′), 121.7 (C6), 109.3 (C22), 92.6 (C1′), 80.8 (C16), 77.5 (C3), 69.9 (C5′), 66.9 (C26), 63.0 (C6′), 62.9 (C4′), 62.1 (C17), 56.5 (C14), 50.0 (C9), 41.6 (C20), 40.4 (C13), 40.3 (C4), 39.8 (C12), 37.1 (C1), 36.8 (C10), 32.1 (C7), 31.8 (C15), 31.4 (C23, C25), 30.3 (C8), 28.8 (C24), 28.3 (C2), 20.8 (C11), 19.4 (C19), 17.1 (C27), 16.3 (C18), 14.5 (C21); MALDI-TOF-MS *m*/*z*: [M + H]^+^ calcd for C_33_H_51_O_6_, 543.4; found, 543.3, [M + Na]^+^ calcd for C_33_H_50_O_6_Na, 565.4; found, 565.3, [M + K]^+^ calcd for C_33_H_50_O_6_K, 581.3; found, 581.2; HRMS (ESI) *m*/*z*: [M + HCOO]^−^ calcd for C_34_H_51_O_8_, 587.3584; found, 587.3579.

##### 4.1.3.3 Diosgenyl 2,3,6-Trideoxy-α-l-*erythro*-hex-2-enopyranoside (**9α**)

Reaction of diosgenin (0.43 g, 1.04 mmol) with **3** (0.20 g, 0.93 mmol) followed by flash chromatography (eluent C) led to **9α** (0.29 g, 59%, a white amorphous solid); *R*_*f*_ 0.38 (eluent E); ^1^H NMR (CDCl_3_, 500 MHz): δ 5.92 (d, 1H, *J* = 10.07 Hz, H3′), 5.74 (ddd, 1H, *J* = 10.07 Hz, *J* = 2.74 Hz, *J* = 2.14 Hz, H2′), 5.34 (d, 1H, *J* = 5.49 Hz, H6), 5.09 (br s, 1H, H1′), 4.41 (q, 1H, *J* = 7.33/7.63 Hz, H16), 3.84 (br t, 1H, *J* = 7.62/8.85 Hz, H4′), 3.76 (dq, 1H, *J* = 8.85 Hz, *J* = 6.10 Hz, H5′), 3.54 (tt, 1H, *J* = 11.29 Hz, *J* = 4.58/4.88 Hz, H3), 3.47 (ddd, 1H, *J* = 10.68/10.99 Hz, *J* = 4.27 Hz, *J* = 1.83 Hz, H26_a_), 3.37 (t, 1H, *J* = 10.68/10.97 Hz, H26_b_), 2.34 (ddd, 1H, *J* = 13.12 Hz, *J* = 4.88 Hz, *J* = 2.14 Hz, H4_a_), 2.25 (tq, 1H, *J* = 11.29/13.74 Hz, *J* = 2.44/2.75 Hz, H4_b_), 1.99 (m, 1H, H7_a_), 1.98 (ddd, 1H, *J* = 12.20 Hz, *J* = 7.63 Hz, *J* = 5.49 Hz, H15_a_), 1.91 (dm, 1H, *J* = 12.81, H2_a_), 1.87 (m, 1H, H20), 1.85 (dt, 1H, *J* = 13.43 Hz, *J* = 3.36 Hz, H1_a_), 1.78 (dd, 1H, *J* = 8.55 Hz, *J* = 6.71 Hz, H17), 1.74 (dt, 1H, *J* = 12.51 Hz, *J* = 2.74/3.66 Hz, H12_a_), 1.69 (m, 1H, H23_a_), 1.63 (m, 2H, H8, H24_a_), 1.61 (m, 1H, H2_b_), 1.60 (m, 2H, H23_b_, H25), 1.54 (m, 1H, H7_b_), 1.52 (m, 1H, H11_a_), 1.47 (m, 1H, H11_b_), 1.46 (m, 1H, H24_b_), 1.39 (d, 1H, *J* = 8.54 Hz, OH), 1.31 (d, 1H, *J* = 6.10 Hz, H6′), 1.27 (m, 1H, H15_b_), 1.18 (td, 1H, *J* = 12.82 Hz, *J* = 4.58 Hz, H12_b_), 1.11 (ddd, 1H, *J* = 13.73 Hz, *J* = 10.37 Hz, *J* = 5.49 Hz, H14), 1.08 (m, 1H, H1_b_), 1.02 (s, 3H, H19), 0.97 (d, 3H, *J* = 7.02 Hz, H21), 0.95 (dd, 1H, *J* = 11.60 Hz, *J* = 5.19 Hz, H9), 0.79 (s, 3H, H18), 0.79 (d, 3H, *J* = 6.40 Hz, H27); ^13^C{^1^H} NMR (CDCl_3_, 125 MHz): δ 140.8 (C5), 133.2 (C3′), 127.3 (C2′), 121.5 (C6), 109.3 (C22), 92.8 (C1′), 80.8 (C16), 77.8 (C3), 69.8 (C4′), 67.9 (C5′), 66.9 (C26), 62.1 (C17), 56.5 (C14), 50.1 (C9), 41.6 (C20), 40.3 (C13), 39.8 (C12), 39.1 (C4), 37.4 (C1), 36.9 (C10), 32.1 (C7), 31.9 (C15), 31.4 (C23, C25), 30.3 (C8), 30.0 (C2), 28.8 (C24), 20.9 (C11), 19.4 (C19), 18.0 (C6′), 17.1 (C27), 16.3 (C18), 14.5 (C21); MALDI-TOF-MS *m*/*z*: [M + H]^+^ calcd for C_33_H_51_O_5_, 527.4; found, 527.4, [M + Na]^+^ calcd for C_33_H_50_O_5_Na, 549.4; found, 549.4, [M + K]^+^ calcd for C_33_H_50_O_5_K, 365.3; found, 565.4; HRMS (ESI) *m*/*z*: [M + HCOO]^−^ calcd for C_34_H_51_O_7_, 571.3635; found, 571.3626.

##### 4.1.3.4 Diosgenyl 2,3,6-Trideoxy-α-l-*threo*-hex-2-enopyranoside (**10α**)

Reaction of diosgenin (0.83 g, 2.00 mmol) with **4** (0.39 g, 1.82 mmol) followed by flash chromatography (eluent C) provided **10α** (0.43 g, 45%, a white amorphous solid); *R*_*f*_ 0.63 (eluent D); ^1^H NMR (CDCl_3_, 500 MHz): δ 6.17 (dd, 1H, *J* = 9.76 Hz, *J* = 5.49 Hz, H3′), 5.86 (dd, 1H, *J* = 9.77 Hz, *J* = 2.13 Hz, H2′), 5.34 (d, 1H, *J* = 3.66 Hz, H6), 5.11 (s, 1H, H1′), 4.41 (q, 1H, *J* = 7.33/7.63 Hz, H16), 4.17 (q, 1H, *J* = 6.41/6.72 Hz, *J* = 3.66 Hz, H5′), 3.58 (m, 1H, H4′), 3.54 (m, 1H, H3), 3.47 (bd, 1H, *J* = 10.68 Hz, H26_a_), 3.38 (t, 1H, *J* = 10.68/10.98 Hz, H26_b_), 2.35 (dd, 1H, *J* = 12.21 Hz, *J* = 4.58 Hz, H4_a_), 2.24 (bt, 1H, *J* = 11.90/12.21 Hz, H4_b_), 1.99 (m, 2H, H7_a_, H15_a_), 1.92 (m, 1H, H2_a_), 1.87 (m, 1H, H20), 1.86 (m, 1H, H1_a_), 1.78 (t, 1H, *J* = 7.63/7.94 Hz, H17), 1.74 (bd, 1H, *J* = 12.82 Hz, H12_a_), 1.69 (m, 1H, H23_a_), 1.64 (m, 1H, H25), 1.63 (m, 1H, H8), 1.62 (m, 1H, H24_a_), 1.60 (m, 1H, H23_b_), 1.58 (m, 1H, H2_b_), 1.55 (m, 1H, H7_b_), 1.51 (m, 1H, H11_a_), 1.47 (m, 1H, H11_b_), 1.46 (m, 1H, H24_b_), 1.28 (d, 1H, *J* = 6.41 Hz, H6′), 1.29 (m, 1H, H15_b_), 1.18 (td, 1H, *J* = 12.51/12.82 Hz, *J* = 3.97/4.27 Hz, H12_b_), 1.12 (m, 1H, H14), 1.08 (m, 1H, H1_b_), 1.02 (s, 3H, H19), 0.97 (d, 3H, *J* = 6.71 Hz, H21), 0.95 (dd, 1H, *J* = 4.88 Hz, H9), 0.79 (br s, 6H, H18, H27); ^13^C{^1^H} NMR (CDCl_3_, 125 MHz): δ 140.7 (C5), 133.2 (C3′), 128.7 (C2′), 121.5 (C6), 109.3 (C22), 93.0 (C1′), 80.8 (C16), 77.5 (C3), 66.9 (C26), 66.2 (C5′), 64.1 (C4′), 62.1 (C17), 56.5 (C14), 50.1 (C9), 41.6 (C20), 40.3 (C13), 39.8 (C12), 39.0 (C4), 37.4 (C1), 36.9 (C10), 32.1 (C7), 31.9 (C15), 31.4 (C23, C25), 30.3 (C8), 29.9 (C2), 28.8 (C24), 20.9 (C11), 19.4 (C19), 17.1 (C27), 16.3 (C18), 16.1 (C6′), 14.5 (C21); MALDI-TOF-MS *m*/*z*: [M + H]^+^ calcd for C_33_H_51_O_5_, 527.4; found, 527.4; HRMS (ESI) *m*/*z*: [M + HCOO]^−^ calcd for C_34_H_51_O_7_, 571.3635; found, 571.3620.

##### 4.1.3.5 Diosgenyl 2,3-Dideoxy-α-d-*glycero*- (**11α**) and -β-d-*glycero*-Hex-2-enopyranoside (**11β**)

Reaction of diosgenin (0.45 g, 1.08 mmol) with **5** (0.20 g, 1.00 mmol) followed by flash chromatography (eluent C) provided first **11α** (0.10 g, 19%, a white amorphous solid); *R*_*f*_ 0.76 (eluent A); ^1^H NMR (CDCl_3_, 500 MHz): δ 6.01 (ddt, 1H, *J* = 10.38 Hz, *J* = 2.44 Hz, *J* = 1.22 Hz, H3′), 5.76 (ddd, 1H, *J* = 10.37 Hz, *J* = 2.44 Hz, *J* = 1.83 Hz, H2′), 5.37 (d, 1H, *J* = 5.50 Hz, H6), 5.06 (br s, 1H, H1′), 4.41 (q, 1H, *J* = 7.32/7.63 Hz, H16), 4.23 (br m, 1H, H4′), 3.80 (ddd, 1H, *J* = 10.99 Hz, *J* = 5.49 Hz, *J* = 0.91 Hz, H5_a_′), 3.73 (dd, 1H, *J* = 10.99 Hz, *J* = 7.93 Hz, H5_b_′), 3.54 (tt, 1H, *J* = 10.99 Hz, *J* = 4.88 Hz, H3), 3.47 (ddd, 1H, *J* = 10.98 Hz, *J* = 4.27 Hz, *J* = 1.83 Hz, H26_a_), 3.38 (t, 1H, *J* = 10.99 Hz, H26_b_), 2.40 (ddd, 1H, *J* = 13.43 Hz, *J* = 5.50 Hz, *J* = 1.52 Hz, H4_a_), 2.33 (br t, 1H, *J* = 13.13 Hz, *J* = 2.44 Hz, H4_b_), 2.00 (m, 1H, H7_a_), 1.98 (ddd, 1H, *J* = 12.21 Hz, *J* = 7.33 Hz, *J* = 5.19 Hz, H15_a_), 1.89 (m, 1H, H2_a_), 1.87 (m, 1H, H20), 1.86 (m, 1H, H1_a_), 1.77 (dd, 1H, *J* = 8.54 Hz, *J* = 7.02 Hz, H17), 1.74 (dt, 1H, *J* = 12.51 Hz, *J* = 3.05 Hz, H12_a_), 1.68 (m, 1H, H23_a_), 1.63 (m, 3H, H8, H24_a_, H25), 1.60 (m, 1H, H23_b_), 1.55 (m, 1H, H7_b_), 1.52 (m, 2H, H2_b_, H11_a_), 1.47 (m, 1H, H11_b_), 1.46 (dt, 1H, *J* = 12.82 Hz, *J* = 4.27 Hz, H24_b_), 1.29 (ddd, 1H, *J* = 13.73 Hz, *J* = 11.90 Hz, *J* = 6.41 Hz, H15_b_), 1.18 (td, 1H, *J* = 12.51 Hz, *J* = 4.58 Hz, H12_b_), 1.11 (ddd, 1H, *J* = 13.73 Hz, *J* = 10.68 Hz, *J* = 5.49 Hz, H14), 1.05 (dd, 1H, *J* = 13.73 Hz, *J* = 3.97 Hz, H1_b_), 1.02 (s, 3H, H19), 0.97 (d, 3H, *J* = 7.02 Hz, H21), 0.94 (dd, 1H, *J* = 11.59 Hz, *J* = 5.18 Hz, H9), 0.79 (s, 3H, H18), 0.79 (d, 3H, *J* = 4.41 Hz, H27); ^13^C{^1^H} NMR (CDCl_3_, 125 MHz): δ 140.8 (C5), 132.8 (C3′), 128.4 (C2′), 121.6 (C6), 109.3 (C22), 92.8 (C1′), 80.8 (C16), 77.8 (C3), 66.9 (C26), 63.7 (C5′), 63.3 (C4′), 62.1 (C17), 56.5 (C14), 50.1 (C9), 41.6 (C20), 40.4 (C4), 40.3 (C13), 39.8 (C12), 37.1 (C1), 36.8 (C10), 32.1 (C7), 31.9 (C15), 31.4 (C23, C25), 30.3 (C8), 28.8 (C24), 28.3 (C2), 20.9 (C11), 19.4 (C19), 17.1 (C27), 16.3 (C18), 14.5 (C21); MALDI-TOF-MS *m*/*z*: [M + H]^+^ calcd for C_32_H_49_O_5_, 513.4; found, 513.4, [M + Na]^+^ calcd for C_32_H_48_O_5_Na, 535.3; found, 535.3, [M + K]^+^ calcd for C_32_H_48_O_5_K, 551.3; found, 551.3; HRMS (ESI) *m*/*z*: [M + HCOO]^−^ calcd for C_33_H_49_O_7_, 557.3478; found, 557.3449. Eluted second was **11β** (0.28 g, 55%, a white amorphous solid); *R*_*f*_ 0.65 (eluent E); ^1^H NMR (CDCl_3_, 500 MHz): δ 6.12 (dd, 1H, *J* = 10.07 Hz, *J* = 5.19 Hz, H3′), 5.87 (dd, 1H, *J* = 10.07 Hz, *J* = 3.05 Hz, H2′), 5.35 (d, 1H, *J* = 5.19 Hz, H6), 5.10 (dd, 1H, *J* = 3.05 Hz, *J* = 0.91 Hz, H1′), 4.41 (q, 1H, *J* = 7.32/7.63 Hz, H16), 4.15 (dd, 1H, *J* = 12.21 Hz, *J* = 2.75 Hz, H5_a_′), 3.82 (br m, 1H, H4′), 3.76 (dt, 1H, *J* = 12.21 Hz, *J* = 1.52 Hz, *J* = 1.22 Hz, H5_b_′), 3.55 (tt, 1H, *J* = 11.29 Hz, *J* = 4.88/4.58 Hz, H3), 3.47 (ddd, 1H, *J* = 10.69 Hz, *J* = 4.27 Hz, *J* = 2.13 Hz, H26_a_), 3.37 (t, 1H, *J* = 10.68/10.98 Hz, H26_b_), 2.36 (ddd, 1H, *J* = 13.13 Hz, *J* = 4.88 Hz, *J* = 2.13 Hz, H4_a_), 2.24 (br t, 1H, H4_b_), 2.00 (m, 1H, H7_a_), 1.98 (ddd, 1H, *J* = 11.91 Hz, *J* = 7.33 Hz, *J* = 5.19 Hz, H15_a_), 1.91 (m, 1H, H2_a_), 1.87 (m, 1H, H20), 1.85 (m, 1H, H1_a_), 1.78 (dd, 1H, *J* = 8.55 Hz, *J* = 7.02 Hz, H17), 1.74 (dt, 1H, *J* = 12.51 Hz, *J* = 3.05/3.97 Hz, H12_a_), 1.68 (m, 1H, H23_a_), 1.64 (m, 1H, H25), 1.63 (m, 1H, H8), 1.62 (m, 1H, H24_a_), 1.60 (m, 1H, H23_b_), 1.55 (m, 1H, H7_b_), 1.59 (m, 1H, H2_b_), 1.52 (m, 1H, H11_a_), 1.48 (m, 1H, H11_b_), 1.45 (dt, 1H, *J* = 12.81 Hz, *J* = 4.58 Hz, H24_b_), 1.29 (ddd, 1H, *J* = 13.73 Hz, *J* = 11.91 Hz, *J* = 6.41 Hz, H15_b_), 1.18 (td, 1H, *J* = 12.51/12.82 Hz, *J* = 4.57 Hz, H12_b_), 1.11 (ddd, 1H, *J* = 13.73 Hz, *J* = 10.68 Hz, *J* = 5.49 Hz, H14), 1.09 (m, 1H, H1_b_), 1.02 (s, 3H, H19), 0.97 (d, 3H, *J* = 7.02 Hz, H21), 0.94 (dd, 1H, *J* = 11.59 Hz, *J* = 5.19 Hz, H9), 0.79 (s, 3H, H18), 0.79 (d, 3H, *J* = 6.10 Hz, H27); ^13^C{^1^H} NMR (CDCl_3_, 125 MHz): δ 140.7 (C5), 129.1 (C3′), 129.0 (C2′), 121.6 (C6), 109.3 (C22), 91.5 (C1′), 80.8 (C16), 77.1 (C3), 66.9 (C26), 64.2 (C5′), 62.1 (C17), 61.6 (C4′), 56.5 (C14), 50.1 (C9), 41.6 (C20), 40.3 (C13), 39.8 (C12), 38.9 (C4), 37.4 (C1), 36.9 (C10), 32.1 (C7), 31.9 (C15), 31.4 (C23, C25), 30.3 (C8), 29.8 (C2), 28.8 (C24), 20.8 (C11), 19.4 (C19), 17.1 (C27), 16.3 (C18), 14.5 (C21); MALDI-TOF-MS *m*/*z*: [M + H]^+^ calcd for C_32_H_49_O_5_, 513.4; found, 513.4, [M + Na]^+^ calcd for C_32_H_48_O_5_Na, 535.3; found, 535.4, [M + K]^+^ calcd for C_32_H_48_O_5_K, 551.3; found, 551.4 (M + K)^+^; HRMS (ESI) *m*/*z*: [M + HCOO]^−^ calcd for C_33_H_49_O_7_, 557.3478; found, 557.3458.

##### 4.1.3.6 Diosgenyl 2,3-Dideoxy-α-l-*glycero*- (**12α**) and -β-l-*glycero*-Hex-2-enopyranoside (**12β**)

Reaction of diosgenin (0.21 g, 0.51 mmol) with **6** (0.10 g, 0.50 mmol) followed by flash chromatography (eluent C) gave first **12α** (0.02 g, 8%, a white amorphous solid); *R*_*f*_ 0.71 (eluent A); ^1^H NMR (CDCl_3_, 500 MHz): δ 6.01 (br d, 1H, *J* = 10.37 Hz, H3′), 5.76 (dt, 1H, *J* = 10.07 Hz, *J* = 1.83 Hz, H2′), 5.35 (d, 1H, *J* = 5.19 Hz, H6), 5.07 (br s, 1H, H1′), 4.41 (q, 1H, *J* = 7.32/7.63 Hz, H16), 4.22 (br m, 1H, H4′), 3.78 (dd, 1H, *J* = 10.99 Hz, *J* = 5.49 Hz, H5_a_′), 3.73 (dd, 1H, *J* = 10.99 Hz, *J* = 7.93 Hz, H5_b_′), 3.55 (tt, 1H, *J* = 11.29 Hz, *J* = 4.58 Hz, H3), 3.47 (ddd, 1H, *J* = 10.98 Hz, *J* = 4.28 Hz, *J* = 1.84 Hz, H26_a_), 3.37 (t, 1H, *J* = 10.68/10.98 Hz, H26_b_), 2.35 (ddd, 1H, *J* = 12.82 Hz, *J* = 5.19 Hz, *J* = 1.83 Hz, H4_a_), 2.25 (br t, 1H, H4_b_), 2.02 (m, 1H, H7_a_), 1.99 (m, 1H, H15_a_), 1.89 (m, 1H, H2_a_), 1.87 (m, 1H, H20), 1.86 (m, 1H, H1_a_), 1.78 (dd, 1H, *J* = 8.55 Hz, *J* = 7.02 Hz, H17), 1.74 (dt, 1H, *J* = 12.51 Hz, *J* = 3.05 Hz, H12_a_), 1.69 (m, 1H, H23_a_), 1.64 (m, 1H, H25), 1.63 (m, 2H, H8, H24_a_), 1.61 (m, 2H, H2_b_, H23_b_), 1.55 (m, 1H, H7_b_), 1.52 (m, 1H, H11_a_), 1.48 (m, 1H, H11_b_), 1.46 (m, 1H, H24_b_), 1.28 (m, 1H, H15_b_), 1.18 (td, 1H, *J* = 12.52 Hz, *J* = 4.58 Hz, H12_b_), 1.11 (ddd, 1H, *J* = 13.43 Hz, *J* = 10.98 Hz, *J* = 5.49 Hz, H14), 1.08 (m, 1H, H1_b_), 1.02 (s, 3H, H19), 0.97 (d, 3H, *J* = 7.02 Hz, H21), 0.94 (dd, 1H, *J* = 11.60 Hz, *J* = 5.19 Hz, H9), 0.79 (s, 3H, H18), 0.79 (d, 3H, *J* = 6.10 Hz, H27); ^13^C{^1^H} NMR (CDCl_3_, 125 MHz): δ 140.7 (C5), 132.8 (C3′), 128.4 (C2′), 121.6 (C6), 109.3 (C22), 92.8 (C1′), 80.8 (C16), 77.7 (C3), 66.9 (C26), 63.7 (C5′), 63.2 (C4′), 62.1 (C17), 56.5 (C14), 50.1 (C9), 41.6 (C20), 40.3 (C13), 39.8 (C12), 39.0 (C4), 37.4 (C1), 36.9 (C10), 32.1 (C7), 31.9 (C15), 31.4 (C23, C25), 30.3 (C8), 29.9 (C2), 28.8 (C24), 20.9 (C11), 19.4 (C19), 17.1 (C27), 16.3 (C18), 14.5 (C21); Eluted second was **12β** (0.08 g, 31%, a white amorphous solid); *R*_*f*_ 0.63 (eluent A); ^1^H NMR (CDCl_3_, 500 MHz): δ 6.12 (dd, 1H, *J* = 10.07 Hz, *J* = 5.19 Hz, H3′), 5.86 (dd, 1H, *J* = 10.07 Hz, *J* = 3.05 Hz, H2′), 5.37 (d, 1H, *J* = 5.19 Hz, H6), 5.09 (d, 1H, *J* = 3.05 Hz, H1′), 4.41 (q, 1H, *J* = 7.32/7.63 Hz, H16), 4.16 (dd, 1H, *J* = 12.21 Hz, *J* = 2.74 Hz, H5_a_′), 3.82 (br m, 1H, H4′), 3.78 (dt, 1H, *J* = 12.21 Hz, *J* = 1.22 Hz, H5_b_′), 3.55 (tt, 1H, *J* = 10.98/11.29 Hz, *J* = 4.88 Hz, H3), 3.47 (ddd, 1H, *J* = 10.68/10.99 Hz, *J* = 3.97/4.28 Hz, *J* = 1.83 Hz, H26_a_), 3.38 (t, 1H, *J* = 10.98 Hz, H26_b_), 2.38 (ddd, 1H, *J* = 13.12 Hz, *J* = 5.19 Hz, *J* = 1.53 Hz, H4_a_), 2.33 (br t, 1H, H4_b_), 2.02 (ddd, 1H, *J* = 10.38 Hz, *J* = 4.88/5.19 Hz, *J* = 2.44 Hz, H7_a_), 1.98 (ddd, 1H, *J* = 12.21 Hz, *J* = 7.33 Hz, *J* = 5.19 Hz, H15_a_), 1.90 (m, 1H, H2_a_), 1.87 (m, 1H, H20), 1.86 (m, 1H, H1_a_), 1.78 (dd, 1H, *J* = 8.55 Hz, *J* = 7.02 Hz, H17), 1.74 (dt, 1H, *J* = 12.21 Hz, *J* = 3.05 Hz, H12_a_), 1.69 (m, 1H, H23_a_), 1.65 (m, 1H, H25), 1.64 (m, 1H, H8), 1.63 (m, 1H, H24_a_), 1.61 (m, 1H, H23_b_), 1.56 (m, 1H, H7_b_), 1.59 (m, 1H, H2_b_), 1.52 (m, 1H, H11_a_), 1.49 (m, 1H, H11_b_), 1.47 (m, 1H, H24_b_), 1.29 (m, 1H, H15_b_), 1.18 (td, 1H, *J* = 12.82 Hz, *J* = 4.58 Hz, H12_b_), 1.11 (ddd, 1H, *J* = 13.73 Hz, *J* = 10.68 Hz, *J* = 5.49 Hz, H14), 1.05 (m, 1H, H1_b_), 1.02 (s, 3H, H19), 0.97 (d, 3H, *J* = 7.02 Hz, H21), 0.94 (dd, 1H, *J* = 11.59 Hz, *J* = 5.19 Hz, H9), 0.79 (s, 3H, H18), 0.79 (d, 3H, *J* = 6.41 Hz, H27); ^13^C{^1^H} NMR (CDCl_3_, 125 MHz): δ 140.8 (C5), 129.1 (C3′), 129.0 (C2′), 121.6 (C6), 109.3 (C22), 91.5 (C1′), 80.8 (C16), 77.4 (C3), 66.9 (C26), 64.2 (C5′), 62.1 (C17), 61.6 (C4′), 56.5 (C14), 50.1 (C9), 41.6 (C20), 40.3 (C4, C13), 39.8 (C12), 37.2 (C1), 36.8 (C10), 32.1 (C7), 31.9 (C15), 31.4 (C23, C25), 30.3 (C8), 28.8 (C24), 28.2 (C2), 20.9 (C11), 19.4 (C19), 17.1 (C27), 16.3 (C18), 14.5 (C21); MALDI-TOF-MS *m*/*z*: [M + H]^+^ calcd for C_32_H_49_O_5_, 513.4; found, 513.2, [M + K]^+^ calcd for C_32_H_48_O_5_K, 551.3; found, 551.2; HRMS (ESI) *m*/*z*: [M + HCOO]^−^ calcd for C_33_H_49_O_7_, 557.3478; found, 557.3436.

#### 4.1.4 2,3-Dideoxy Diosgenyl Glycosides (**13–18**)

General procedure: Respective 2,3-unsaturated diosgenyl glycoside (**7**–**12**) (0.05–0.10 mmol) was dissolved in a 10% solution of hydrazine hydrate in EtOH (10 mL). The mixture was stirred at 50–55 °C under reflux, and 30% water solution of H_2_O_2_ (3.5–4.0 mL) was added in portions over 2 h. The reaction was continued for next 16–22 h and monitored with TLC (eluent A or E). Next, a catalytical amount of active charcoal was added to eliminate the excess of H_2_O_2_ and the mixture was stirred for another 1 h. Then, the solution was filtered through Celite, diluted with CHCl_3_, and washed three times with water. The organic layer was dried with MgSO_4_, filtered, and evaporated to give the product. In some cases, the crude product was purified using flash chromatography (eluent F).

##### 4.1.4.1 Diosgenyl 2,3-Dideoxy-α-d-*erythro*-hexopyranoside (**13α**)

Hydrogenation of **7α** (34 mg, 0.063 mmol) provided 1 **3α** (32 mg, 93%, a white amorphous solid); *R*_*f*_ 0.33 (eluent A); ^1^H NMR (CDCl_3_, 500 MHz): δ 5.35 (d, 1H, *J* = 4.58 Hz, H6), 4.95 (d, 1H, *J* = 3.05 Hz, H1′), 4.41 (q, 1H, *J* = 7.32/7.93 Hz, H16), 3.81 (br, 2H, 2 x H6′), 3.65 (m, 1H, *J* = 9.16 Hz, H5′), 3.62 (td, 1H, *J* = 9.46 Hz, *J* = 4.88 Hz, H4′), 3.47 (m, 1H, H26_a_), 3.46 (m, 1H, H3), 3.37 (t, 1H, *J* = 10.98 Hz, H26_b_), 2.33 (br d, 2H, *J* = 7.63 Hz, H4_a_, H4_b_), 1.99 (m, 1H, H7_a_), 1.99 (ddd, 1H, *J* = 12.51 Hz, *J* = 7.63 Hz, *J* = 4.88 Hz, H15_a_), 1.87 (m, 2H, H3_a_′, H20), 1.86 (m, 1H, H2_a_), 1.85 (m, 1H, H1_a_), 1.77 (dd, 1H, *J* = 8.54 Hz, *J* = 6.72 Hz, H17), 1.76 (m, 1H, H2_a_′), 1.76 (m, 1H, H3_b_′), 1.74 (m, 1H, H12_a_), 1.69 (m, 1H, H23_a_), 1.63 (m, 1H, H8), 1.62 (m, 1H, H24_a_), 1.61 (m, 2H, H23_b_, H25), 1.55 (m, 1H, H7_b_), 1.52 (m, 1H, H11_a_), 1.47 (m, 1H, H11_b_), 1.46 (m, 1H, H24_b_), 1.45 (m, 1H, H2_b_), 1.28 (m, 1H, H15_b_), 1.26 (m, 1H, H2_b_′), 1.18 (td, 1H, *J* = 12.51 Hz, *J* = 4.57 Hz, H12_b_), 1.10 (ddd, 1H, *J* = 13.73 Hz, *J* = 10.38 Hz, *J* = 5.49 Hz, H14), 1.03 (m, 1H, H1_b_), 1.03 (s, 1H, H19), 0.97 (d, 3H, *J* = 7.02 Hz, H21), 0.94 (dd, 1H, *J* = 11.90 Hz, *J* = 5.49 Hz, H9), 0.79 (s, 3H, H18), 0.79 (d, 3H, *J* = 6.10 Hz, H27); ^13^C{^1^H} NMR (CDCl_3_, 125 MHz): δ 140.9 (C5), 121.5 (C6), 109.3 (C22), 93.8 (C1′), 80.8 (C16), 75.8 (C3), 72.8 (C5′), 67.7 (C4′), 66.9 (C26), 63.4 (C6′), 62.1 (C17), 56.5 (C14), 50.0 (C9), 41.6 (C20), 40.3 (C13), 40.1 (C4), 39.8 (C12), 37.1 (C10), 36.9 (C1), 32.1 (C7), 31.9 (C15), 31.4 (C23), 31.4 (C25), 30.3 (C8), 29.8 (C2′), 28.8 (C24), 27.8 (C2), 27.3 (C3′), 20.8 (C11), 19.4 (C19), 17.1 (C27), 16.3 (C18), 14.5 (C21); MALDI-TOF-MS *m*/*z*: [M + H]^+^ calcd for C_33_H_53_O_6_, 545,4; found, 545.5, [M + Na]^+^ calcd for C_33_H_52_O_6_Na, 567.4; found, 567.3; HRMS (ESI) *m*/*z*: [M + HCOOH] calcd for C_34_H_54_O_8_, 590.3819; found, 590.3824.

##### 4.1.4.2 Diosgenyl 2,3-Dideoxy-α-d-*threo*-hexopyranoside (**14α**)

Hydrogenation of **8α** (31 mg, 0.057 mmol) gave **14α** (28 mg, 89%, a white amorphous solid); *R*_*f*_ 0.30 (eluent A); ^1^H NMR (CDCl_3_, 500 MHz): δ 5.33 (d, 1H, *J* = 4.88 Hz, H6), 5.07 (d, 1H, *J* = 2.44 Hz, H1′), 4.41 (q, 1H, *J* = 7.32/7.63 Hz, H16), 3.93 (br, 1H, H4′), 3.88 (t, 1H, *J* = 5.19 Hz, *J* = 3.97 Hz, H5′), 3.87 (m, 1H, H6_a_′), 3.83 (dd, 1H, *J* = 12.81 Hz, *J* = 5.19 Hz, H6_b_′), 3.47 (ddd, 1H, *J* = 10.68 Hz, *J* = 4.58 Hz, *J* = 1.52 Hz, H26_a_), 3.48 (m, 1H, H3), 3.37 (t, 1H, *J* = 10.98 Hz, H26_b_), 2.33 (tq, 1H, *J* = 12.52/12.82 Hz, *J* = 1.83/2.44 Hz, H4_a_), 2.30 (m, 1H, H4_b_) 2.09 (tt, 1H, *J* = 13.13/13.73 Hz, *J* = 3.36/3.97 Hz, H2_a_′), 2.02 (m, 1H, H3_a_′), 1.99 (m, 1H, H7_a_), 1.98 (ddd, 1H, *J* = 12.21 Hz, *J* = 7.32 Hz, *J* = 4.88 Hz, H15_a_), 1.90 (m, 1H, H2_a_), 1.87 (m, 1H, H20), 1.86 (m, 1H, H1_a_), 1.77 (dd, 1H, *J* = 8.54 Hz, *J* = 7.02 Hz, H17), 1.71 (m, 1H, H3_b_′), 1.74 (dt, 1H, *J* = 11.91 Hz, *J* = 3.36 Hz, H12_a_), 1.68 (m, 1H, H23_a_), 1.65 (m, 1H, H25), 1.63 (m, 2H, H8, H24_a_), 1.60 (m, 1H, H23_b_), 1.54 (m, 1H, H7_b_), 1.52 (m, 1H, H2_b_′), 1.51 (m, 1H, H11_a_), 1.48 (m, 1H, H11_b_), 1.46 (m, 1H, H2_b_), 1.45 (dt, 1H, *J* = 12.82 Hz, *J* = 4.58 Hz, H24_b_), 1.29 (ddd, 1H, *J* = 13.74 Hz, *J* = 12.21 Hz, *J* = 6.41 Hz, H15_b_), 1.18 (td, 1H, *J* = 12.82 Hz, *J* = 4.58 Hz, H12_b_), 1.10 (ddd, 1H, *J* = 13.74 Hz, *J* = 10.68 Hz, *J* = 5.49 Hz, H14), 1.04 (m, 1H, H1_b_), 1.02 (s, 1H, H19), 0.97 (d, 3H, *J* = 7.02 Hz, H21), 0.94 (dd, 1H, *J* = 11.60 Hz, *J* = 5.19 Hz, H9), 0.79 (s, 3H, H18), 0.79 (d, 3H, *J* = 6.10 Hz, H27); ^13^C{^1^H} NMR (CDCl_3_, 125 MHz): δ 140.9 (C5), 121.5 (C6), 109.3 (C22), 95.1 (C1′), 80.8 (C16), 76.0 (C3), 69.3 (C5′), 67.0 (C4′), 66.9 (C26), 64.9 (C6′), 62.1 (C17), 56.5 (C14), 50.0 (C9), 41.6 (C20), 40.3 (C13), 40.1 (C4), 39.8 (C12), 37.1 (C10), 36.9 (C1), 32.1 (C7), 31.9 (C15), 31.4 (C23), 31.4 (C25), 30.3 (C8), 28.8 (C24), 27.9 (C2), 25.4 (C3′), 24.0 (C2′), 20.8 (C11), 19.4 (C19), 17.1 (C27), 16.3 (C18), 14.5 (C21); MALDI-TOF-MS *m*/*z*: [M + H]^+^ calcd for C_33_H_53_O_6_, 545.4; found, 545.5, [M + Na]^+^ calcd for C_33_H_52_O_6_Na, 567.4; found, 567.3, [M + K]^+^ calcd for C_33_H_52_O_6_K, 583.3; found, 583.3; HRMS (ESI) *m*/*z*: [M + HCOOH] calcd for C_34_H_54_O_8_, 590.3819; found, 590.3822.

##### 4.1.4.3 Diosgenyl 2,3,6-Trideoxy-α-l-*erythro*-hexopyranoside (**15α**)

Hydrogenation of **9α** (32 mg, 0.061 mmol) followed by flash chromatography led to **15α** (24 mg, 74%, a white amorphous solid); *R*_*f*_ 0.31 (eluent E); ^1^H NMR (CDCl_3_, 500 MHz): δ 5.33 (d, 1H, *J* = 5.19 Hz, H6), 4.90 (br s, 1H, H1′), 4.41 (q, 1H, *J* = 7.32/7.63 Hz, H16), 3.66 (qd, 1H, *J* = 8.85/9.16 Hz, *J* = 6.10/6.41 Hz, H5′), 3.48 (m, 1H, H3), 3.47 (m, 1H, 26_a_), 3.37 (t, 1H, *J* = 10.68/10.99 Hz, H26_b_), 3.26 (td, 1H, *J* = 8.85/9.16 Hz, *J* = 3.96 Hz, H4′), 2.34 (ddd, 1H, *J* = 13.12 Hz, *J* = 4.88 Hz, *J* = 2.14 Hz, H4_a_), 2.25 (tq, 1H, *J* = 12.21/12.36 Hz, *J* = 2.14 Hz, H4_b_), 1.99 (m, 2H, H7_a_, H15_a_), 1.87 (m, 1H, H20), 1.86 (m, 1H, H2_a_), 1.85 (m, 2H, H1_a_, H2_a_′), 1.78 (m, 2H, H3_a_′, H3_b_′), 1.77 (m, 1H, H17), 1.76 (m, 1H, H2_b_′), 1.74 (dt, 1H, *J* = 12.51 Hz, *J* = 3.35 Hz, H12_a_), 1.68 (m, 1H, H23_a_), 1.64 (m, 1H, H25), 1.63 (m, 2H, H8, H24_a_), 1.60 (m, 2H, H2_b_, H23_b_), 1.54 (m, 1H, H7_b_), 1.52 (m, 1H, H11_a_), 1.46 (m, 2H, H11_b_, H24_b_), 1.28 (ddd, 1H, *J* = 13.73 Hz, *J* = 12.21 Hz, *J* = 6.41 Hz, H15_b_), 1.23 (d, 1H, *J* = 6.10 Hz, H6′), 1.18 (td, 1H, *J* = 12.82 Hz, *J* = 4.27 Hz, H12_b_), 1.11 (ddd, 1H, *J* = 13.73 Hz, *J* = 10.68 Hz, *J* = 5.49 Hz, H14), 1.08 (m, 1H, H1_b_), 1.03 (s, 1H, H19), 0.97 (d, 3H, *J* = 7.02 Hz, H21), 0.95 (dd, 1H, *J* = 11.29 Hz, *J* = 5.18 Hz, H9), 0.79 (s, 3H, H18), 0.79 (d, 3H, *J* = 6.10 Hz, H27); ^13^C{^1^H} NMR (CDCl_3_, 125 MHz): δ 140.8 (C5), 121.4 (C6), 109.3 (C22), 94.0 (C1′), 80.8 (C16), 75.6 (C3), 72.3 (C4′), 69.5 (C5′), 66.8 (C26), 62.1 (C17), 56.5 (C14), 50.1 (C9), 41.6 (C20), 40.3 (13), 39.8 (C12), 38.6 (C4), 37.4 (C1), 36.9 (C10), 32.1 (C7), 31.9 (C15), 31.5 (C25), 31.4 (C23), 31.1 (C3′), 30.3 (C8), 29.5 (C2), 28.8 (C24), 27.6 (C2′), 20.8 (C11), 19.4 (C19), 17.9 (C6′), 17.1 (C27), 16.3 (C18), 14.5 (C21); MALDI-TOF-MS *m*/*z*: [M + H]^+^ calcd for C_33_H_53_O_5_, 529.4; found, 529.4, [M + Na]^+^ calcd for C_33_H_52_O_5_Na, 551.4; found, 551.3; HRMS (ESI) *m*/*z*: [M + HCOO]^−^ calcd for C_34_H_53_O_7_, 573.3791; found, 573.3814.

##### 4.1.4.4 Diosgenyl 2,3,6-Trideoxy-α-l-*threo*-hexopyranoside (**16α**)

Hydrogenation of **10α** (51 mg, 0.097 mmol) followed by flash chromatography gave **16α** (38 mg, 74%, a white amorphous solid); *R*_*f*_ 0.33 (eluent E); ^1^H NMR (CDCl_3_, 500 MHz): δ 5.34 (d, 1H, *J* = 3.96 Hz, H6), 4.96 (br s, 1H, H1′), 4.41 (q, 1H, *J* = 7.32 Hz, H16), 4.04 (q, 1H, *J* = 6.41/6.71 Hz, H5′), 3.56 (b, 1H, H4′), 3.47 (m, 2H, H3, 26_a_), 3.37 (t, 1H, *J* = 10.99 Hz, H26_b_), 2.34 (dd, 1H, *J* = 13.12/13.43 Hz, *J* = 3.96/4.27 Hz, H4_a_), 2.19 (bt, 1H, *J* = 11.91/12.20 Hz, H4_b_), 2.02 (m, 1H, H3_a_′), 1.99 (m, 2H, H7_a_, H15_a_), 1.97 (m, 1H, H2_a_′), 1.87 (m, 1H, H20), 1.85 (m, 2H, H1_a_, H2_a_), 1.78 (m, 1H, H17), 1.74 (m, 2H, H3_b_′, H12_a_), 1.67 (m, 1H, H23_a_), 1.65 (m, 1H, H25), 1.63 (m, 1H, H8), 1.62 (m, 1H, H24_a_), 1.61 (m, 1H, H23_b_), 1.54 (m, 1H, H7_b_), 1.52 (m, 1H, H11_a_), 1.51 (m, 1H, H2_b_′), 1.47 (m, 1H, H24_b_), 1.46 (m, 1H, H11_b_), 1.29 (m, 1H, H15_b_), 1.26 (m, 1H, H2_b_), 1.18 (td, 1H, *J* = 12.21/12.51 Hz, *J* = 3.97/4.27 Hz, H12_b_), 1.15 (d, 1H, *J* = 6.41 Hz, H6′), 1.11 (m, 1H, H14), 1.06 (m, 1H, H1_b_), 1.03 (s, 1H, H19), 0.97 (d, 3H, *J* = 7.02 Hz, H21), 0.95 (dd, 1H, *J* = 4.89 Hz, H9), 0.79 (br s, 6H, H18, H27); ^13^C{^1^H} NMR (CDCl_3_, 125 MHz): δ 140.8 (C5), 121.4 (C6), 109.3 (C22), 95.1 (C1′), 80.8 (C16), 75.9 (C3), 67.6 (C4′), 66.9 (C26), 66.1 (C5′), 62.1 (C17), 56.5 (C14), 50.1 (C9), 41.6 (C20), 40.3 (13), 39.8 (C12), 38.7 (C4), 37.4 (C1), 36.9 (C10), 32.1 (C7), 31.9 (C15), 31.5 (C25), 31.4 (C23), 30.3 (C8), 29.5 (C2), 28.8 (C24), 25.9 (C3′), 23.9 (C2′), 20.8 (C11), 19.4 (C19), 17.1 (C6′), 17.1 (C27), 16.3 (C18), 14.5 (C21); MALDI-TOF-MS *m*/*z*: [M + H]^+^ calcd for C_33_H_53_O_5_, 529.4; found, 529.4, [M + Na]^+^ calcd for C_33_H_52_O_5_Na, 551.4; found, 551.3, [M + K]^+^ calcd for C_33_H_52_O_5_K, 567.3; found, 567.3; HRMS (ESI) *m*/*z*: [M + HCOO]^−^ calcd for C_34_H_53_O_7_, 573.3791; found, 573.3801.

##### 4.1.4.5 Diosgenyl 2,3-Dideoxy-α-d-*glycero*-hexopyranoside (**17α**)

Hydrogenation of **11α** (47 mg, 0.092 mmol) provided **17α** (23 mg, 48%, a white amorphous solid); *R*_*f*_ 0.29 (eluent A); ^1^H NMR (CDCl_3_, 500 MHz): δ 5.36 (d, 1H, *J* = 5.18 Hz, H6), 4.74 (t, 1H, *J* = 3.97/2.75 Hz, H1′), 4.41 (q, 1H, *J* = 7.32/7.93 Hz, H16), 3.72 (br, 1H, H4′), 3.67 (dd, 1H, *J* = 10.98 Hz, *J* = 7.02 Hz, H5_a_′), 3.61 (dd, 1H, *J* = 10.99 Hz, *J* = 3.36 Hz, H5_b_′), 3.50 (m, 1H, H3), 3.47 (ddd, 1H, *J* = 11.29 Hz, *J* = 4.27 Hz, *J* = 1.53 Hz, H26_a_), 3.38 (t, 1H, *J* = 10.99 Hz, H26_b_), 2.36 (m, 2H, H4_a_, H4_b_), 2.01 (ddd, 1H, *J* = 12.52 Hz, *J* = 4.88 Hz, *J* = 1.83 Hz, H7_a_), 1.98 (ddd, 1H, *J* = 12.51 Hz, *J* = 7.63 Hz, *J* = 4.88 Hz, H15_a_), 1.88 (m, 1H, H2_a_), 1.87 (m, 1H, H20), 1.85 (m, 1H, H1_a_), 1.82 (m, 3H, H2_a_′, H3_a_′, H3_b_′), 1.77 (dd, 1H, *J* = 8.54 Hz, *J* = 7.02 Hz, H17), 1.74 (m, 1H, H12_a_), 1.71 (m, 1H, H2_b_′), 1.68 (m, 1H, H23_a_), 1.65 (m, 1H, H25), 1.63 (m, 1H, H8), 1.62 (m, 1H, H24_a_), 1.60 (m, 1H, H23_b_), 1.52 (m, 2H, H7_b_, H11_a_), 1.48 (m, 1H, H11_b_), 1.46 (m, 1H, H2_b_), 1.46 (dt, 1H, *J* = 12.51 Hz, *J* = 3.96 Hz, H24_b_), 1.29 (ddd, 1H, *J* = 13.43 Hz, *J* = 12.21 Hz, *J* = 6.41 Hz, H15_b_), 1.18 (td, 1H, *J* = 12.51/12.82 Hz, *J* = 4.58 Hz, H12_b_), 1.10 (ddd, 1H, *J* = 13.73 Hz, *J* = 10.68 Hz, *J* = 5.49 Hz, H14), 1.03 (m, 1H, H1_b_), 1.03 (s, 1H, H19), 0.97 (d, 3H, *J* = 7.02 Hz, H21), 0.94 (dd, 1H, *J* = 11.60 Hz, *J* = 5.19 Hz, H9), 0.79 (s, 3H, H18), 0.79 (d, 3H, *J* = 6.41 Hz, H27); ^13^C{^1^H} NMR (CDCl_3_, 125 MHz): δ 140.9 (C5), 121.4 (C6), 109.3 (C22), 95.6 (C1′), 80.8 (C16), 76.2 (C3), 66.9 (C26), 66.6 (C5′), 65.5 (C4′), 62.1 (C17), 56.5 (C14), 50.1 (C9), 41.6 (C20), 40.3 (C13), 40.1 (C4), 39.8 (C12), 37.1 (C10), 36.9 (C1), 32.1 (C7), 31.9 (C15), 31.4 (C23), 31.4 (C25), 30.3 (C8), 28.8 (C24), 28.4 (C2′), 28.3 (C3′), 27.9 (C2), 20.9 (C11), 19.4 (C19), 17.1 (C27), 16.3 (C18), 14.5 (C21); MALDI-TOF-MS *m*/*z*: [M + H]^+^ calcd for C_32_H_51_O_5_, 515.4; found, 515.5, [M + Na]^+^ calcd for C_32_H_50_O_5_Na, 537.4; found, 537.4, [M + K]^+^ calcd for C_32_H_50_O_5_K, 553.3; found, 553.4; HRMS (ESI) *m*/*z*: [M + HCOO]^−^ calcd for C_33_H_51_O_7_, 559.3635; found, 559.3645.

##### 4.1.4.6 Diosgenyl 2,3-Dideoxy-β-d-*glycero*-hexopyranoside (**17β**)

Hydrogenation of **11β** (49 mg, 0.096 mmol) provided **17β** (28 mg, 57%, a white amorphous solid); *R*_*f*_ 0.24 (eluent A); ^1^H NMR (CDCl_3_, 500 MHz): δ 5.34 (d, 1H, *J* = 4.88 Hz, H6), 4.84 (t, 1H, *J* = 3.05/2.74 Hz, H1′), 4.41 (q, 1H, *J* = 7.33/7.63 Hz, H16), 4.00 (dd, 1H, *J* = 11.59 Hz, *J* = 2.14 Hz, H5_a_′), 3.78 (br, 1H, H4′), 3.46 (m, 1H, H26_a_), 3.45 (m, 1H, H3), 3.40 (ddd, 1H, *J* = 11.90 Hz, *J* = 3.97 Hz, *J* = 1.22 Hz, H5_b_′), 3.37 (t, 1H, *J* = 10.68 Hz, H26_b_), 2.35 (ddd, 1H, *J* = 13.12 Hz, *J* = 4.88 Hz, *J* = 2.10 Hz, H4_a_), 2.20 (br t, 1H, H4_b_), 2.06 (tt, 1H, 1H, *J* = 11.59 Hz, *J* = 3.36 Hz, H3_a_′), 1.99 (m, 1H, H2_a_′), 1.99 (m, 2H, H7_a_, H15_a_), 1.88 (m, 1H, H2_a_), 1.87 (m, 1H, H20), 1.85 (m, 1H, H1_a_), 1.77 (dd, 1H, *J* = 8.54 Hz, *J* = 7.02 Hz, H17), 1.74 (m, 1H, H12_a_), 1.69 (m, 1H, H23_a_), 1.64 (m, 1H, H25), 1.63 (m, 1H, H8), 1.62 (m, 1H, 24_a_), 1.60 (m, 1H, H23_b_), 1.59 (m, 1H, H3_b_′), 1.54 (m, 2H, H2_b_′, H7_b_), 1.53 (m, 1H, H11_a_), 1.48 (m, 1H, H11_b_), 1.27 (m, 1H, H2_b_), 1.45 (dt, 1H, *J* = 12.82 Hz, *J* = 4.88 Hz, H24_b_), 1.29 (ddd, 1H, *J* = 13.73 Hz, *J* = 12.21 Hz, *J* = 6.41 Hz, H15_b_), 1.18 (td, 1H, *J* = 12.52 Hz, *J* = 4.58 Hz, H12_b_), 1.10 (ddd, 1H, *J* = 13.73 Hz, *J* = 10.99 Hz, *J* = 5.49 Hz, H14), 1.08 (m, 1H, H1_b_), 1.03 (s, 1H, H19), 0.97 (d, 3H, *J* = 7.02 Hz, H21), 0.95 (dd, 1H, *J* = 11.59 Hz, *J* = 5.18 Hz, H9), 0.79 (s, 3H, H18), 0.79 (d, 3H, *J* = 4.27 Hz, H27); ^13^C{^1^H} NMR (CDCl_3_, 125 MHz): δ 140.7 (C5), 121.4 (C6), 109.3 (C22), 95.8 (C1′), 80.8 (C16), 76.1 (C3), 66.9 (C26), 65.8 (C5′), 65.2 (C4′), 62.1 (C17), 56.5 (C14), 50.1 (C9), 41.6 (C20), 40.3 (C13), 39.8 (C12), 38.7 (C4), 37.4 (C1), 36.9 (C10), 32.1 (C7), 31.9 (C15), 31.5 (C25), 31.4 (C23), 30.3 (C8), 28.8 (C24), 29.5 (C2), 26.5 (C3′), 26.3 (C2′), 20.8 (C11), 19.4 (C19), 17.1 (C27), 16.3 (C18), 14.5 (C21); MALDI-TOF-MS *m*/*z*: [M + H]^+^ calcd for C_32_H_51_O_5_, 515.4; found, 515.4, [M + Na]^+^ calcd for C_32_H_50_O_5_Na, 537.4; found, 537.3; HRMS (ESI) *m*/*z*: [M + HCOO]^−^ calcd for C_33_H_51_O_7_, 559.3635; found, 559.3624.

##### 4.1.4.7 Diosgenyl 2,3-Dideoxy-β-l-*glycero*-hexopyranoside (**18β**)

Hydrogenation of **12β** (31 mg, 0.061 mmol) gave **18β** (24 mg, 74%, a white amorphous solid); *R*_*f*_ 0.31 (eluent E); ^1^H NMR (CDCl_3_, 500 MHz): δ 5.35 (d, 1H, *J* = 4.27 Hz, H6), 4.84 (t, 1H, *J* = 3.36/2.97 Hz, H1′), 4.41 (q, 1H, *J* = 7.33/7.63 Hz, H16), 4.01 (dd, 1H, *J* = 11.60 Hz, *J* = 2.44 Hz, H5_a_′), 3.79 (br, 1H, H4′), 3.50 (m, 1H, H3), 3.47 (br dd, 1H, *J* = 10.99 Hz, *J* = 4.27 Hz, H26_a_), 3.41 (ddd, 1H, *J* = 11.90 Hz, *J* = 3.97 Hz, *J* = 1.22 Hz, H5_b_′), 3.37 (t, 1H, *J* = 10.68/10.99 Hz, H26_b_), 2.34 (br d, 2H, *J* = 7.63 Hz, H4_a_, H4_b_), 2.06 (tt, 1H, 1H, *J* = 11.29 Hz, *J* = 3.36/3.66 Hz, H3_a_′), 2.01 (m, 1H, H7_a_), 2.00 (m, 1H, H2_a_′), 1.99 (m, 1H, H15_a_), 1.89 (m, 1H, H2_a_), 1.87 (m, 1H, H20), 1.85 (m, 1H, H1_a_), 1.77 (dd, 1H, *J* = 8.54 Hz, *J* = 7.02 Hz, H17), 1.74 (m, 1H, H12_a_), 1.68 (m, 1H, H23_a_), 1.65 (m, 1H, H25), 1.63 (m, 2H, H8, 24_a_), 1.60 (m, 1H, H23_b_), 1.60 (m, 1H, H3_b_′), 1.54 (m, 2H, H2_b_′, H7_b_), 1.51 (m, 1H, H11_a_), 1.48 (m, 1H, H11_b_), 1.46 (m, 1H, H2_b_), 1.45 (dt, 1H, *J* = 13.43 Hz, *J* = 4.27/4.58 Hz, H24_b_), 1.29 (ddd, 1H, *J* = 13.42 Hz, *J* = 11.91 Hz, *J* = 6.41 Hz, H15_b_), 1.18 (td, 1H, *J* = 12.51/12.82 Hz, *J* = 4.58 Hz, H12_b_), 1.10 (ddd, 1H, *J* = 13.43 Hz, *J* = 10.68 Hz, *J* = 5.49 Hz, H14), 1.04 (m, 1H, H1_b_), 1.03 (s, 1H, H19), 0.97 (d, 3H, *J* = 7.02 Hz, H21), 0.94 (dd, 1H, *J* = 11.60 Hz, *J* = 5.19 Hz, H9), 0.79 (s, 3H, H18), 0.79 (d, 3H, *J* = 6.10 Hz, H27); ^13^C{^1^H} NMR (CDCl_3_, 125 MHz): δ 140.9 (C5), 121.4 (C6), 109.3 (C22), 95.6 (C1′), 80.8 (C16), 76.1 (C3), 66.9 (C26), 65.7 (C5′), 65.1 (C4′), 62.1 (C17), 56.5 (C14), 50.1 (C9), 41.6 (C20), 40.3 (C13), 40.1 (C4), 39.8 (C12), 37.1 (C1), 36.9 (C10), 32.1 (C7), 31.9 (C15), 31.4 (C25), 31.4 (C23), 30.3 (C8), 28.8 (C24), 27.9 (C2), 26.4 (C3′), 26.2 (C2′), 20.9 (C11), 19.4 (C19), 17.1 (C27), 16.3 (C18), 14.5 (C21); MALDI-TOF-MS *m*/*z*: [M + H]^+^ calcd for C_32_H_51_O_5_, 515.4; found, 515.4; HRMS (ESI) *m*/*z*: [M + HCOO]^−^ calcd for C_33_H_51_O_7_, 559.3635; found, 559.3624.

### 4.2 Cytotoxicity Assay

An MTT assay was performed to specify the cytotoxicity of the studied compounds against breast cancer cells (MCF-7 line, ATCC), prostate cancer cells (PC3 line, ATCC) and human keratinocytes (HaCaT line, CLS). The MCF-7 cell line was cultured in an RPMI 1640 medium (Gibco, Thermo Fisher Scientific), the PC3 cell line in a F-12K medium (Gibco, Thermo Fisher Scientific), and the HaCaT cell line in a high-glucose DMEM (Gibco, Thermo Fisher Scientific). Each medium was supplemented with 10% fetal bovine serum (Gibco, Thermo Fisher Scientific) and antibiotics (penicillin and streptomycin, Gibco, Thermo Fisher Scientific). Cells were seeded at a density of 4000 per well into 96-well plates and incubated at 37 °C in a 5% CO_2_ atmosphere. The cells were treated with studied compounds in a concentration range of 0.1–100 μg/mL or with a vehicle (1% DMSO) as a control and subsequently incubated for 48 h. Thereafter, the MTT salt solution (3-(4,5-dimethylthiazol-2-yl)-2,5-diphenyltetrazolium bromide, VWR) was added at a concentration of 4 mg/mL (25 μL per well), and the obtained product (formazan) after 3 h of incubation was dissolved in DMSO. An EnSpire microplate reader (PerkinElmer) was used to measure absorbance at 570 nm (the reference wavelength was 660 nm). The viability of the control cells was taken as 100%. Three independent experiments were conducted in triplicate. Statistical analysis of the results and estimation of IC_50_ values were performed using GraphPad Prism 7 software.

### 4.3 Hemolysis Assay

The hemolytic activity was determined on erythrocytes from defibrinated sheep blood (Graso, Poland). The red blood cells were suspended in phosphoric buffer (PBS; AppliChem, Germany) to a final concentration of 4% and added to a polystyrene 96-well plate and exposed to tested compounds (concentration range of 256–0.25 mg/L) for 1 h at 37 °C. The compounds were suspended in DMSO and serially diluted in PBS (the final concentration of DMSO in samples did not exceed 5%; it was confirmed that such concentration of DMSO did not cause hemolysis). After incubation, the plates were centrifuged (800*g*, 10 min, 4 °C) and absorbance of supernatant was measured with a microplate spectrophotometer under a wavelength of 590 nm (Thermo Scientific Multiskan GO, Thermofisher). Sheep erythrocytes exposed to 1% Triton X-100 served as a positive control (100% hemolysis), while the suspensions were in PBS.

### 4.4 Determination of Antibacterial Activity

MIC was determined with the dilution method in Mueller–Hinton II broth (MHB II) on 96-well polystyrene plates. The reference strains (Polish Academy of Sciences, Wroclaw, Poland) of Gram-positive bacteria (*Enterococcus faecium* ATCC 29,212, *S. aureus* ATCC 6538, *S. aureus* ATCC 43,300, *S. aureus* ATCC 12,598, *S. aureus* ATCC 25,923, *S. aureus*, ATCC 33,591, *Staphylococcus epidermidis* ATCC 14,990) and Gram-negative bacteria (*Acinetobacter baumanii* ATCC 23,506, *Escherichia coli* ATCC 25,922, *Klebsiella pneumoniae* ATCC 700,603, *Pseudomonas aeruginosa* ATCC 9027) were suspended in MHB II medium to initial inoculum of 10^5^ cfu/mL. The microbes were exposed to graded concentrations of tested compounds (range 0.5–256 mg/L) for 18 h at 37 °C under aerobic conditions. The compounds were dissolved in DMSO and then diluted with PBS. The final concentration of DMSO in a sample with microbes did not exceed 5% (it was confirmed that this amount of DMSO did not affect the microbial growth). MIC was taken as the lowest concentration at which no microbial growth was observed. The experiments were performed in triplicate.

### 4.5 Methodology of DFT Calculations

All the calculated structures were built in the MOLDEN program.^67^ The structures were optimized using M06-2X functional^68^ and Pople’s 6-311+G** basis set. The M062X function is recommended to solve a wide variety of chemical problems, including the study of conformational equilibria.^69^ It was previously used by us in conformational studies of glycals, giving high agreement with the experiment.^70^ The optimizations were considered satisfactory if the standard criteria were fulfilled, that is, the energy difference between the optimization cycles was lower than 1 × 10^–6^ hartree and a gradient of <1 × 10^–4^ au was achieved. Standard convergence criteria were employed for all calculations. The Hessian calculations were performed for any geometry optimized to confirm that the stationary points corresponding to the minimum have been obtained with no negative eigenvalues. The Hessian calculations were also used to estimate zero-point vibrational effects, molecular entropies, and thermal energy contributions according to statistical thermodynamics formulas. The relative energy of the *i*th conformer is the sum of electronic and zero-point energy, referring to the energy of the most stable conformer. In turn, the relative Gibbs free energy of the *i*th conformer is the sum of its electronic energy and thermal correction to Gibbs free energy, referred to as the Gibbs free energy of the most stable conformer. All calculations were performed in the gas phase at standard temperature and pressure conditions (298.15 K and 1 atm.), using the GAUSSIAN 09 package.^71^

## Data Availability

The data underlying this study are available in the published article and its Supporting Information.

## Supporting Information Available

NMR spectra of **7α–18β**; viability of PC3, MCF7, and HaCaT cells after treatment with **7α–18β**; IC_50_ values of **7α–18β** toward PC3, MCF7, and HaCaT cells; MIC of **7α–18β** determined on G(+) bacteria; and Cartesian coordinates of optimized structures 1′**–5′** and **1″–5″** (PDF)
