# Supplementary material for: Insight into the Course of the Ferrier Rearrangement Used to Obtain Untypical Diosgenyl Saponins
Source: J Org Chem. 2024 Oct 5;89(20):15026–40. doi: 10.1021/acs.joc.4c01756 (PMC11494662; doi:10.1021/acs.joc.4c01756)
Supplement: Supplementary file 1 — jo4c01756_si_001.pdf [file jo4c01756_si_001.pdf]

Supporting Information for

Insight into the course of the Ferrier rearrangement used to obtain untypical diosgenyl saponins

Grzegorz Detlaff[1], Magdalena Zdrowowicz,[1] Małgorzata Paduszyńska[2], Magdalena Datta,[1] Daria Grzywacz[1], Wojciech Kamysz[2], Janusz Rak,[1] Andrzej Nowacki,[1] Henryk Myszka,[1] Beata Liberek[1*]

[1]Faculty of Chemistry, University of Gdańsk, Wita Stwosza 63, 80-308 Gdańsk, Poland

[2]Faculty of Pharmacy, Medical University of Gdańsk, Hallera 107, 80-416 Gdańsk, Poland

Email: Beata Liberek* - beata.liberek@ug.edu.pl

* Corresponding author

Contents:

**Figure S1**	NMR spectra of **7α** …………………………………………………	S2-S3
**Figure S2**	NMR spectra of **8α** …………………………………………………	S4-S5
**Figure S3**	NMR spectra of **9α** …………………………………………………	S6-S7
**Figure S4**	NMR spectra of **10α** ………………………………………………	S8-S9
**Figure S5**	NMR spectra of **11α** ………………………………………………	S10-S11
**Figure S6**	NMR spectra of **11β** ………………………………………………	S12-S13
**Figure S7**	NMR spectra of **12α** ………………………………………………	S14-S15
**Figure S8**	NMR spectra of **12β** ………………………………………………	S16-S17
**Figure S9**	NMR spectra of **13α** ………………………………………………	S18-S19
**Figure S10**	NMR spectra of **14α** ………………………………………………	S20-S21
**Figure S11**	NMR spectra of **15α** ………………………………………………	S22-S23
**Figure S12**	NMR spectra of **16α** ………………………………………………	S24-S25
**Figure S13**	NMR spectra of **17α** ………………………………………………	S26-S27
**Figure S14**	NMR spectra of **17β** ………………………………………………	S28-S29
**Figure S15**	NMR spectra of **18β** ………………………………………………	S30-S31
**Figure S16**	The viability of PC3, MCF7 and HaCaT cells after 48 h treatment with **7α-18β** ………………………………	S32-S34
**Table S1**	IC_{50} values of **7α-18β** towards cancer (PC3 and MCF7) and normal (HaCaT) cells together with selectivity indexes (SI) ……..	S35
**Table S2**	Minimum inhibitory concentrations of **7α-18β** determined on G(+) bacteria ………………………………………………	S35
**Table S3**	Total energies, Gibbs free energies, the lowest frquencies and Cartesian coordinates of optimized structures **1'-5'** and **1''-5''** ……	S36-S39

A

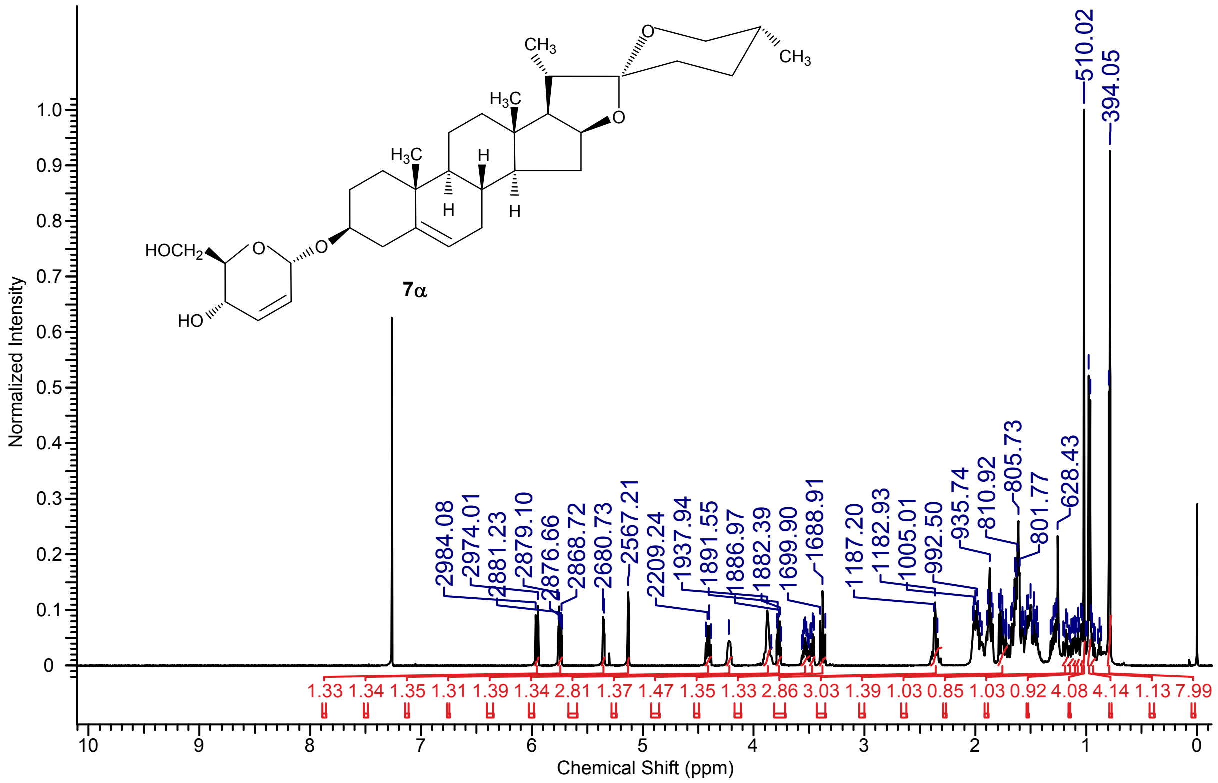

7α
Normalized Intensity
Chemical Shift (ppm)

B

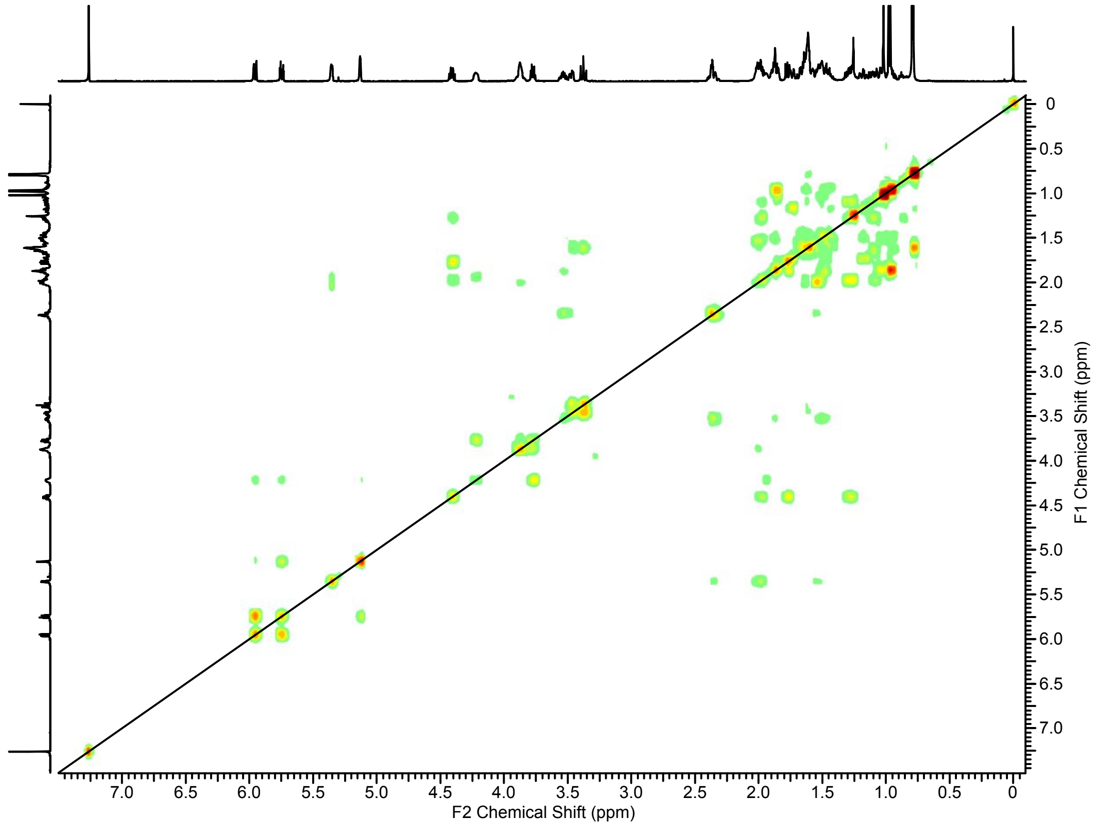

F2 Chemical Shift (ppm)
F1 Chemical Shift (ppm)

C

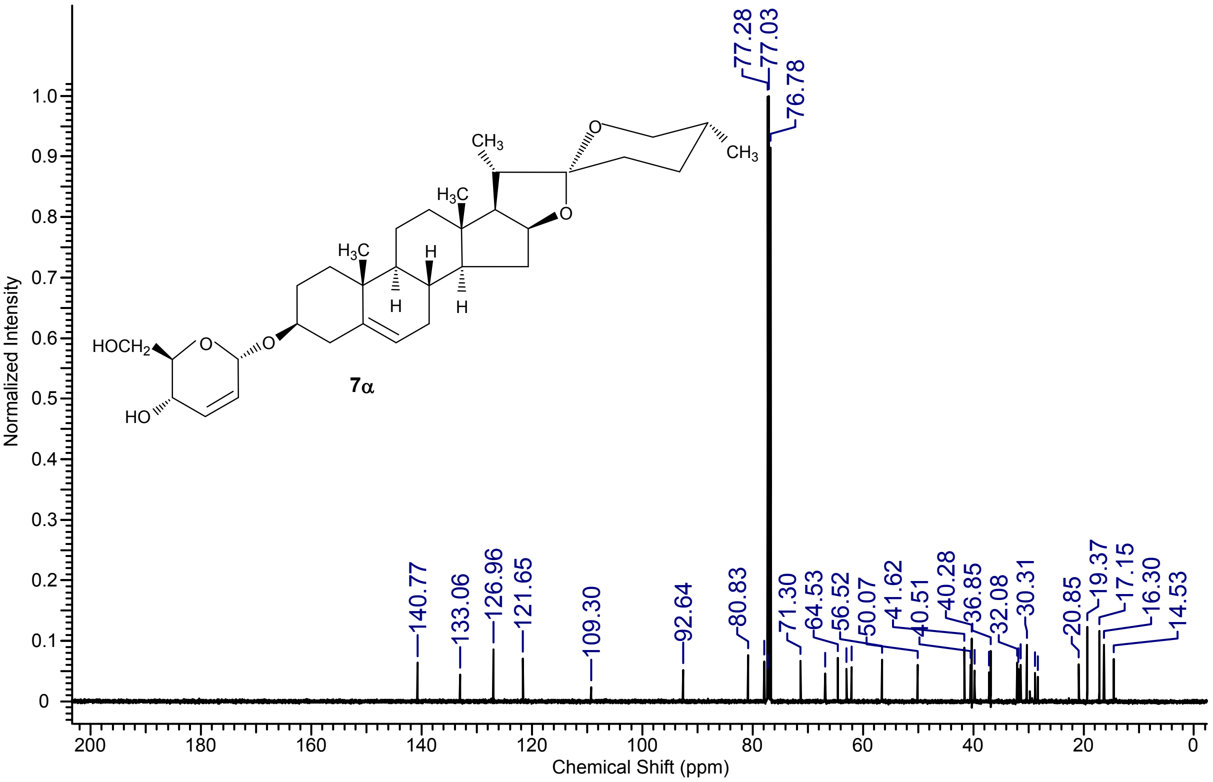

D

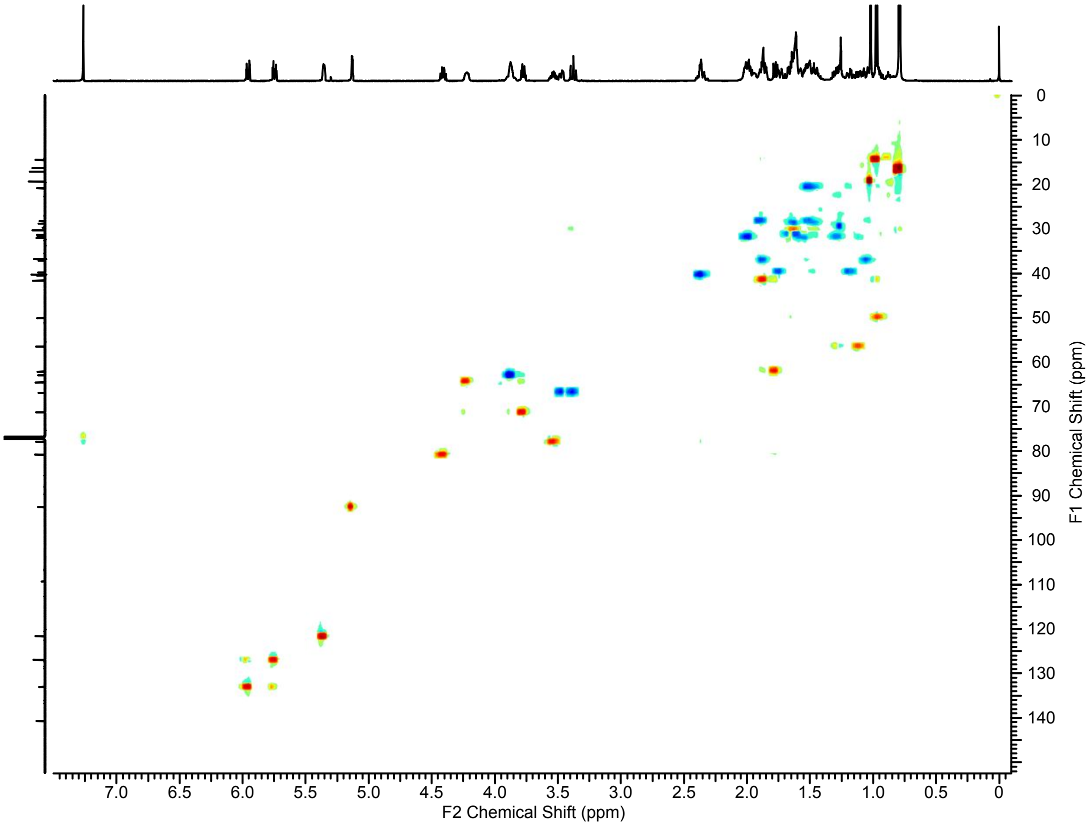

Figure S1. NMR (Chloroform-*d*) spectra of **7α**: ^{1}H NMR (500 MHz) (**A**), gCOSY (**B**), ^{13}C{^{1}H} (125 MHz) (**C**), gHSQC (**D**).

A

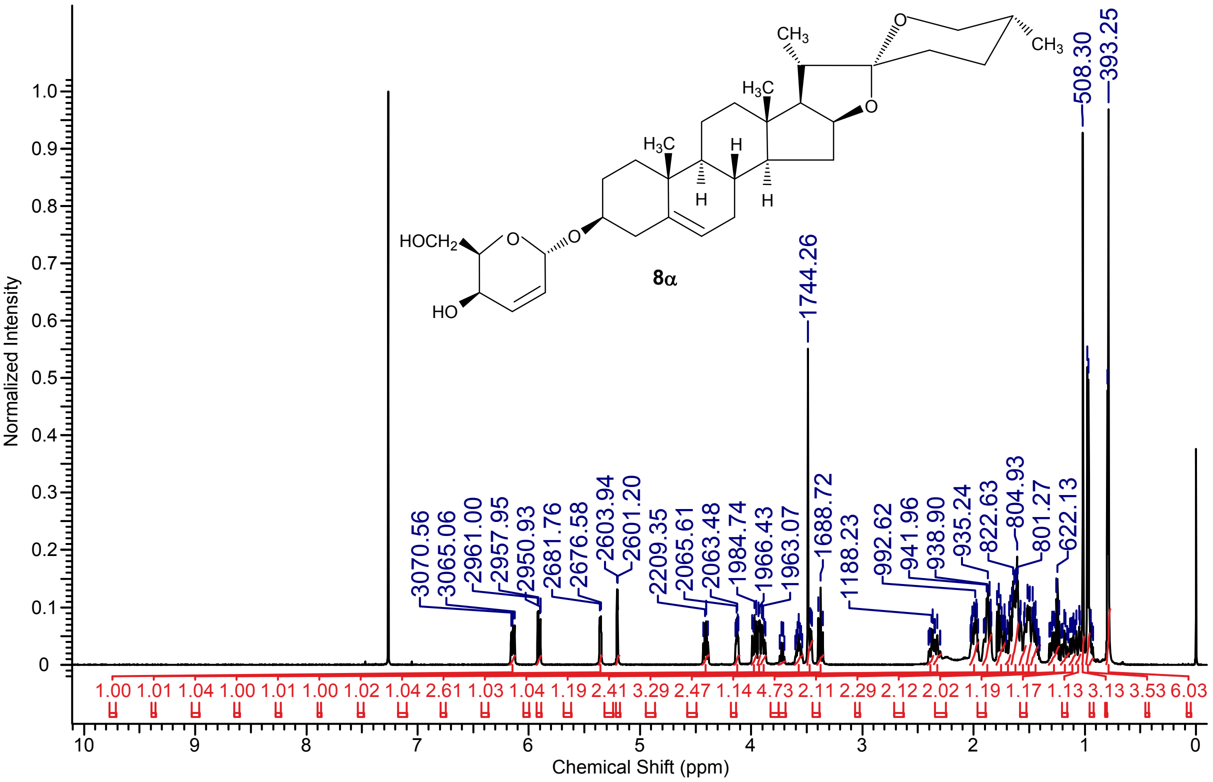

8α
HOCH2
HO
CH3
H3C
Normalized Intensity
Chemical Shift (ppm)

B

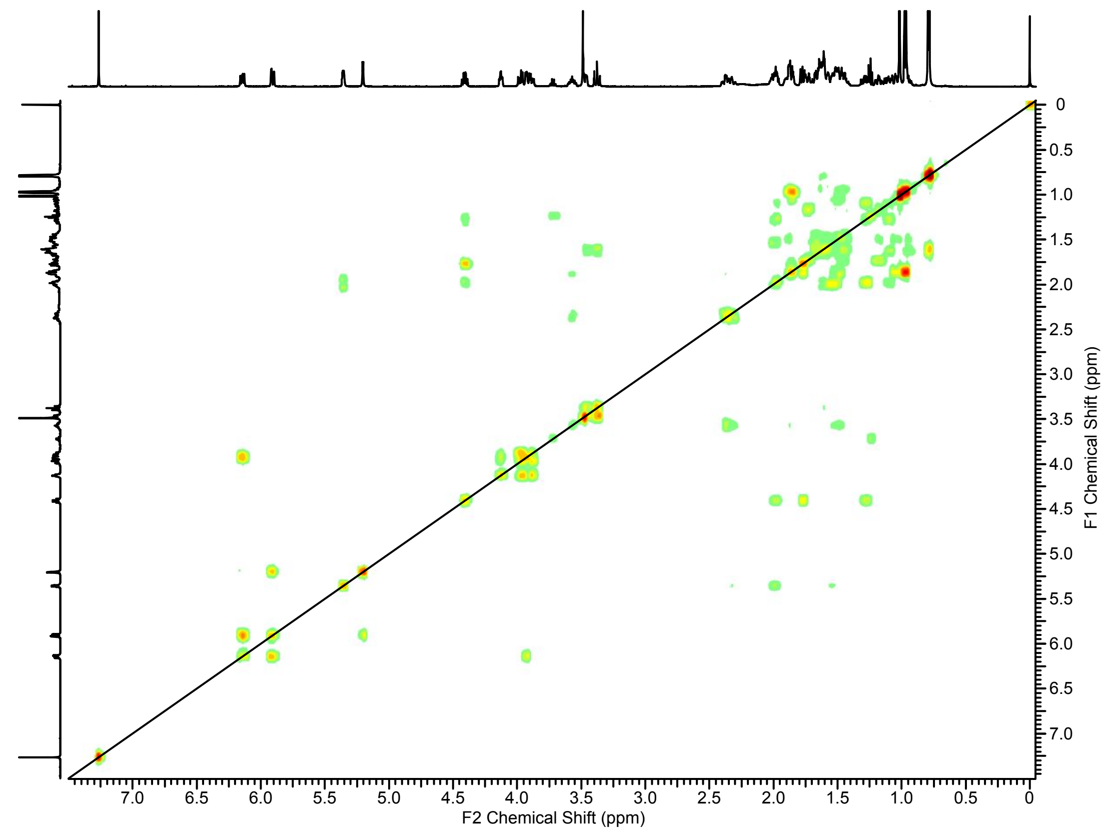

F2 Chemical Shift (ppm)
F1 Chemical Shift (ppm)

C

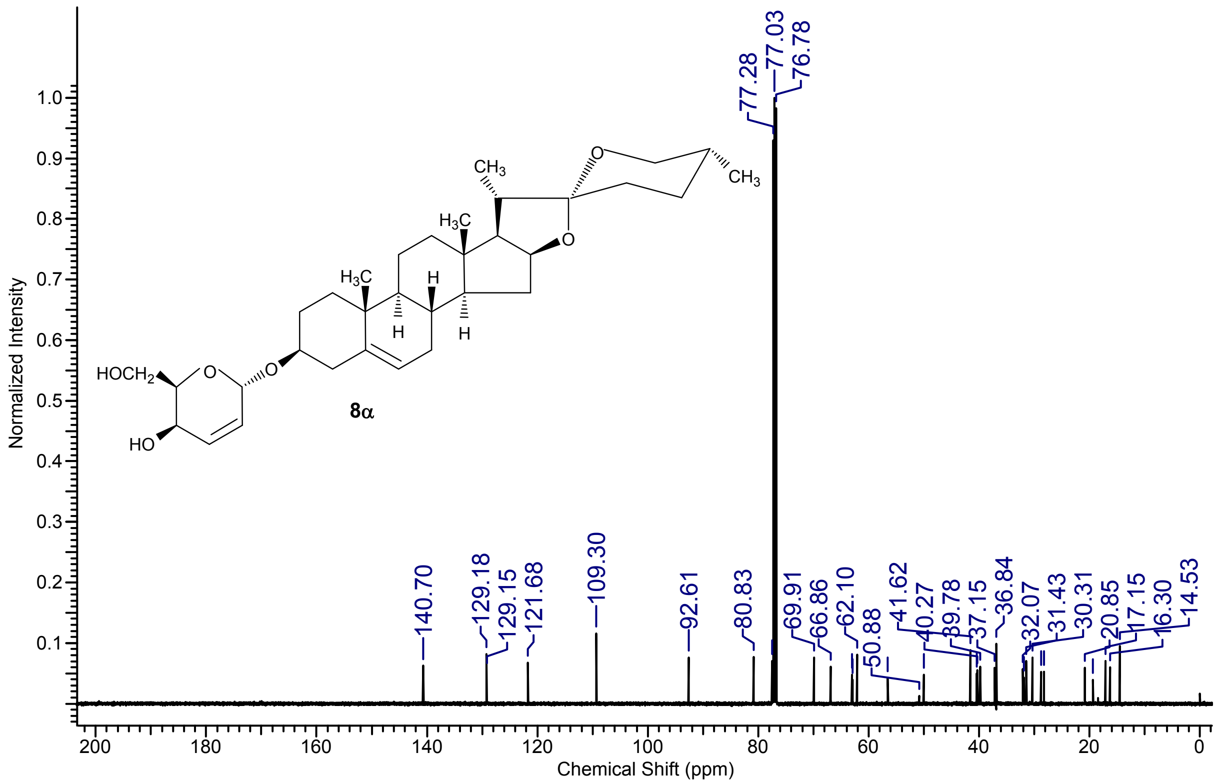

D

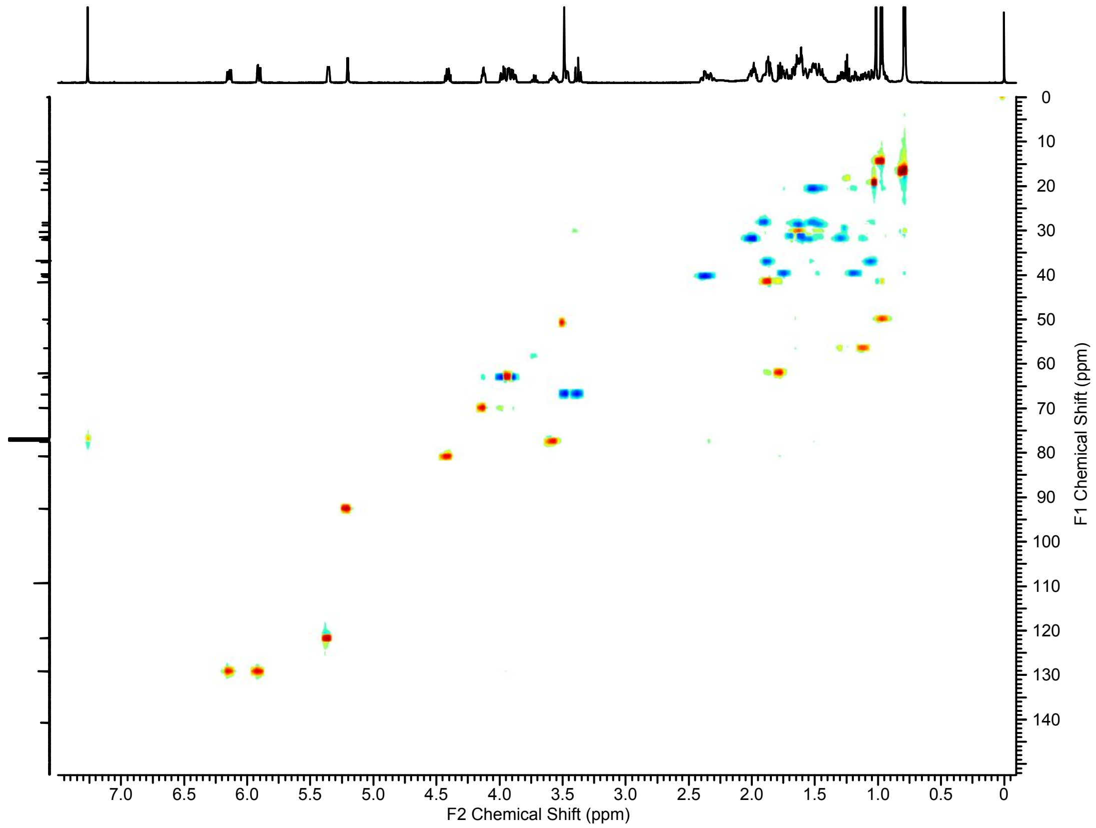

Figure S2. NMR (Chloroform-*d*) spectra of **8α**: ^{1}H NMR (500 MHz) (**A**), gCOSY (**B**), ^{13}C{^{1}H} (125 MHz) (**C**), gHSQC (**D**).

A

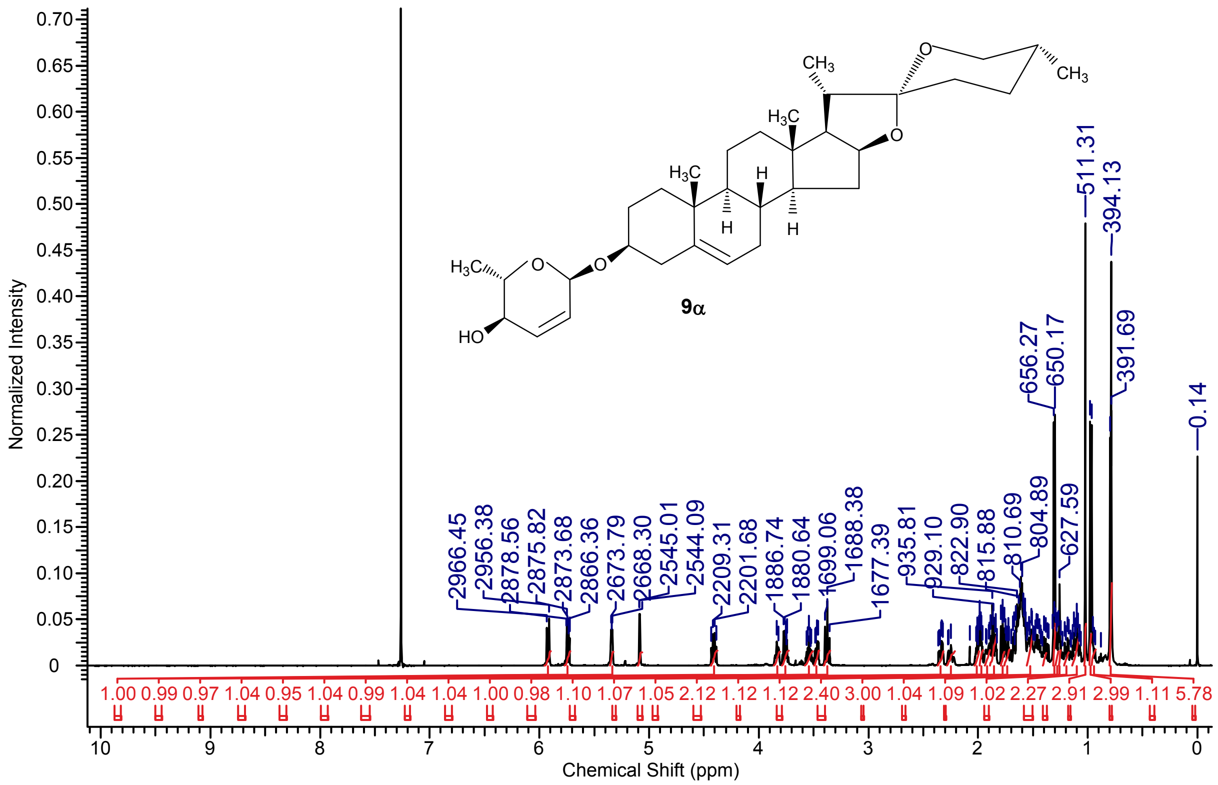
Normalized Intensity
Chemical Shift (ppm)
9α

B

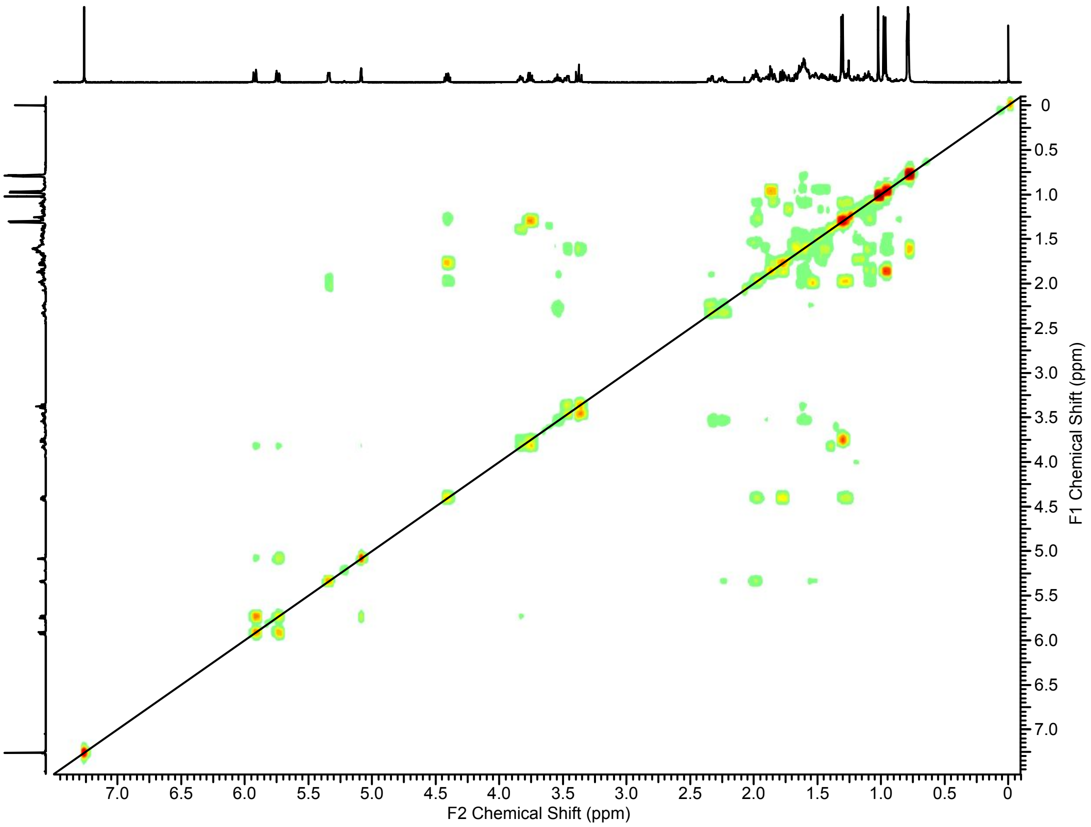
F2 Chemical Shift (ppm)
F1 Chemical Shift (ppm)

C

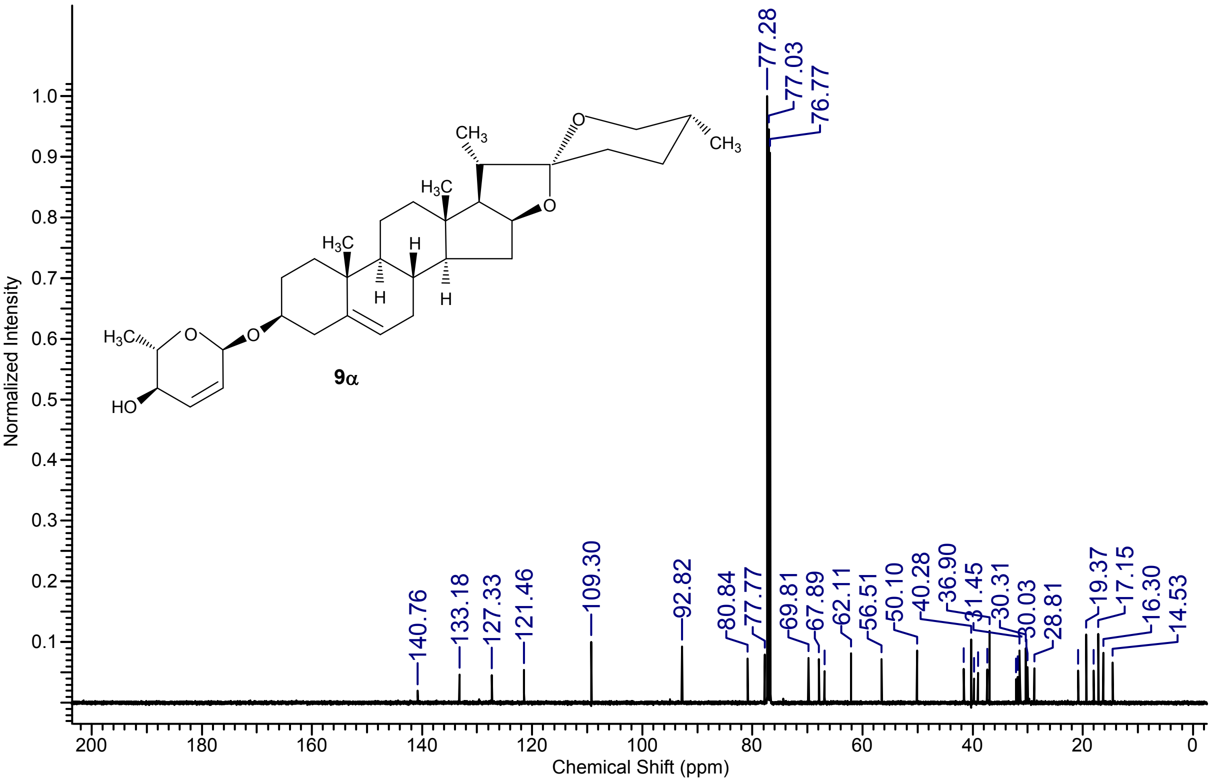

D

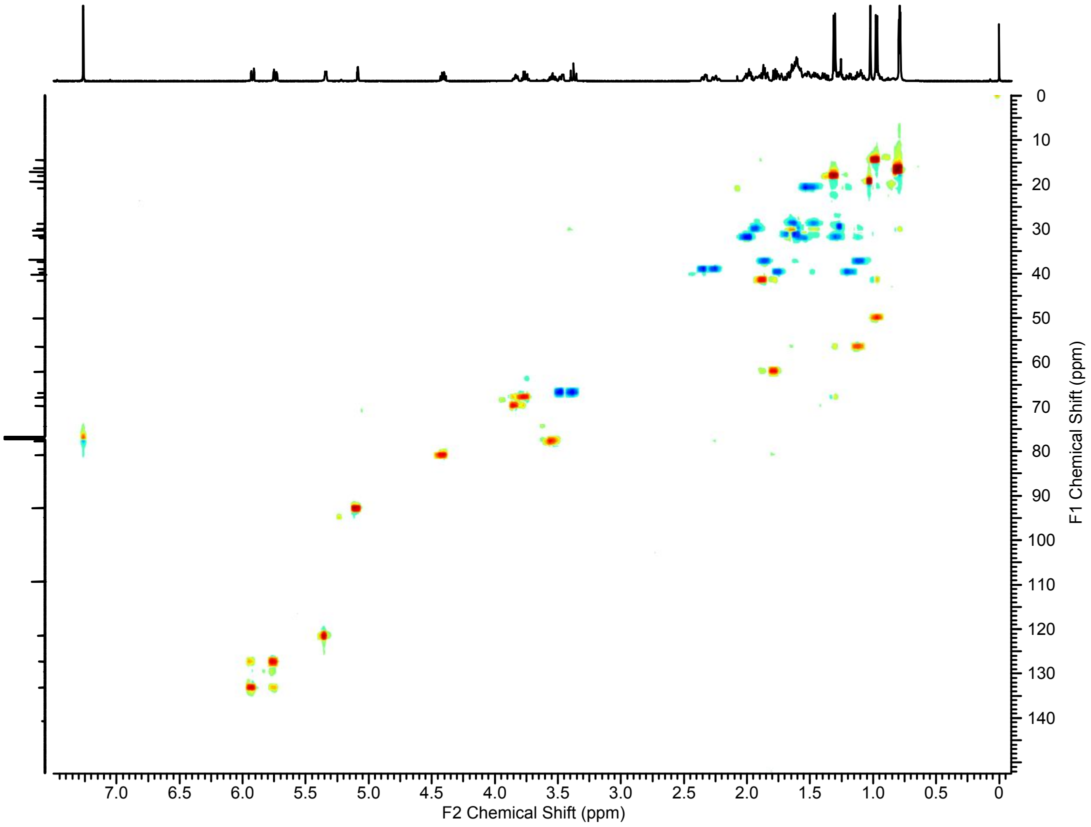

Figure S3. NMR (Chloroform-*d*) spectra of **9α**: ^{1}H NMR (500 MHz) (**A**), gCOSY (**B**), ^{13}C{^{1}H} (125 MHz) (**C**), gHSQC (**D**).

A

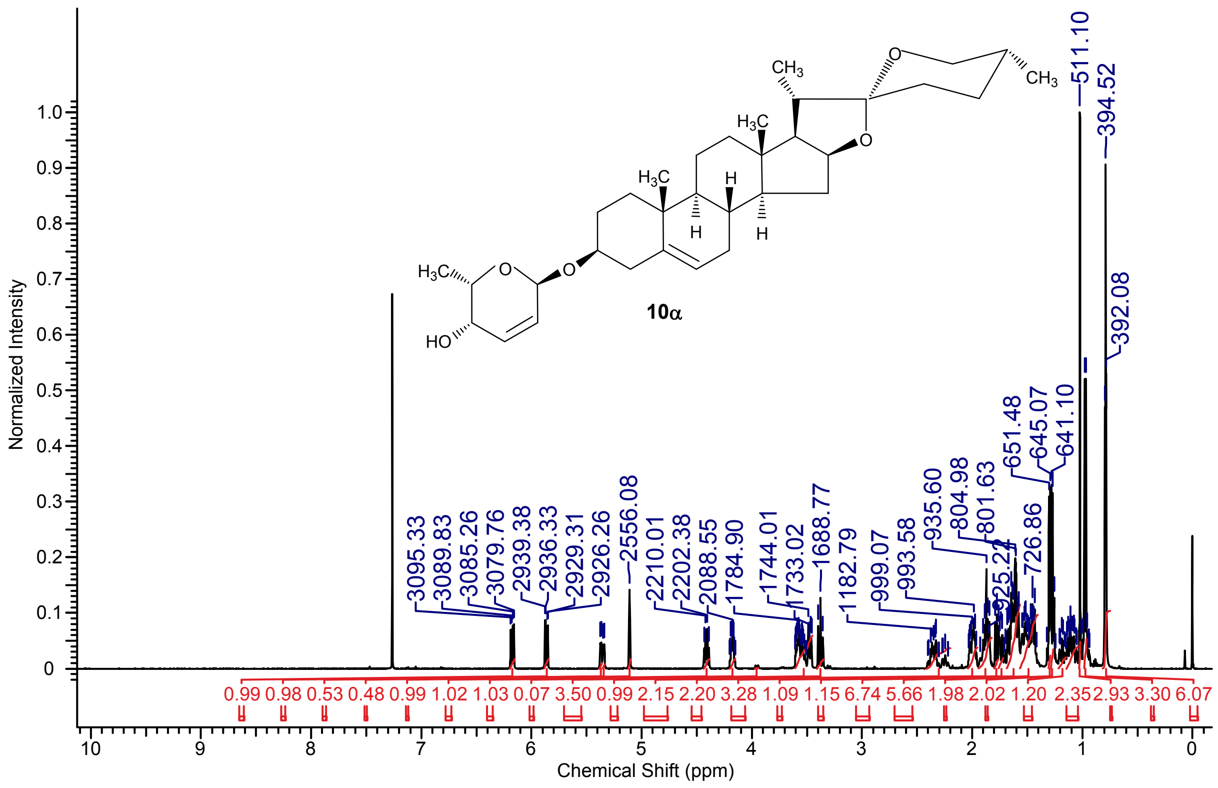

B

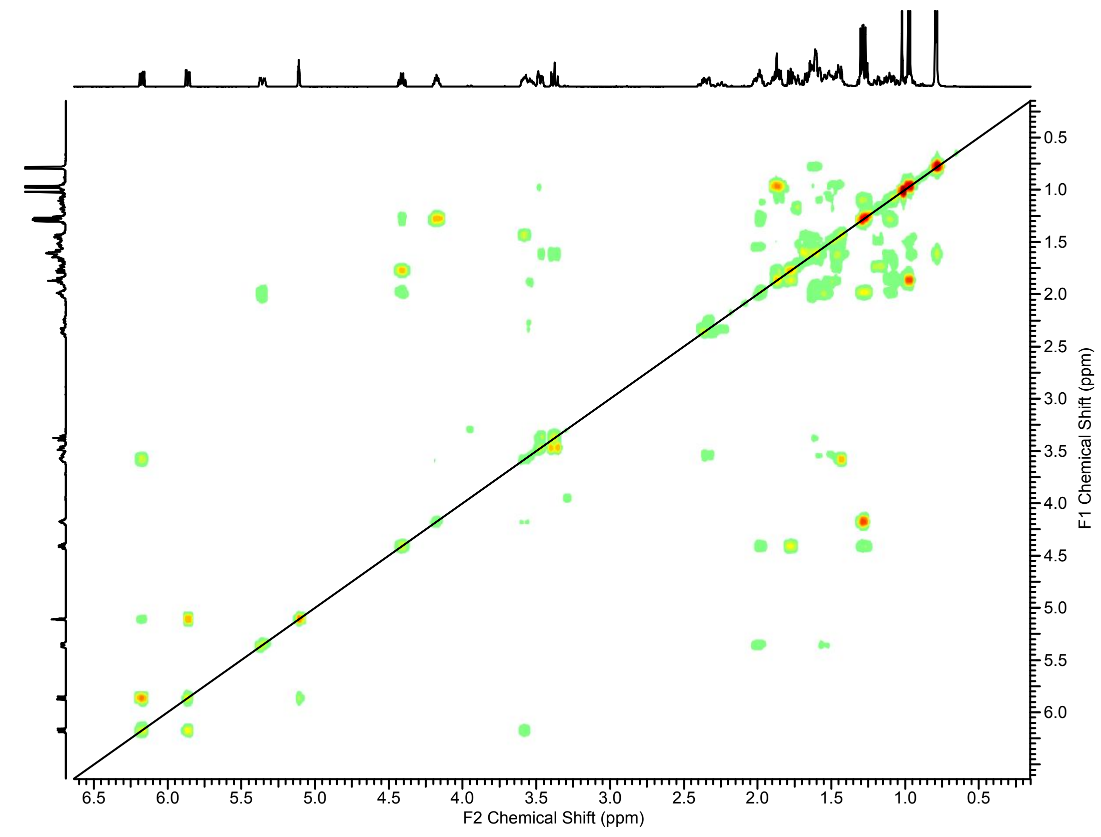

C

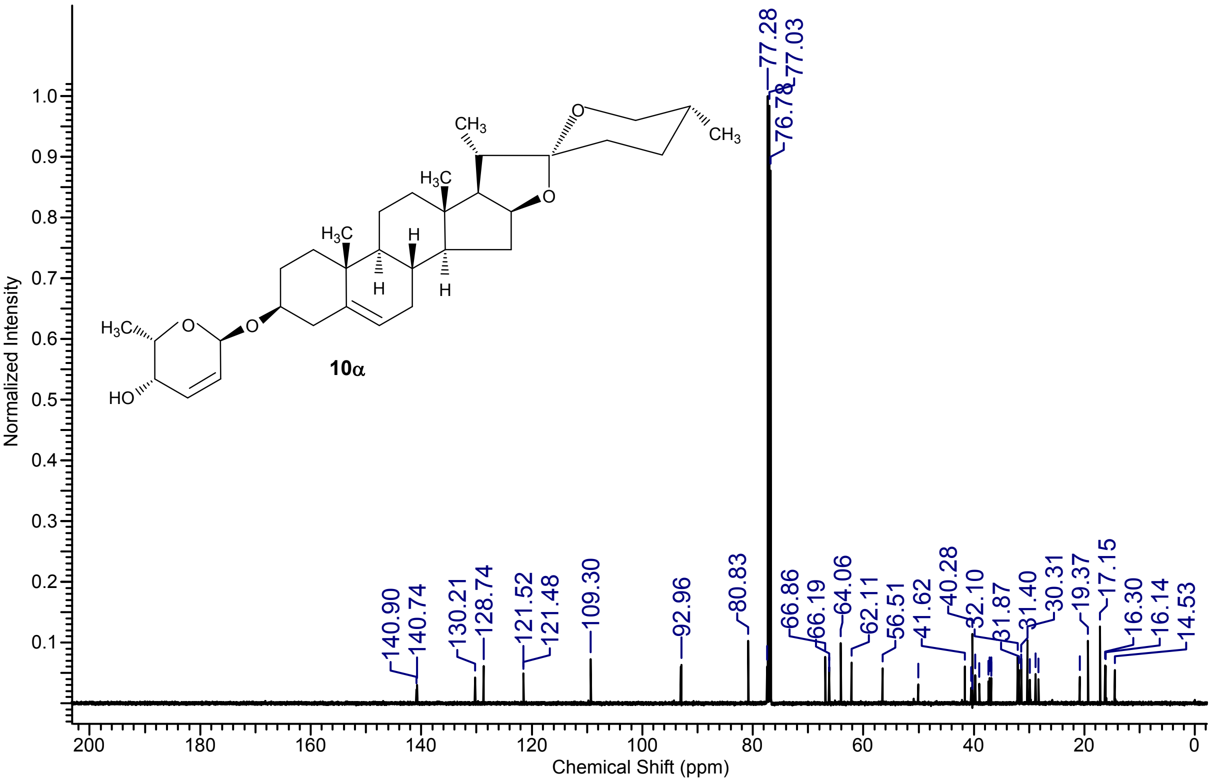

D

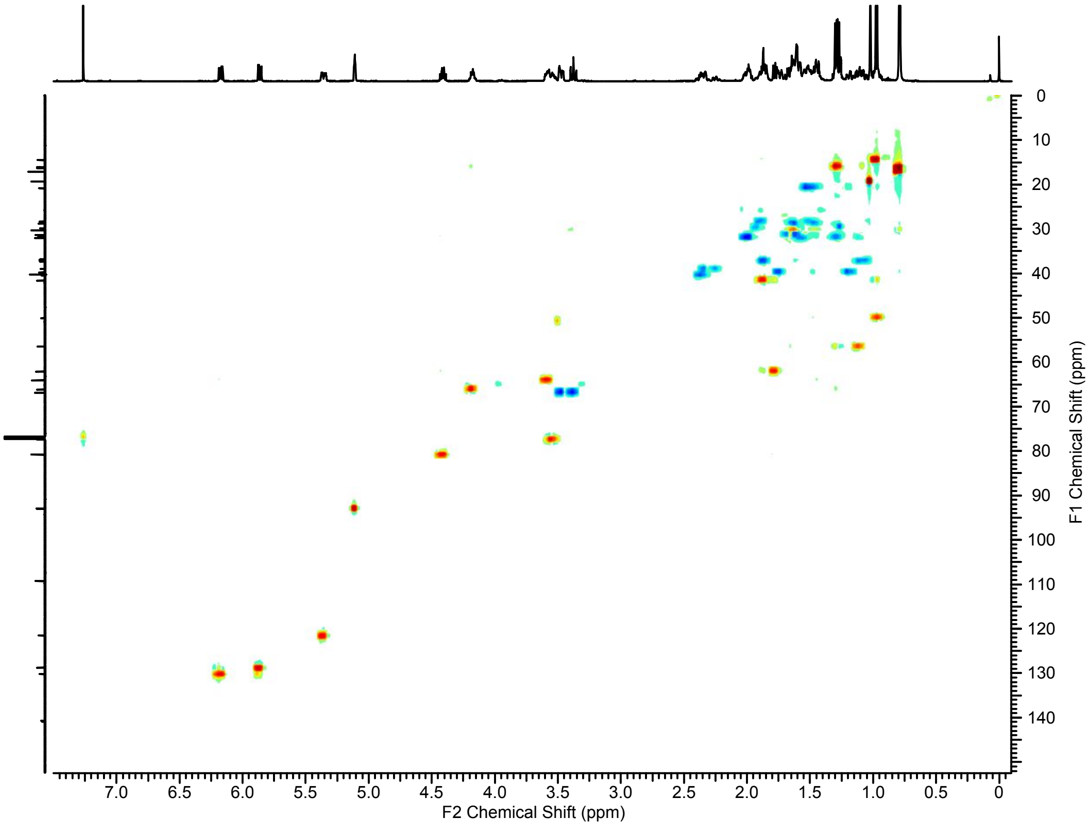

Figure S4. NMR (Chloroform-*d*) spectra of **10α**: ^{1}H NMR (500 MHz) (**A**), gCOSY (**B**), ^{13}C{^{1}H} (125 MHz) (**C**), gHSQC (**D**).

A

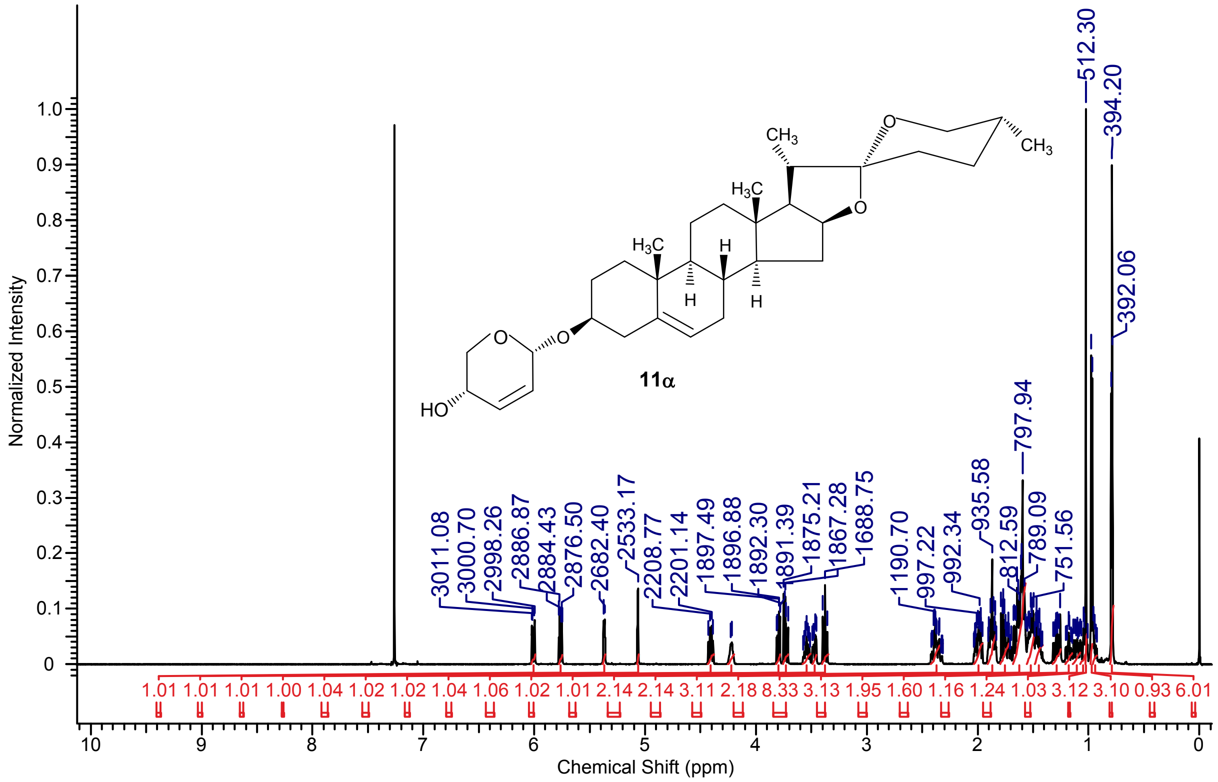
Normalized Intensity
Chemical Shift (ppm)
512.30
394.20
392.06
797.94
3011.08
3000.70
2998.26
2886.87
2884.43
2876.50
2682.40
2533.17
2208.77
2201.14
1897.49
1896.88
1892.30
1891.39
1875.21
1867.28
1688.75
1190.70
997.22
992.34
935.58
812.59
789.09
751.56
1.01 1.01 1.01 1.00 1.04 1.02 1.02 1.04 1.06 1.02 1.01 2.14 2.14 3.11 2.18 8.33 3.13 1.95 1.60 1.16 1.24 1.03 3.12 3.10 0.93 6.01
CH3
H3C
H
O
HO
11α

B

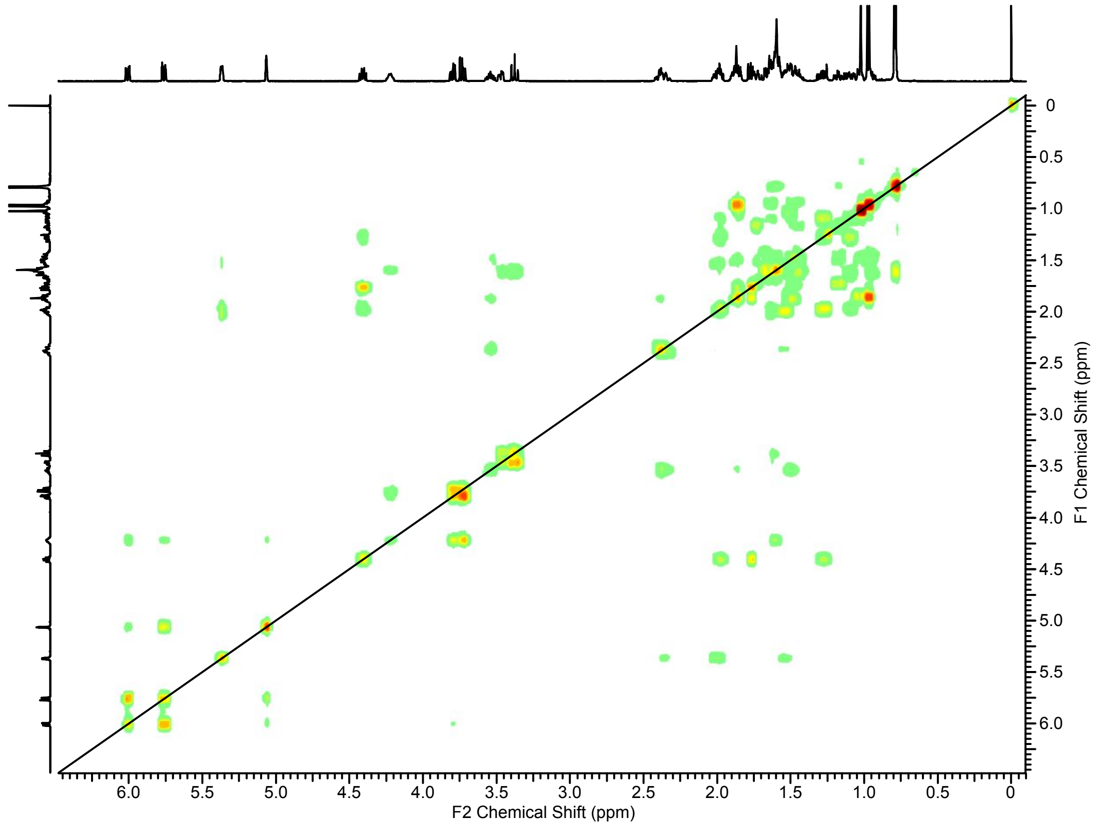
F2 Chemical Shift (ppm)
F1 Chemical Shift (ppm)

C

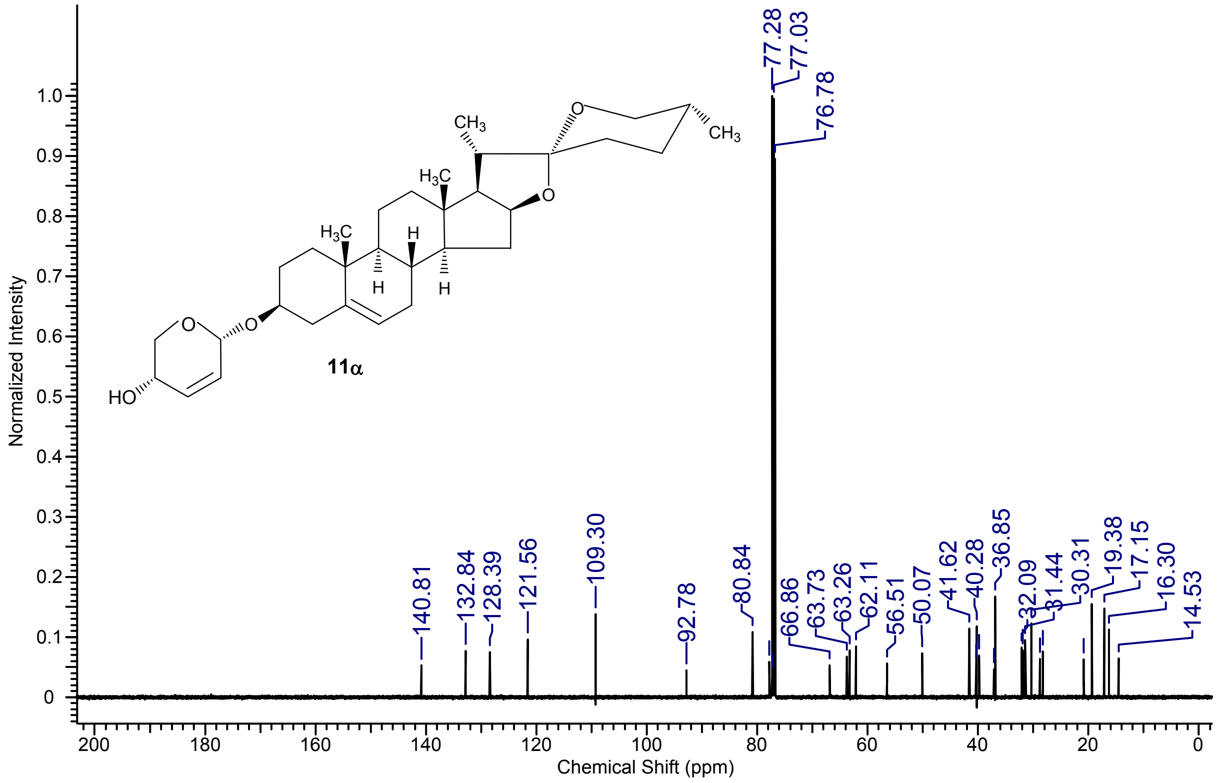

D

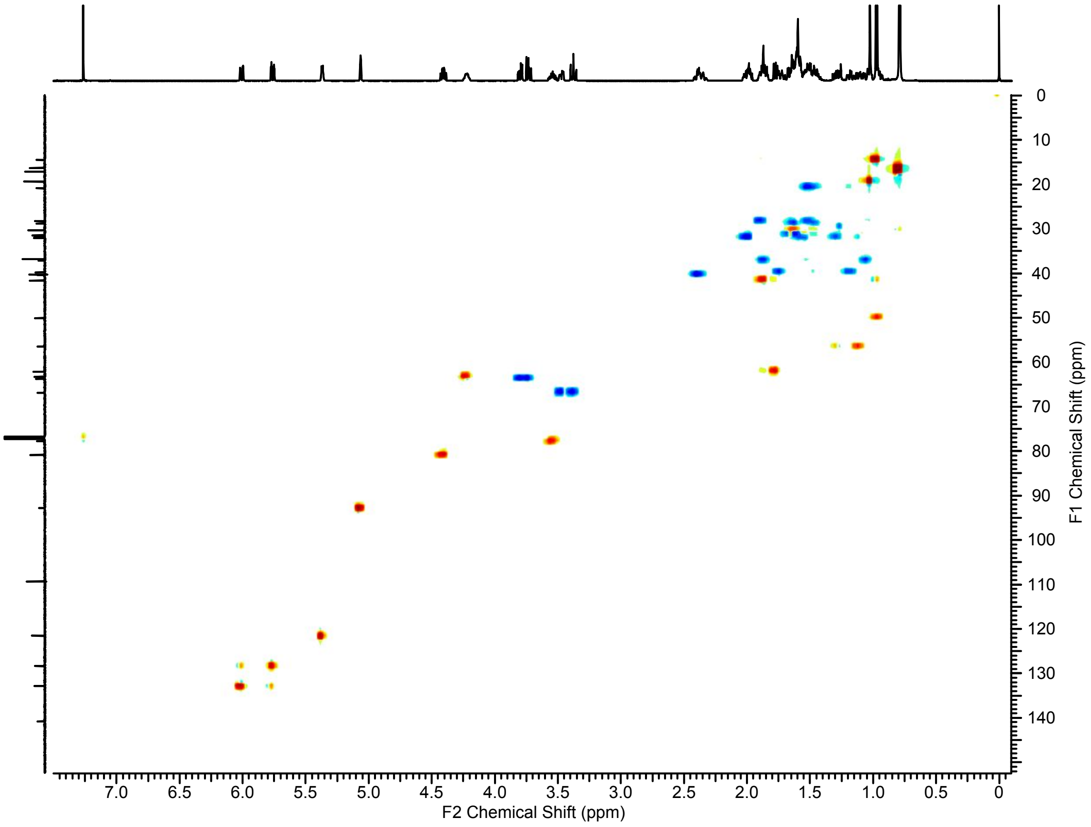

Figure S5. NMR (Chloroform-*d*) spectra of **11α**: ^{1}H NMR (500 MHz) (**A**), gCOSY (**B**), ^{13}C{^{1}H} (125 MHz) (**C**), gHSQC (**D**).

A

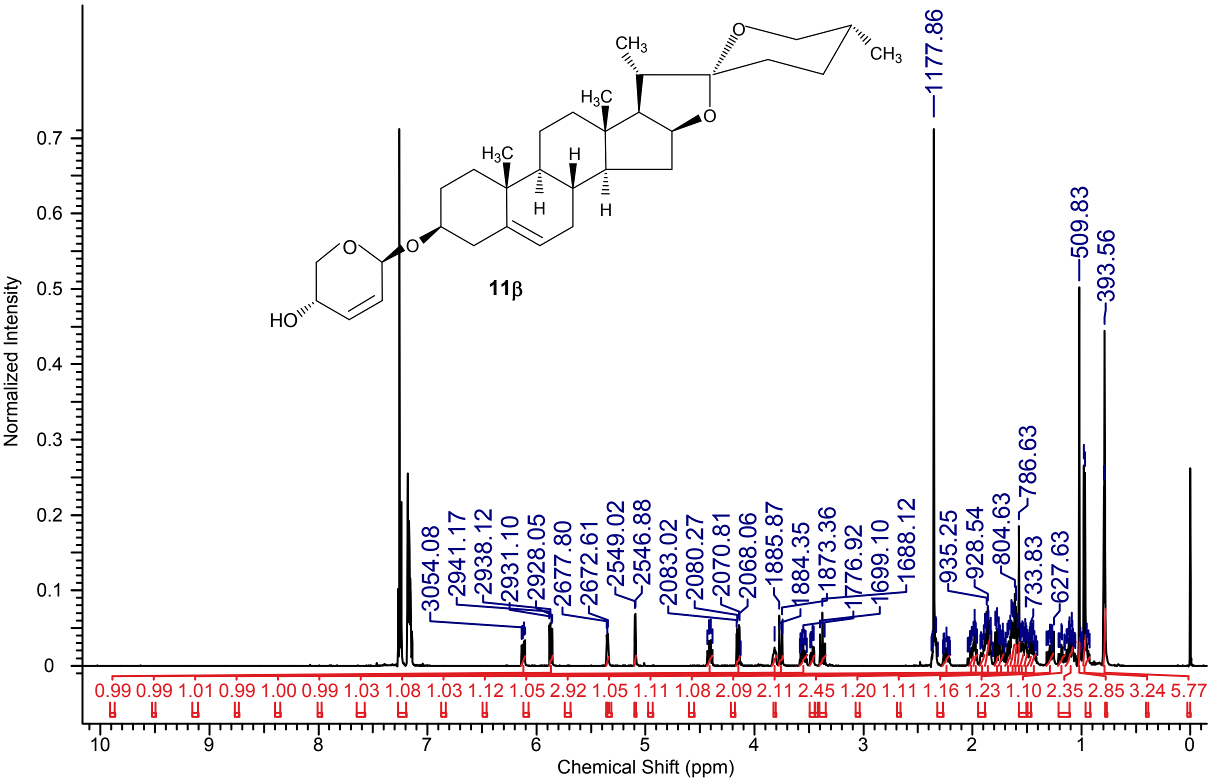

Normalized Intensity
Chemical Shift (ppm)
11β
HO
O
H₃C
CH₃
H
1177.86
509.83
393.56

B

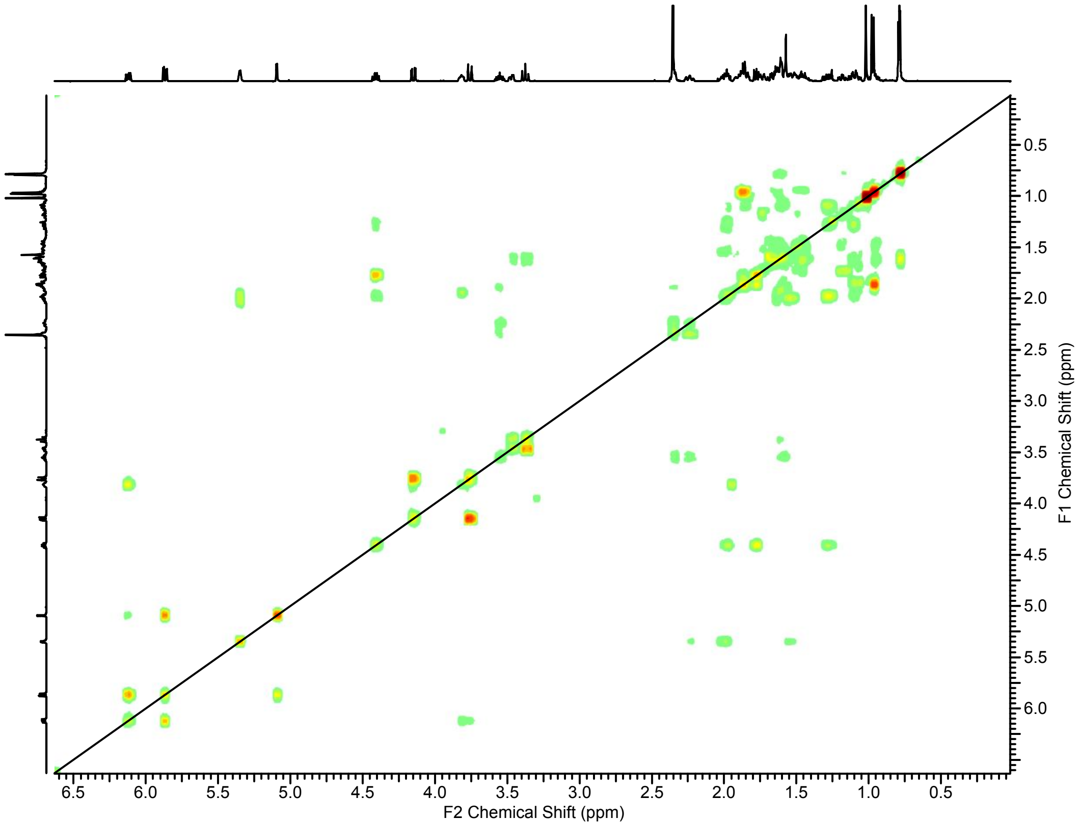

F2 Chemical Shift (ppm)
F1 Chemical Shift (ppm)

C

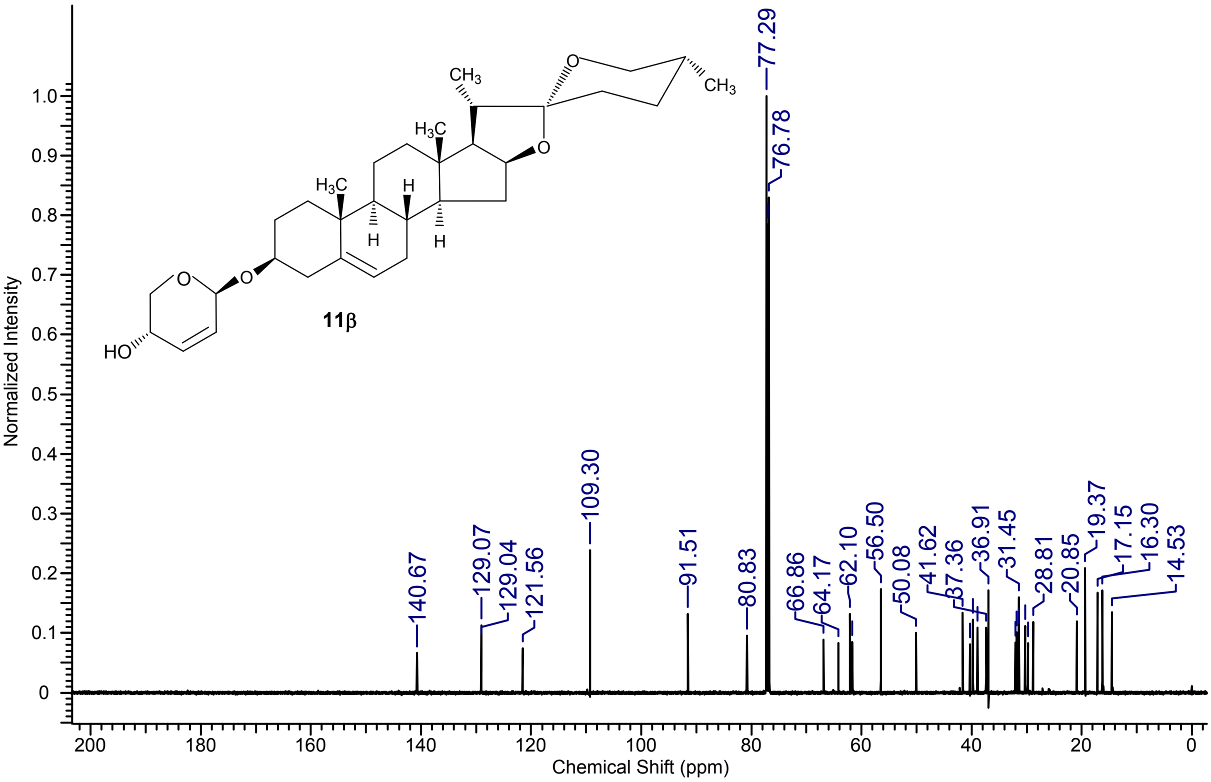

D

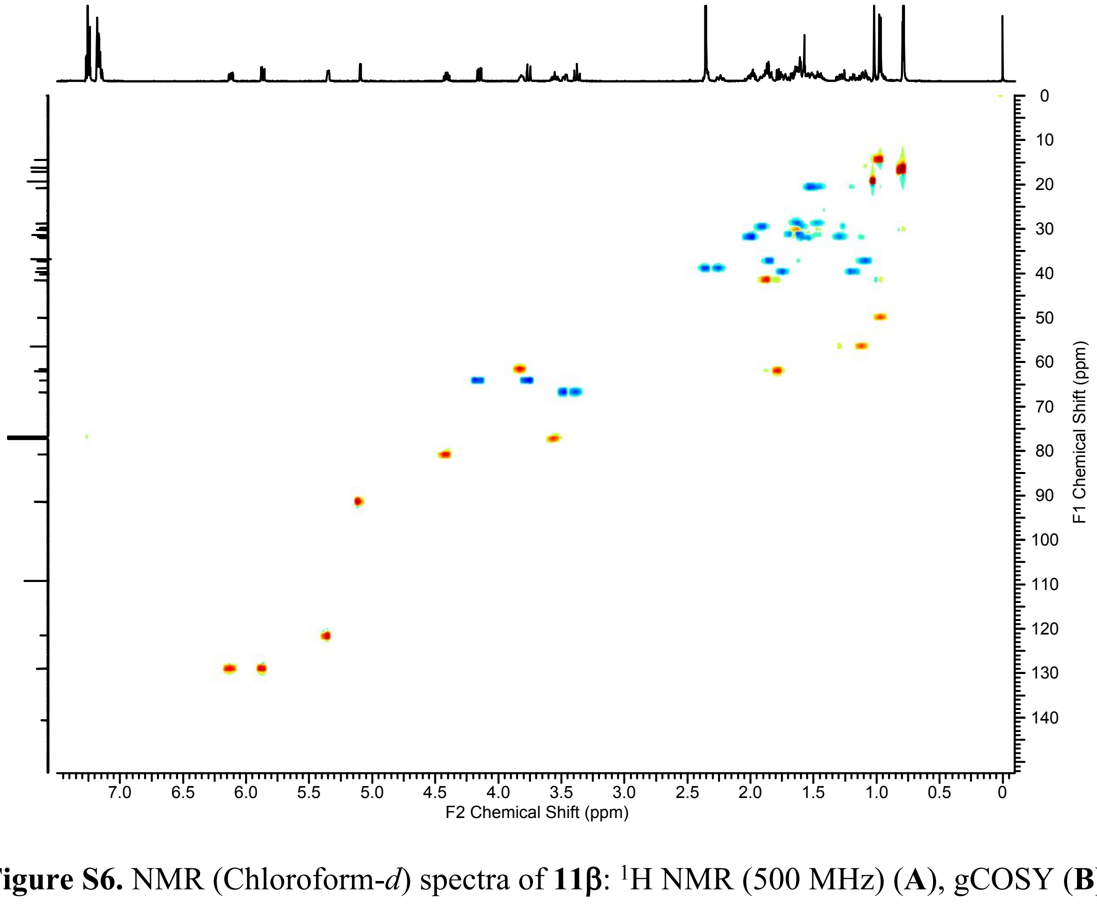

Figure S6. NMR (Chloroform-*d*) spectra of **11β**: ^{1}H NMR (500 MHz) (**A**), gCOSY (**B**), ^{13}C{^{1}H} (125 MHz) (**C**), gHSQC (**D**).

A

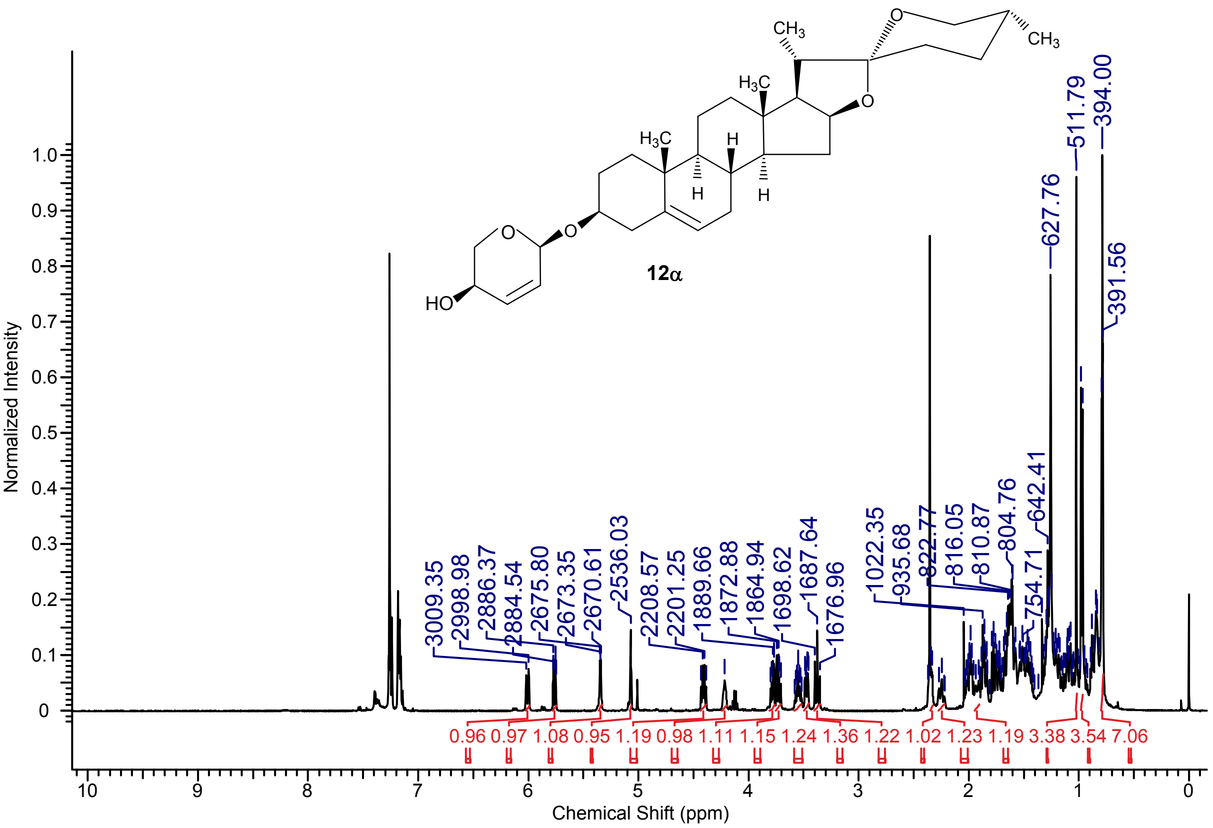

12α
Normalized Intensity
Chemical Shift (ppm)

B

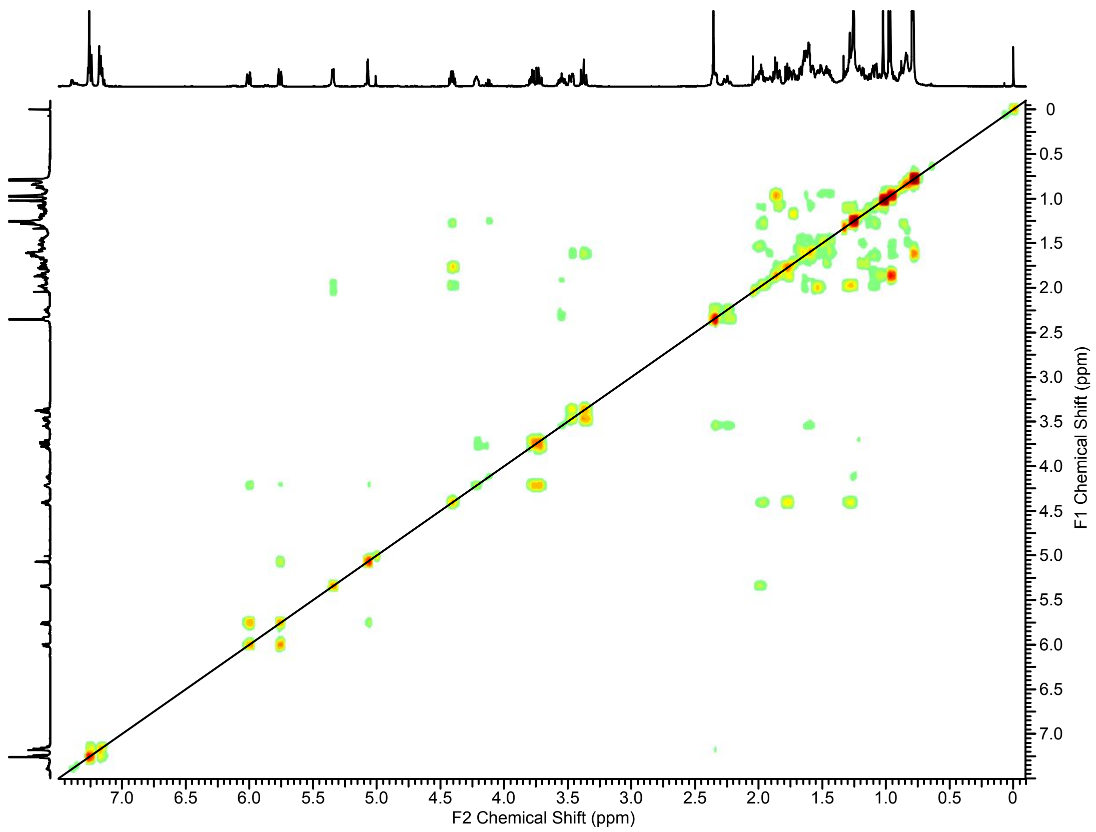

F2 Chemical Shift (ppm)
F1 Chemical Shift (ppm)

C

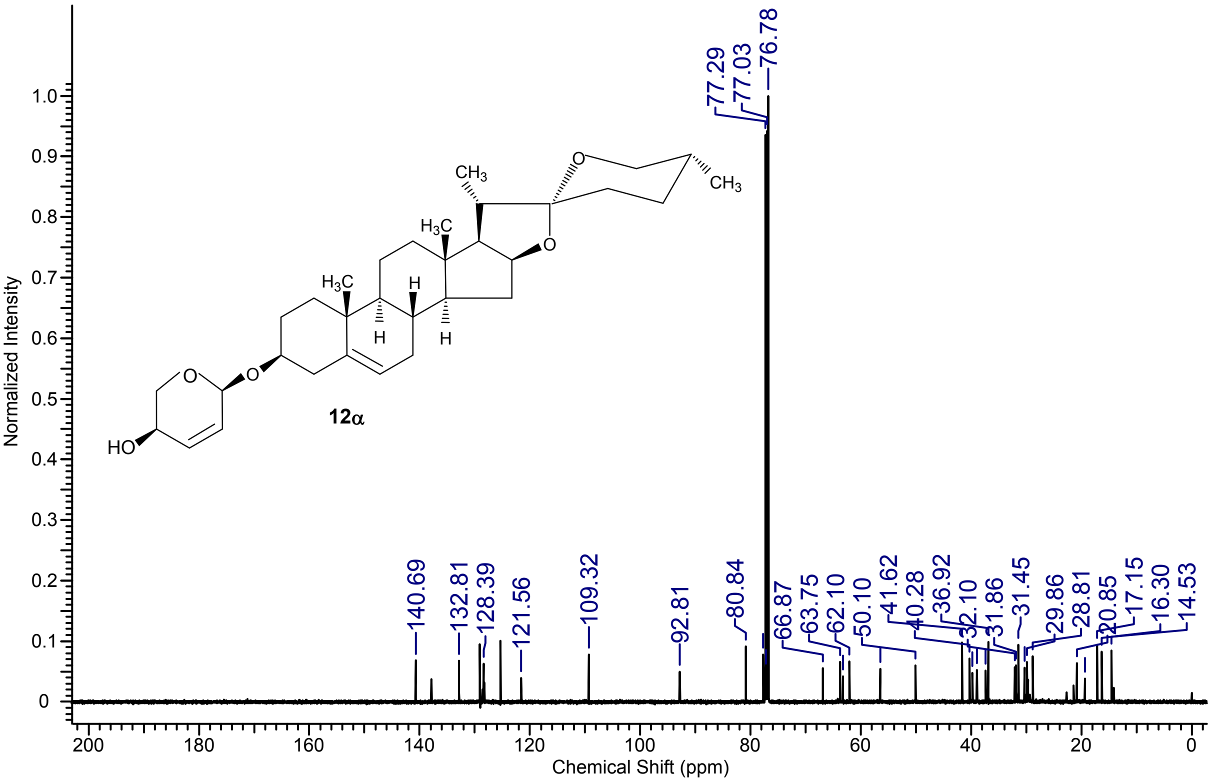

D

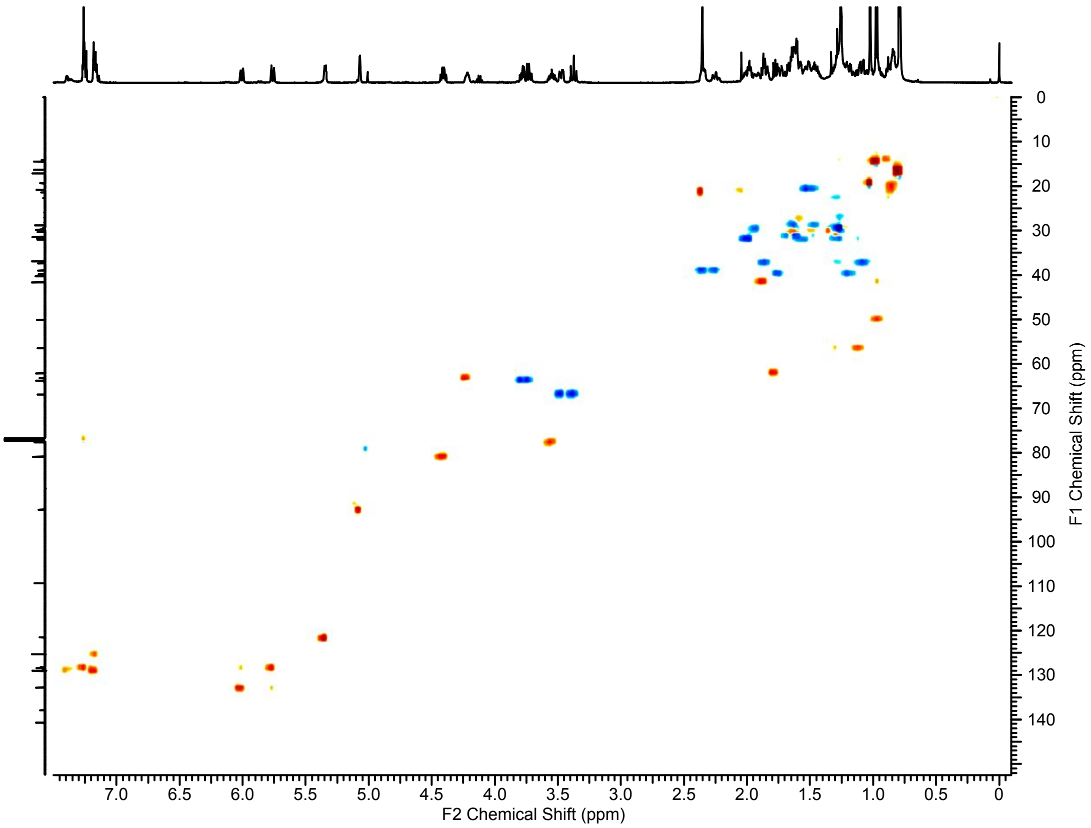

Figure S7. NMR (Chloroform-*d*) spectra of **12α**: ^{1}H NMR (500 MHz) (**A**), gCOSY (**B**), ^{13}C{^{1}H} (125 MHz) (**C**), gHSQC (**D**).

A

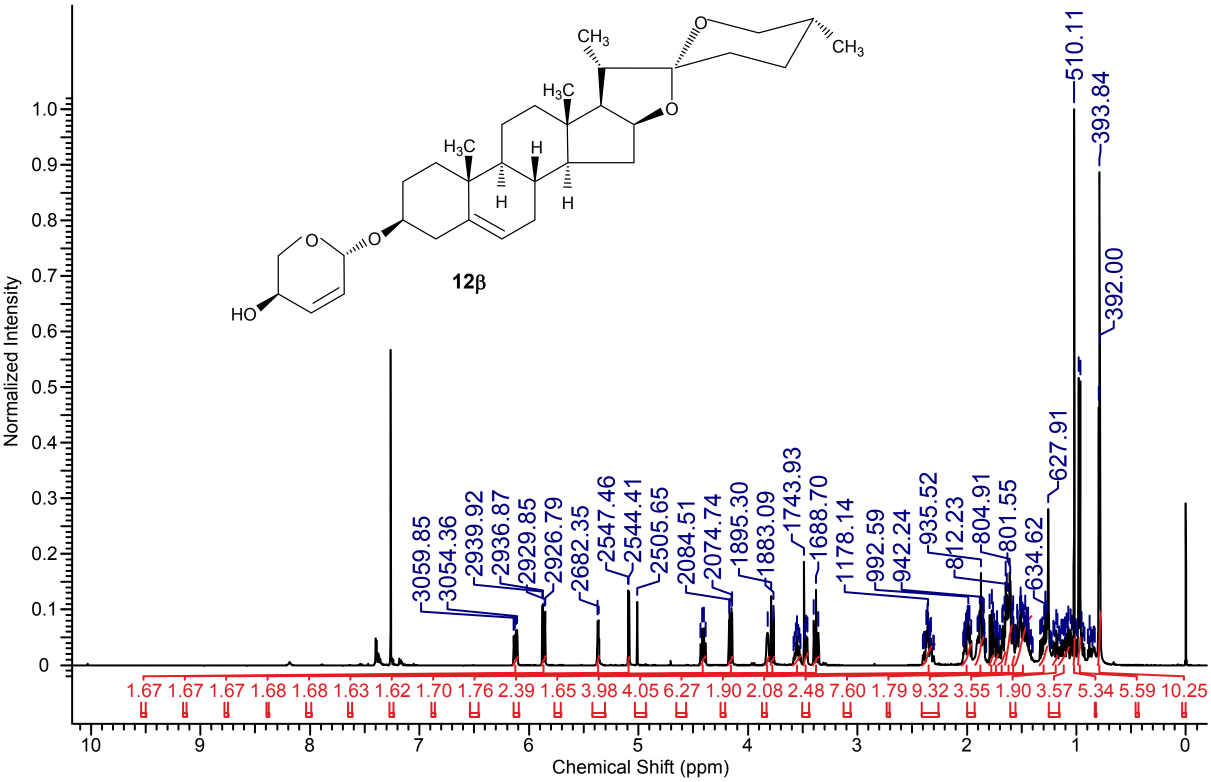

12β
HO
O
CH3
H3C
H
Normalized Intensity
Chemical Shift (ppm)
3059.85
3054.36
2939.92
2936.87
2929.85
2926.79
2682.35
2547.46
2544.41
2505.65
2084.51
2074.74
1895.30
1883.09
1743.93
1688.70
1178.14
992.59
942.24
935.52
812.23
804.91
801.55
634.62
627.91
510.11
393.84
392.00
1.67 1.67 1.67 1.68 1.68 1.63 1.62 1.70 1.76 2.39 1.65 3.98 4.05 6.27 1.90 2.08 2.48 7.60 1.79 9.32 3.55 1.90 3.57 5.34 5.59 10.25

B

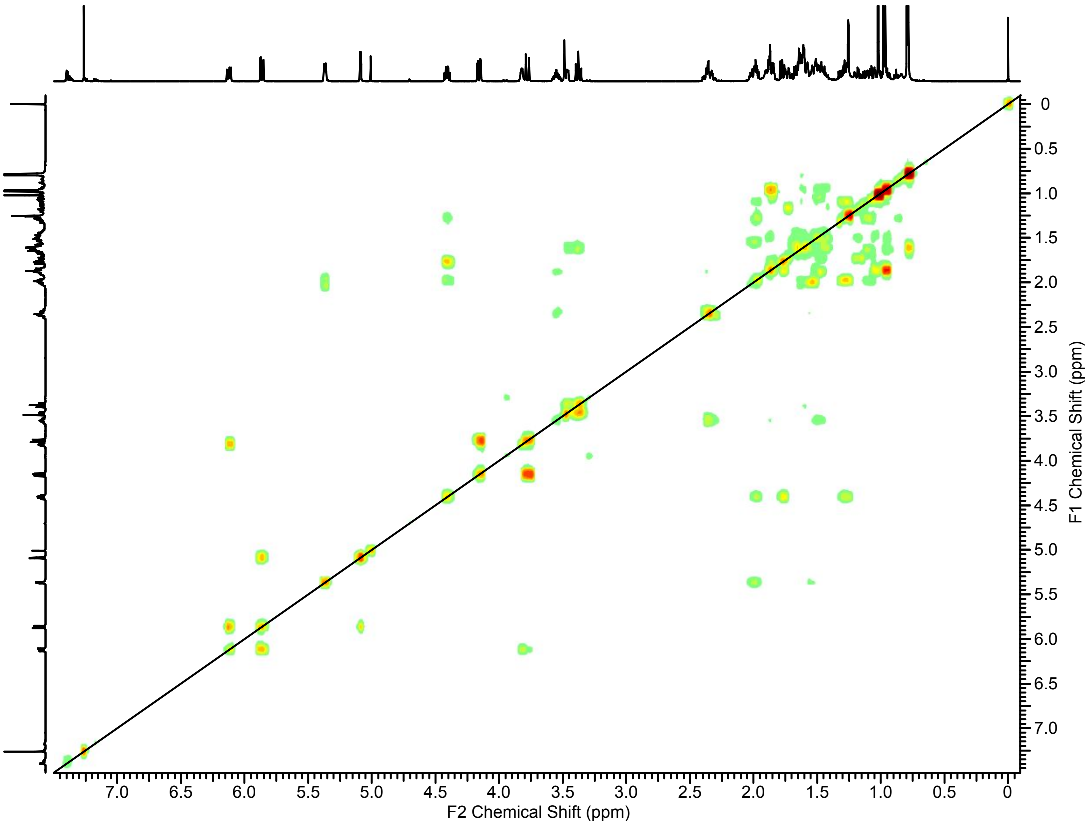

F2 Chemical Shift (ppm)
F1 Chemical Shift (ppm)

C

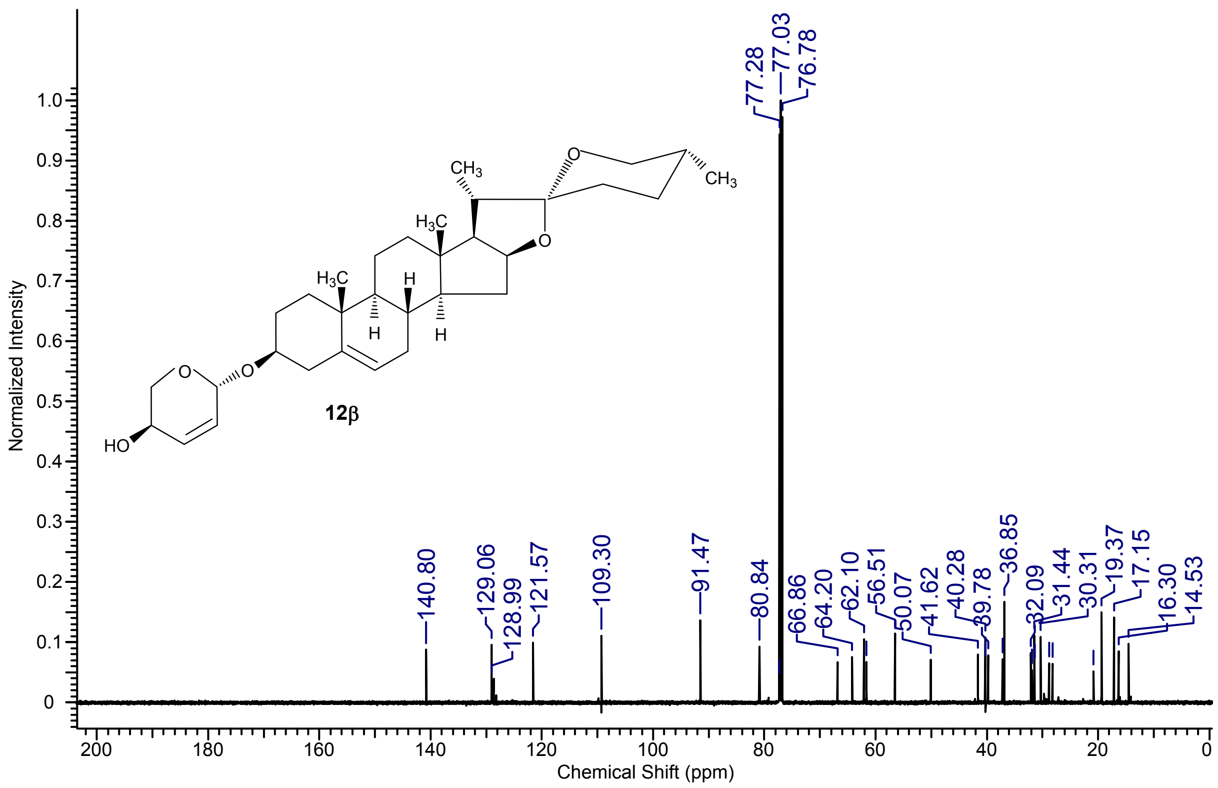

D

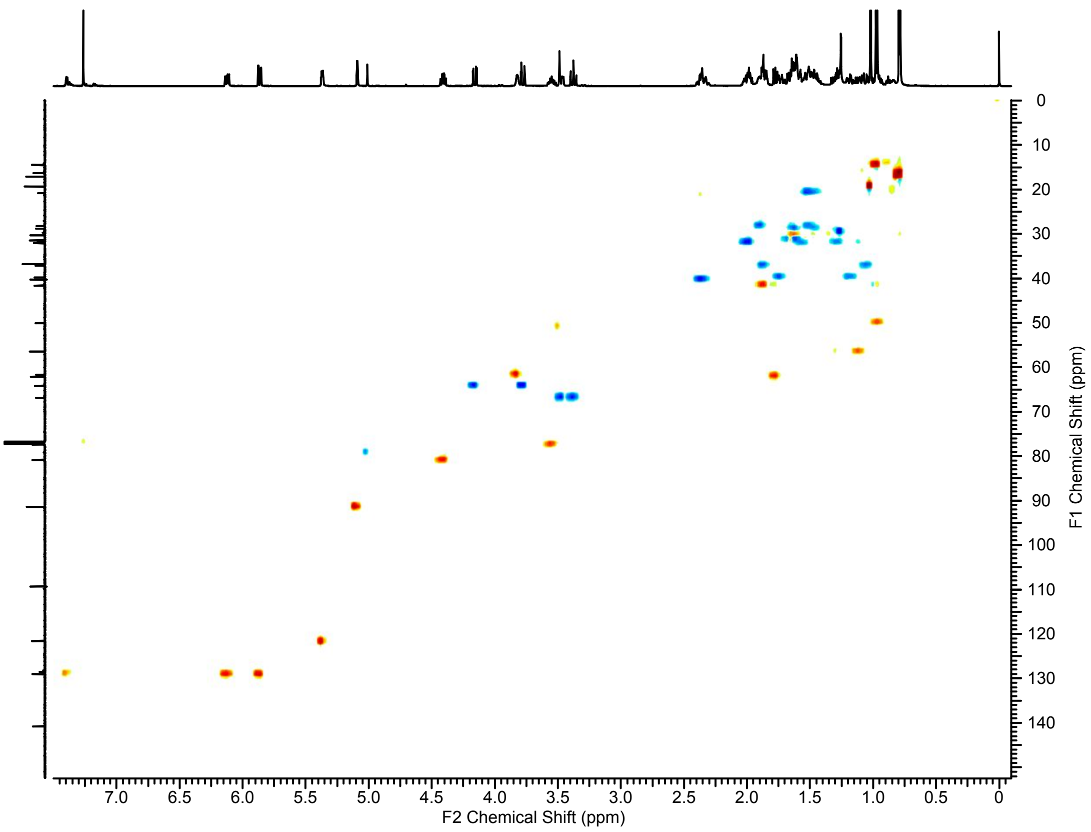

Figure S8. NMR (Chloroform-*d*) spectra of **12β**: ^{1}H NMR (500 MHz) (**A**), gCOSY (**B**), ^{13}C{^{1}H} (125 MHz) (**C**), gHSQC (**D**).

A

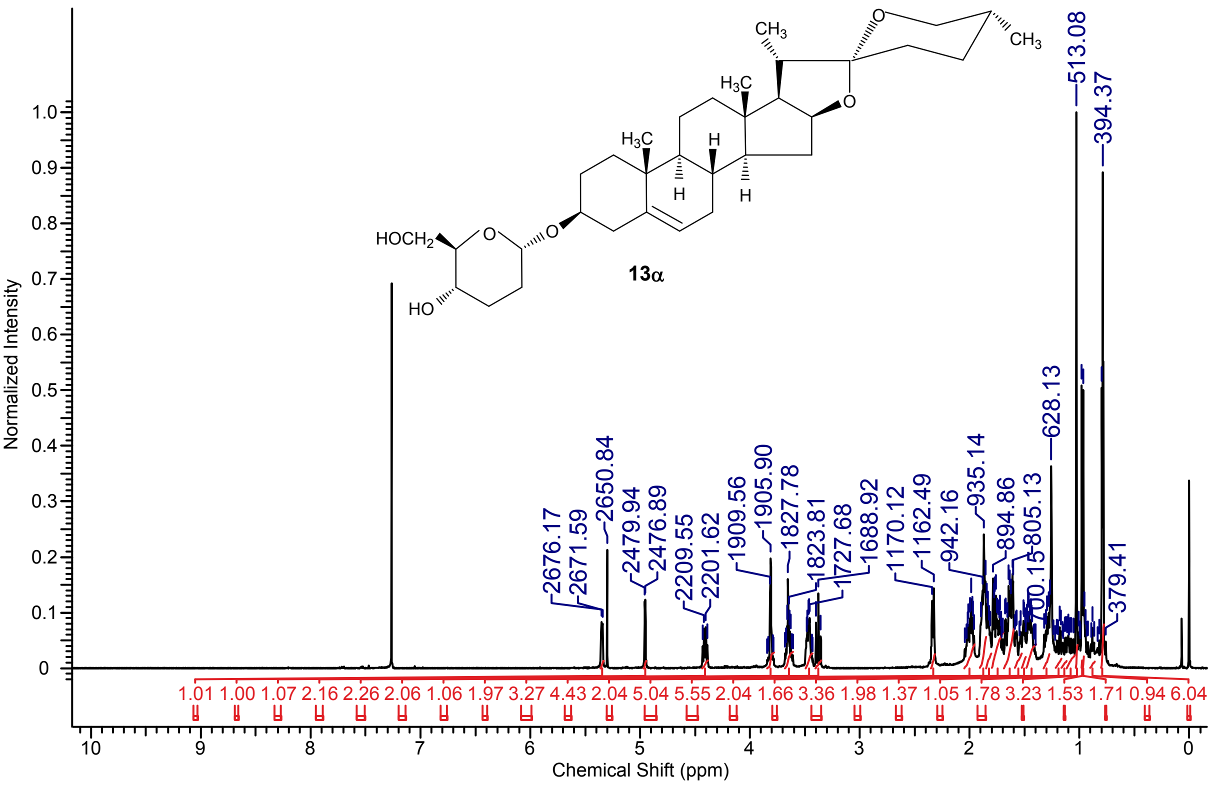
13α
HOCH2
HO
CH3
H3C
Normalized Intensity
Chemical Shift (ppm)
2676.17
2671.59
2650.84
2479.94
2476.89
2209.55
2201.62
1909.56
1905.90
1827.78
1823.81
1727.68
1688.92
1170.12
1162.49
942.16
935.14
894.86
805.13
628.13
513.08
394.37
379.41
1.01 1.00 1.07 2.16 2.26 2.06 1.06 1.97 3.27 4.43 2.04 5.04 5.55 2.04 1.66 3.36 1.98 1.37 1.05 1.78 3.23 1.53 1.71 0.94 6.04

B

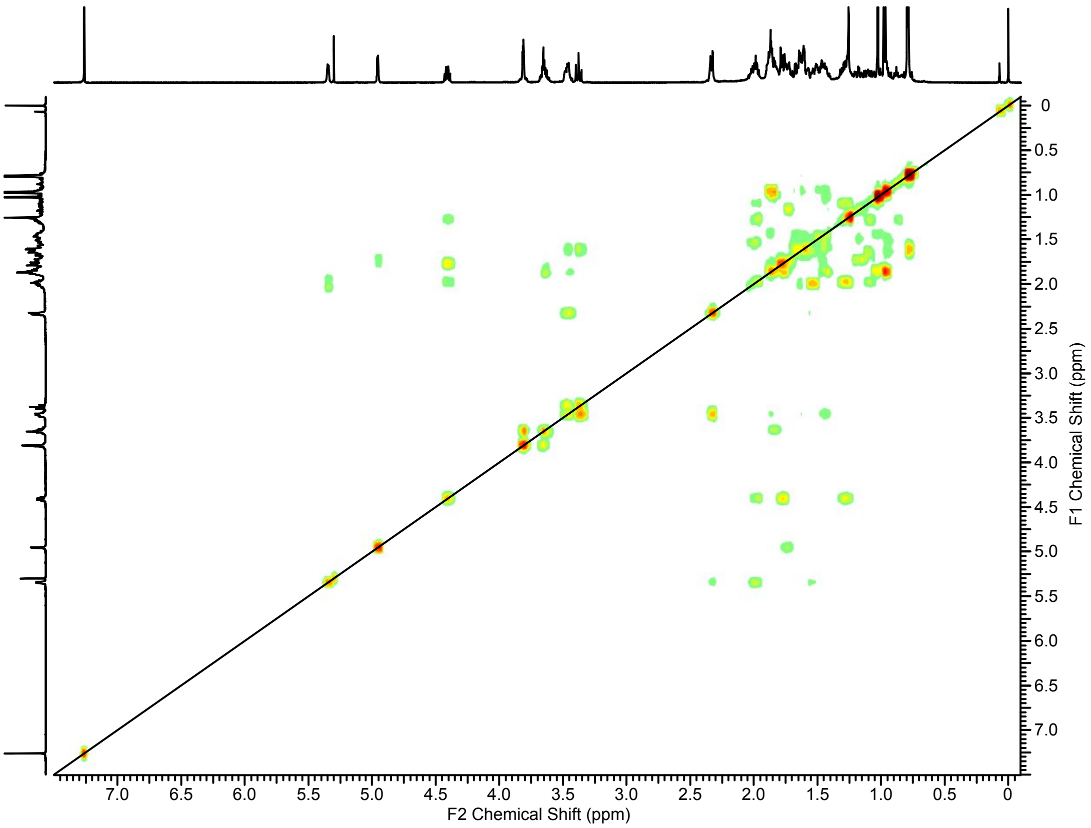
F2 Chemical Shift (ppm)
F1 Chemical Shift (ppm)

C

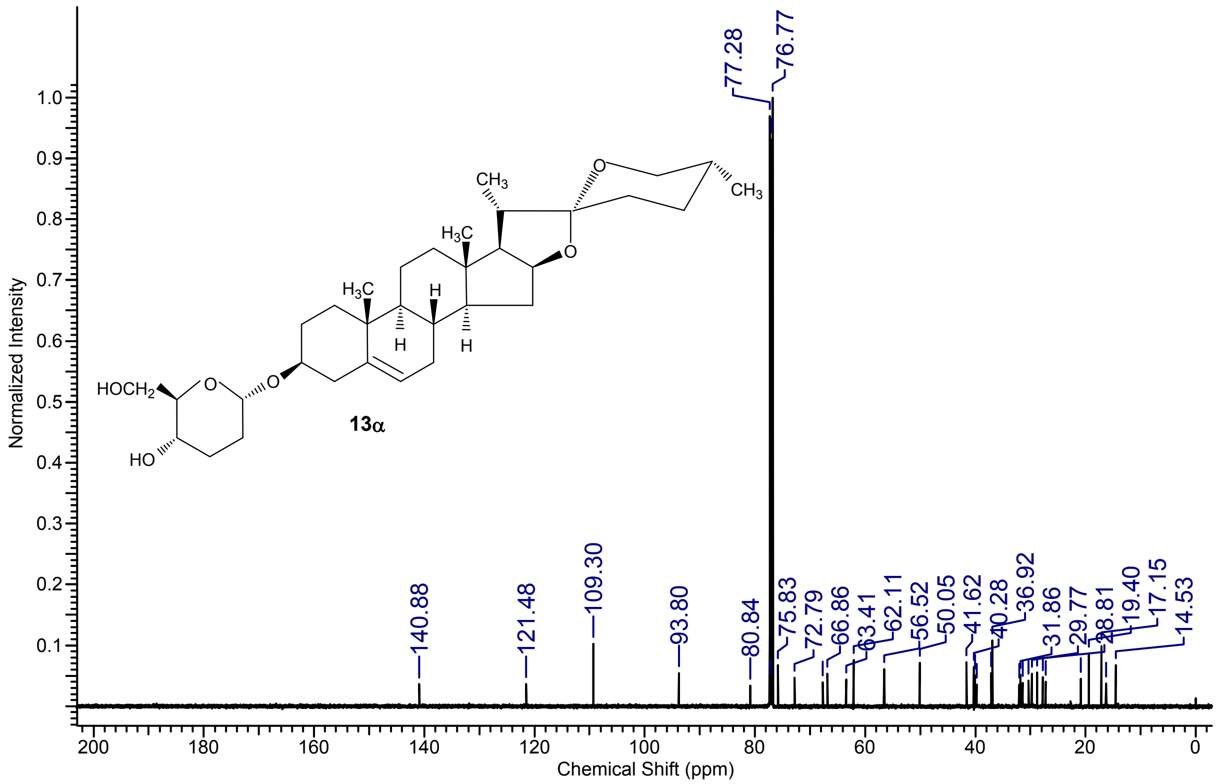

D

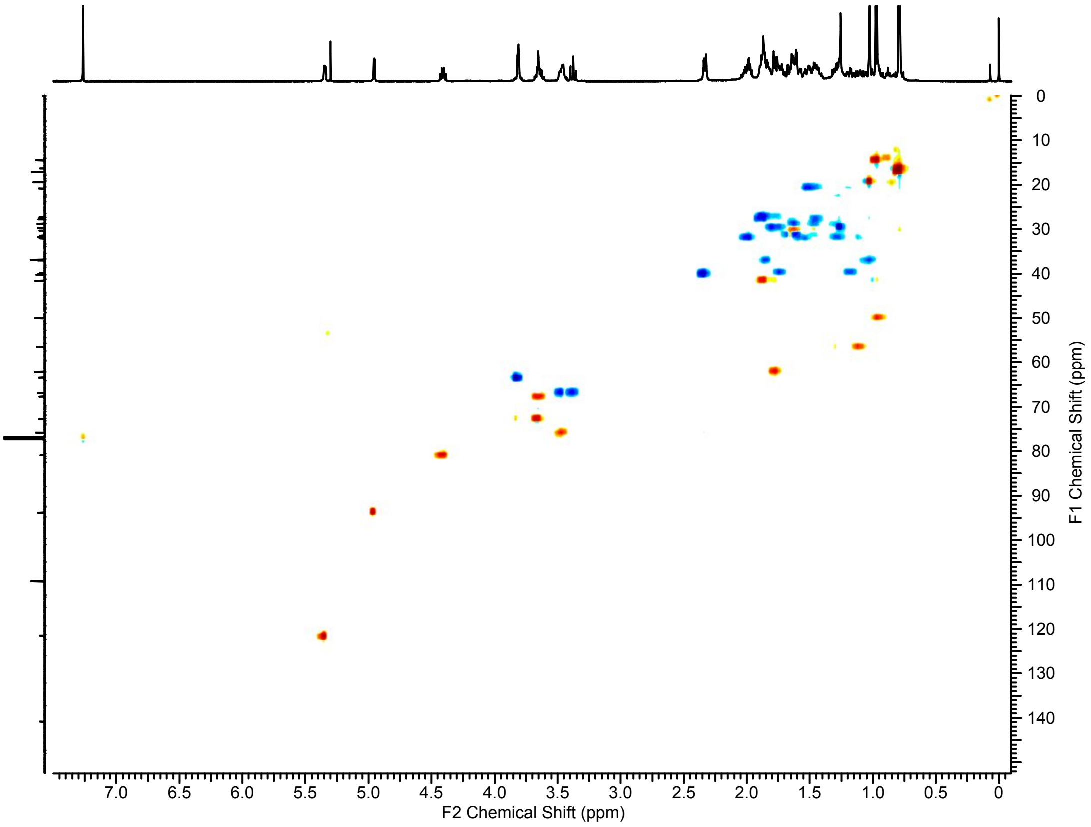

Figure S9. NMR (Chloroform-*d*) spectra of **13α**: ^{1}H NMR (500 MHz) (**A**), gCOSY (**B**), ^{13}C{^{1}H} (125 MHz) (**C**), gHSQC (**D**).

A

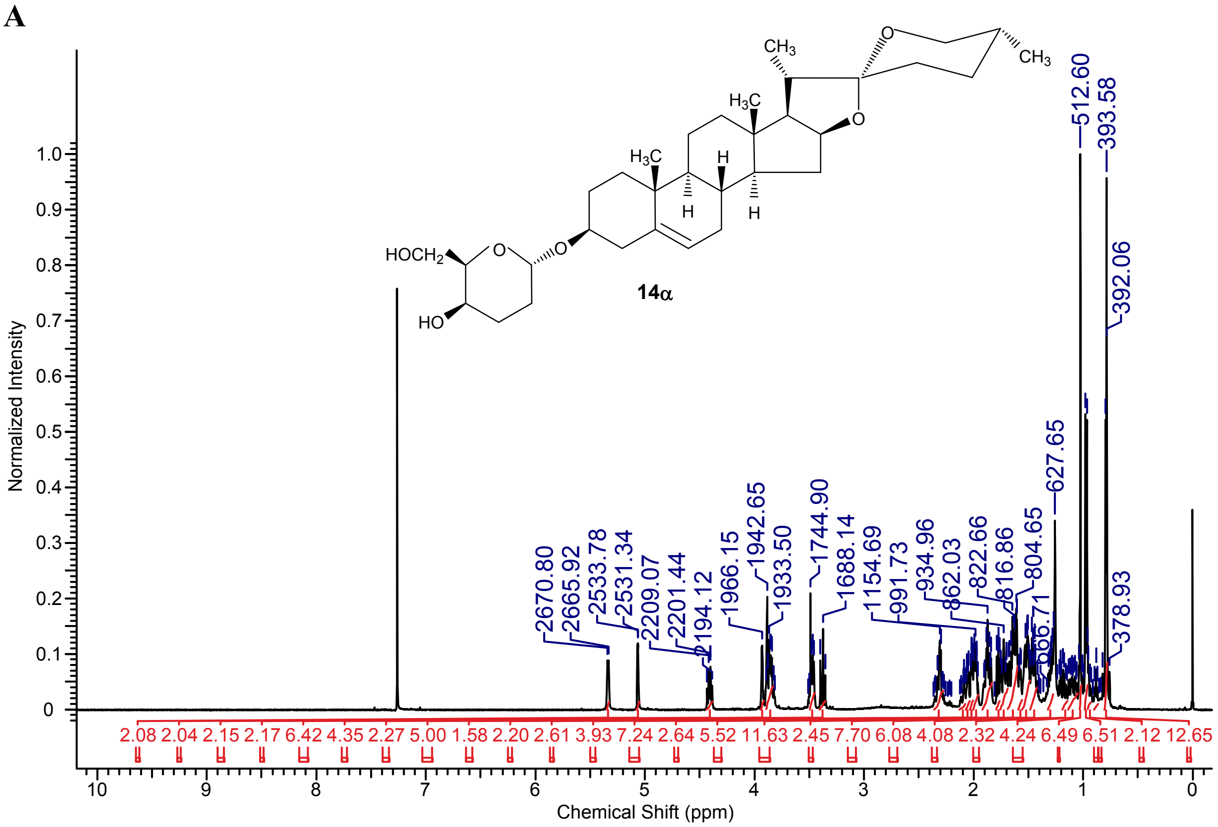
14α
HOCH2
HO
CH3
H3C
H
Normalized Intensity
Chemical Shift (ppm)
512.60
393.58
392.06
627.65
378.93
2.08 2.04 2.15 2.17 6.42 4.35 2.27 5.00 1.58 2.20 2.61 3.93 7.24 2.64 5.52 11.63 2.45 7.70 6.08 4.08 2.32 4.24 6.49 6.51 2.12 12.65

B

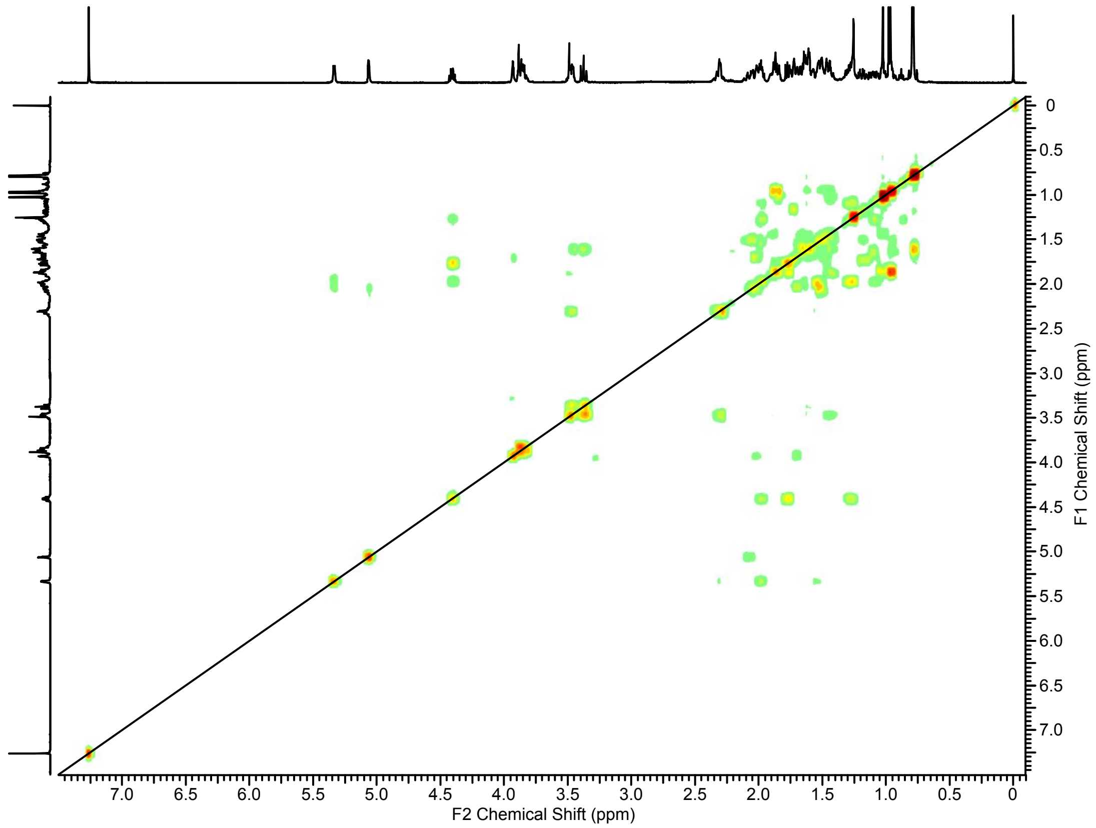
F2 Chemical Shift (ppm)
F1 Chemical Shift (ppm)

C

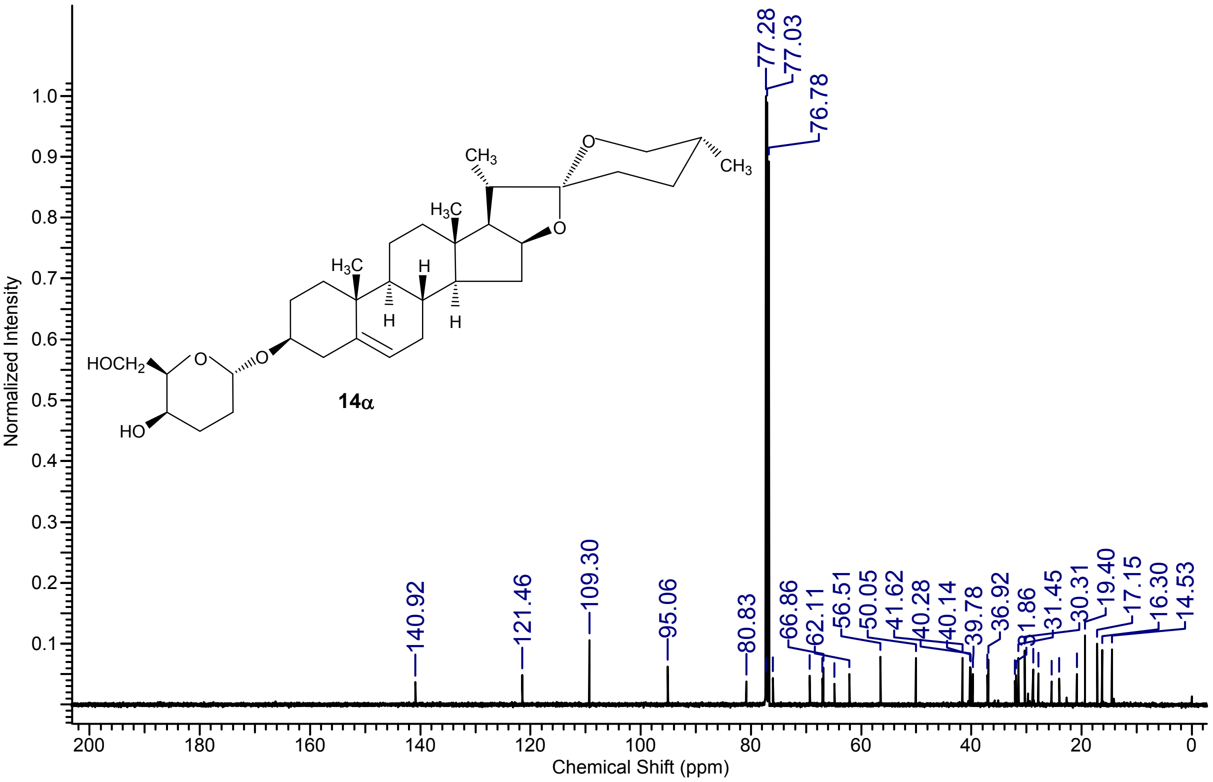

D

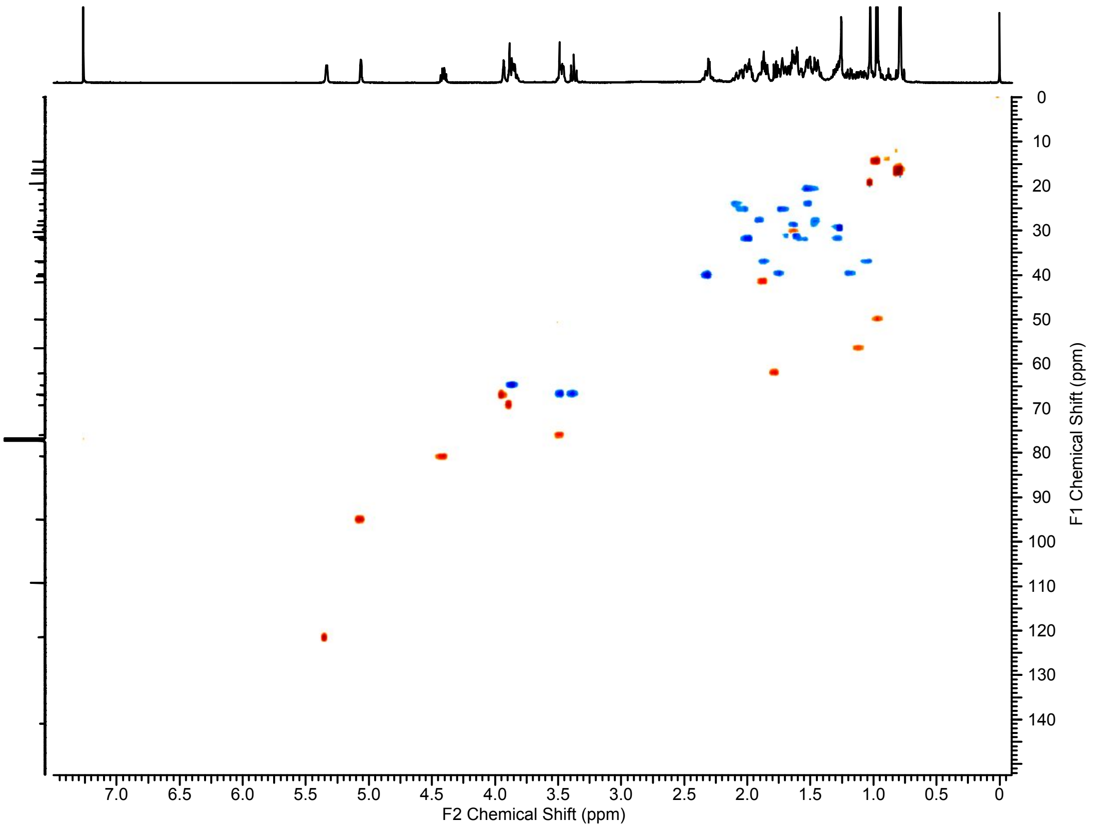

Figure S10. NMR (Chloroform-*d*) spectra of **14α**: ^{1}H NMR (500 MHz) (**A**), gCOSY (**B**), ^{13}C{^{1}H} (125 MHz) (**C**), gHSQC (**D**).

A

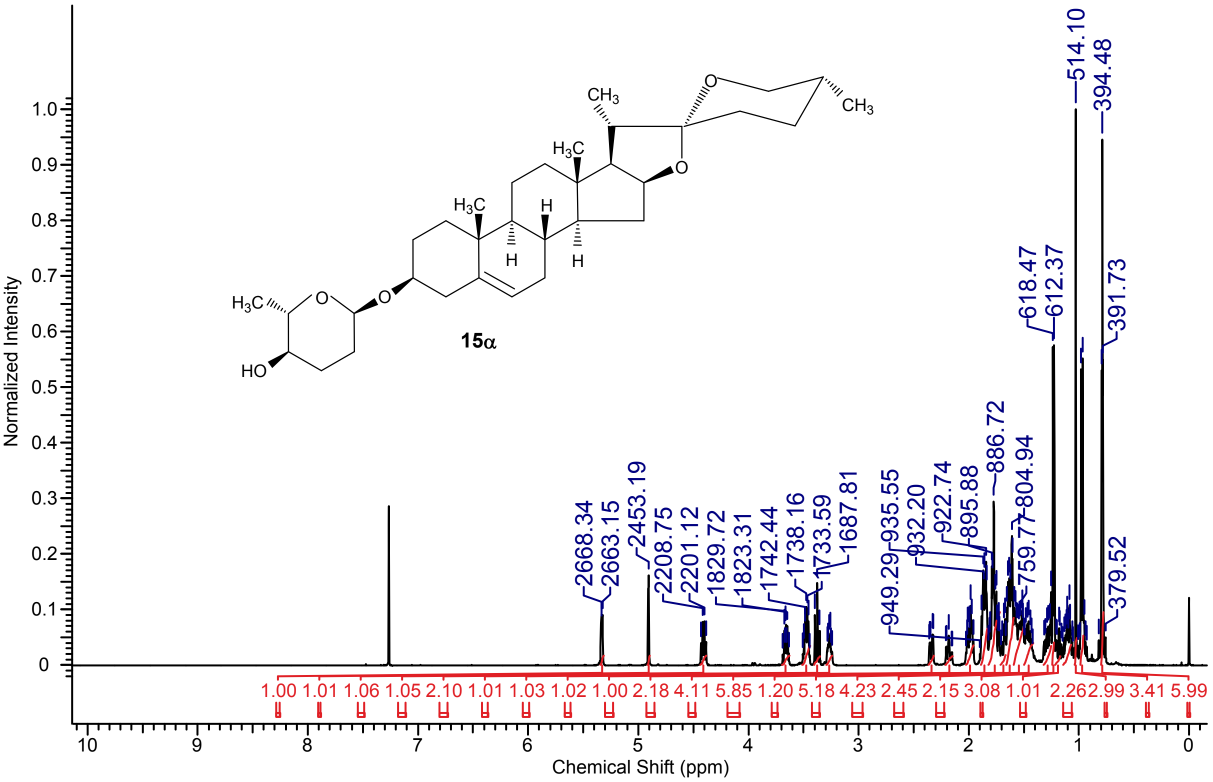

15α
Normalized Intensity
Chemical Shift (ppm)

B

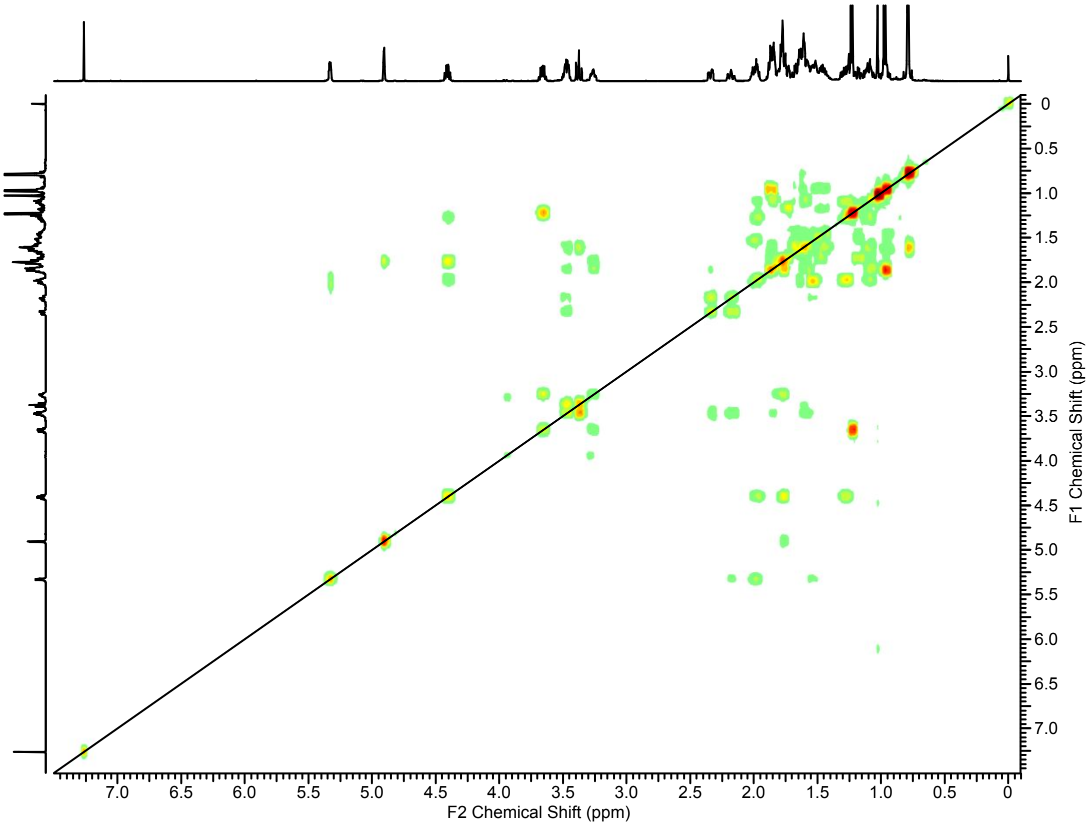

F2 Chemical Shift (ppm)
F1 Chemical Shift (ppm)

C

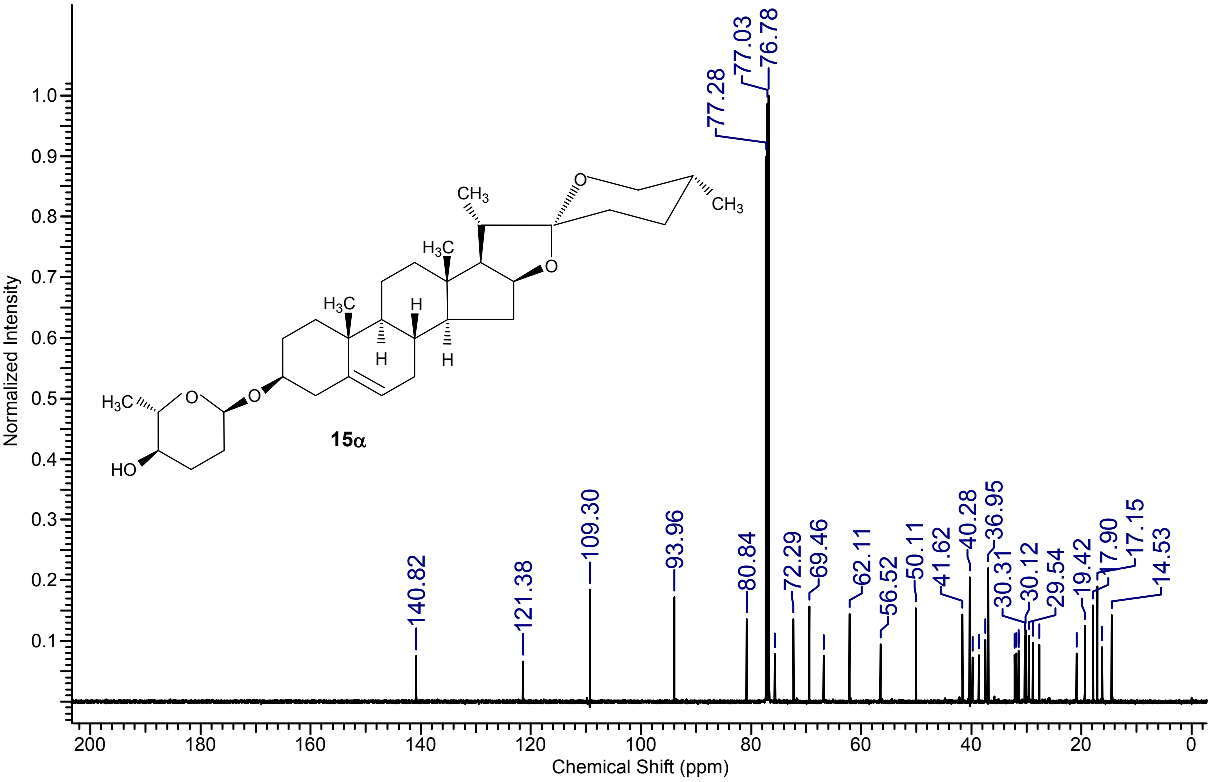

D

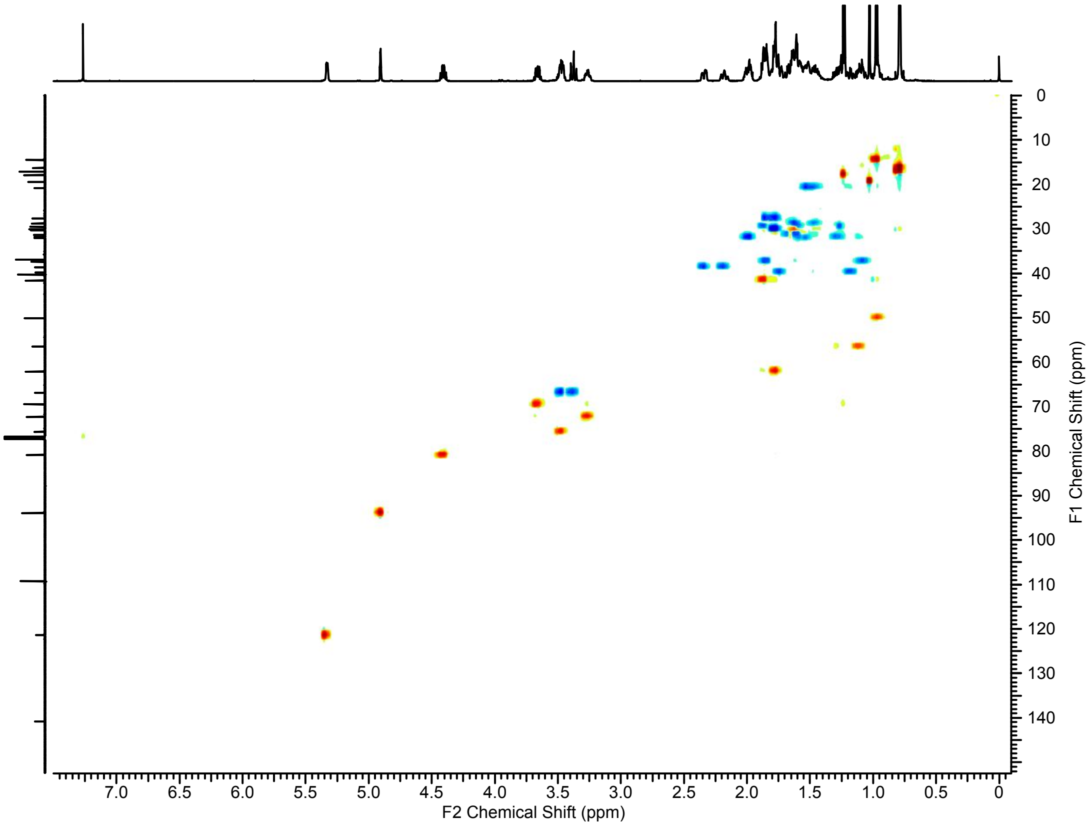

Figure S11. NMR (Chloroform-*d*) spectra of **15α**: ^{1}H NMR (500 MHz) (**A**), gCOSY (**B**), ^{13}C{^{1}H} (125 MHz) (**C**), gHSQC (**D**).

A

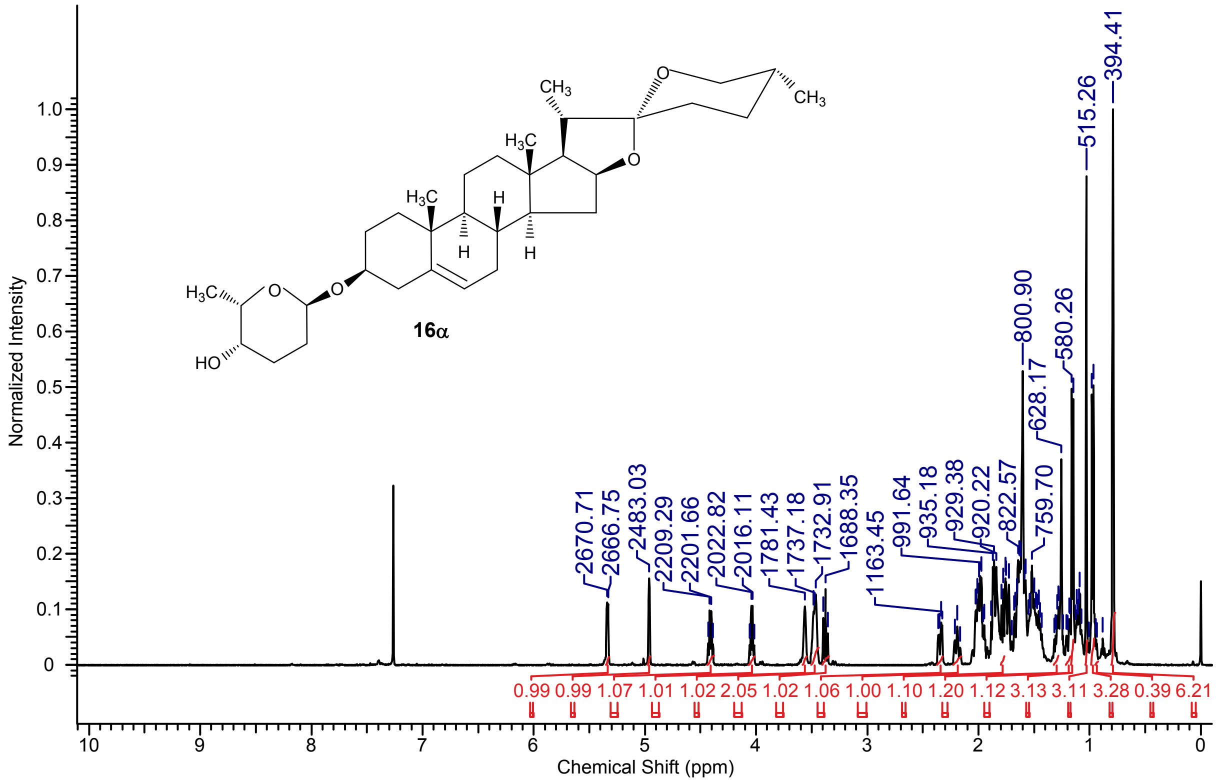

16α
H3C
O
HO
O
H3C
H3C
H
H
H
H
CH3
CH3
O
O
Normalized Intensity
1.0
0.9
0.8
0.7
0.6
0.5
0.4
0.3
0.2
0.1
0
2670.71
2666.75
2483.03
2209.29
2201.66
2022.82
2016.11
1781.43
1737.18
1732.91
1688.35
1163.45
991.64
935.18
929.38
920.22
822.57
800.90
759.70
628.17
580.26
515.26
394.41
0.99 0.99 1.07 1.01 1.02 2.05 1.02 1.06 1.00 1.10 1.20 1.12 3.13 3.11 3.28 0.39 6.21
10 9 8 7 6 5 4 3 2 1 0
Chemical Shift (ppm)

B

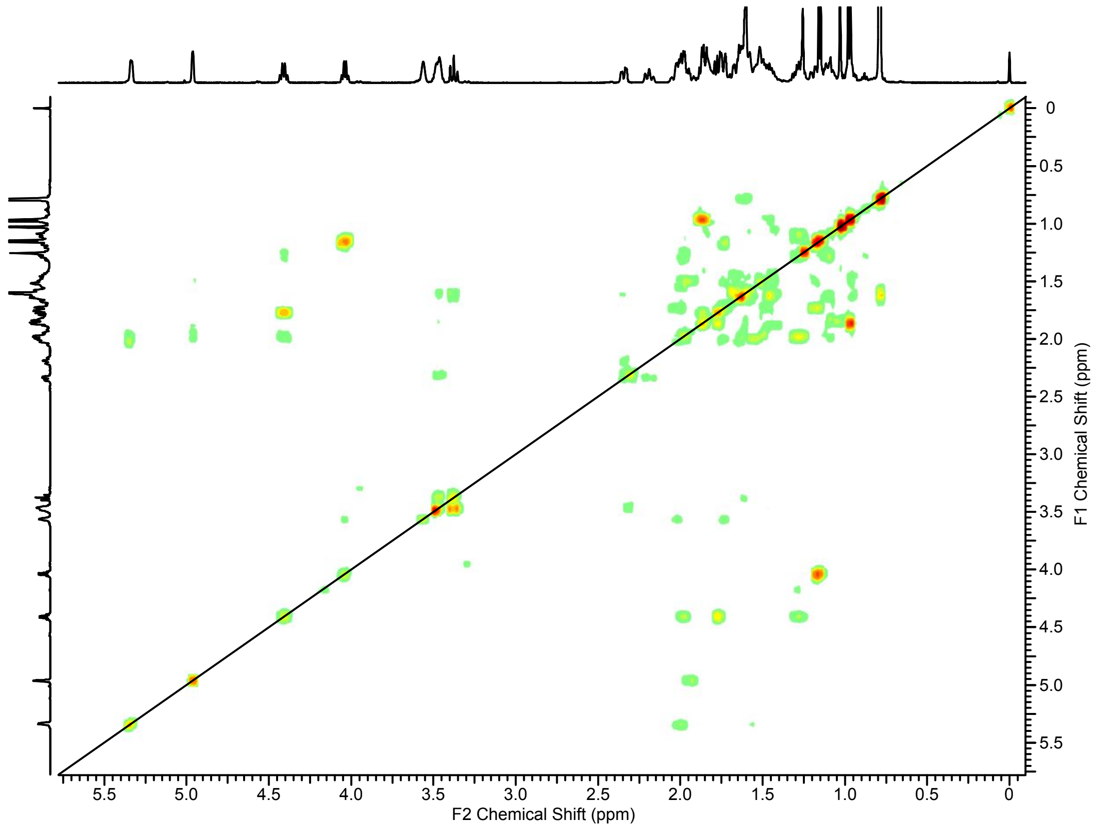

5.5 5.0 4.5 4.0 3.5 3.0 2.5 2.0 1.5 1.0 0.5 0
F2 Chemical Shift (ppm)
F1 Chemical Shift (ppm)

C

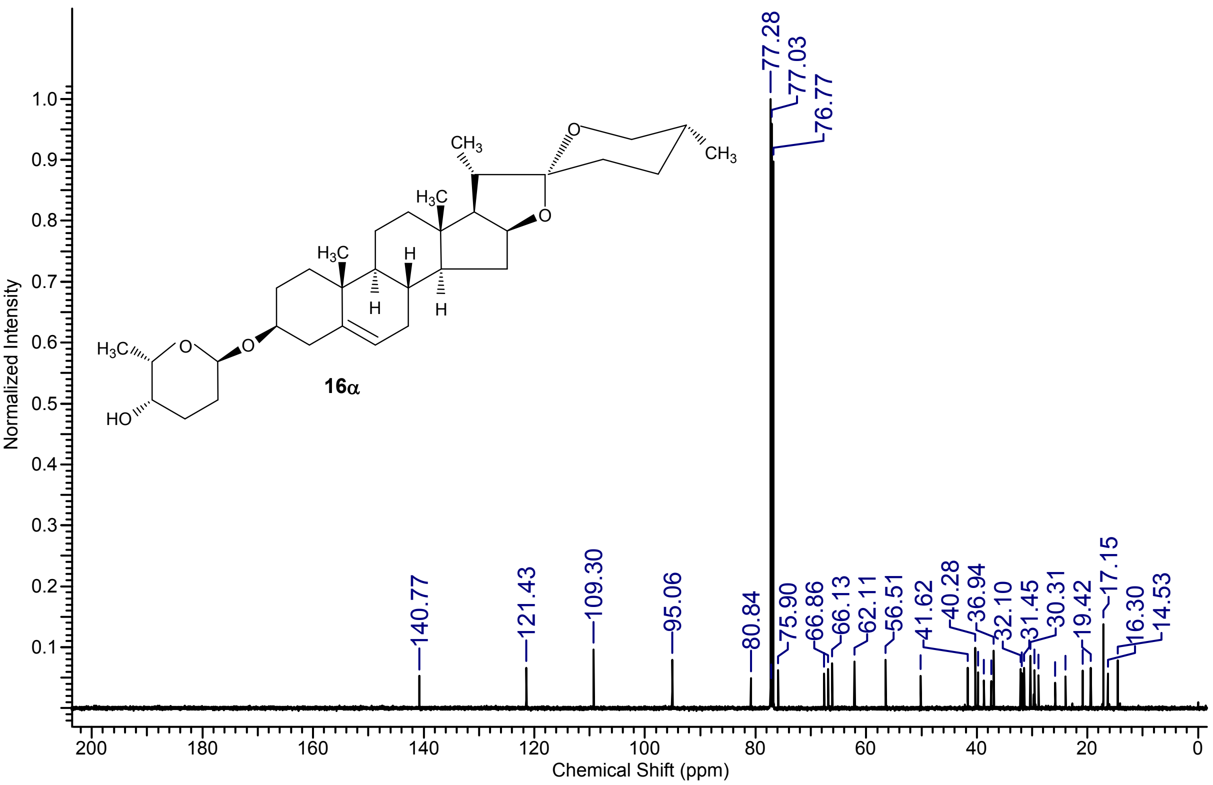

D

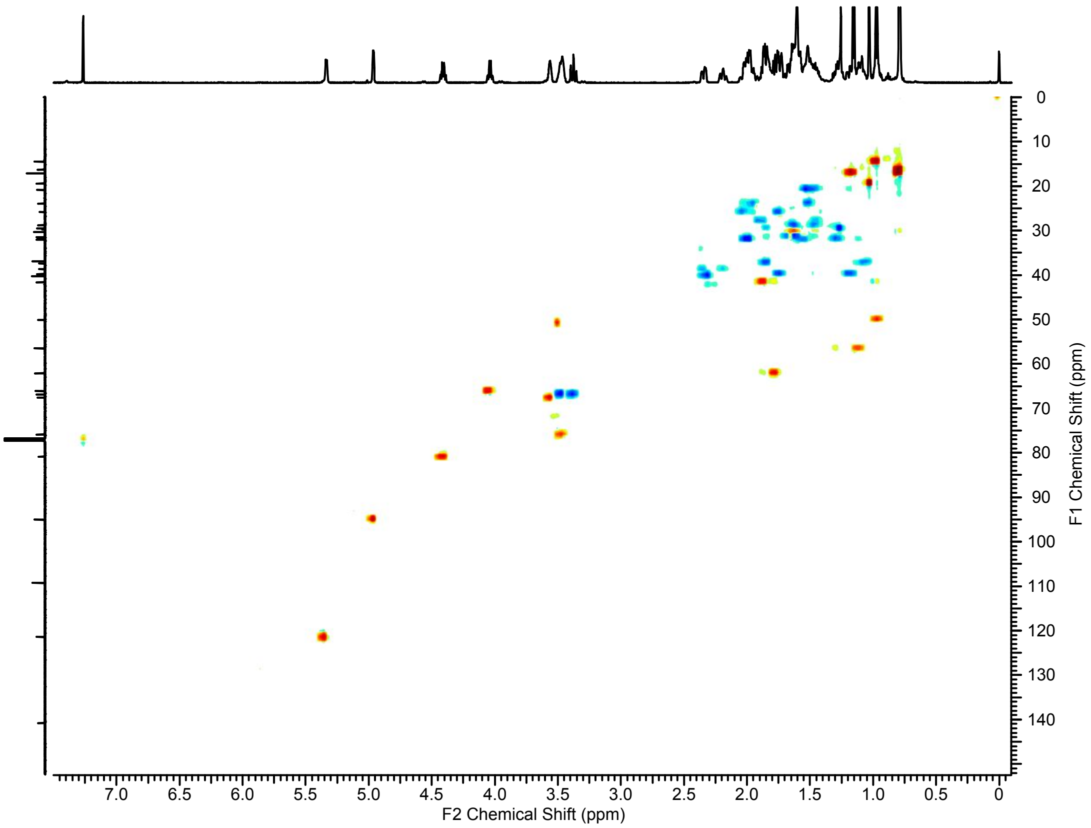

Figure S12. NMR (Chloroform-*d*) spectra of **16α**: ^{1}H NMR (500 MHz) (**A**), gCOSY (**B**), ^{13}C{^{1}H} (125 MHz) (**C**), gHSQC (**D**).

A

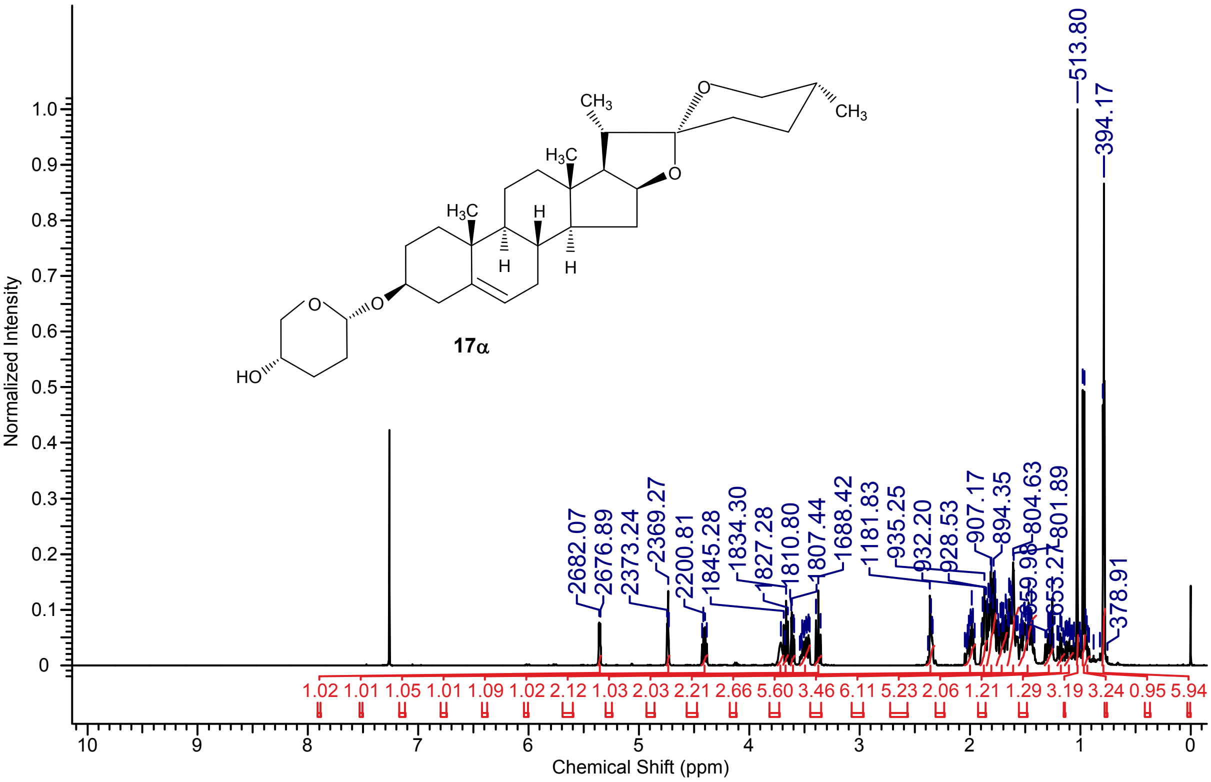

17α
CH3
H3C
H3C
H
H
H
O
O
O
O
HO
CH3
Normalized Intensity
Chemical Shift (ppm)
513.80
394.17
2682.07
2676.89
2373.24
2369.27
2200.81
1845.28
1834.30
1827.28
1810.80
1807.44
1688.42
1181.83
935.25
932.20
928.53
907.17
894.35
804.63
801.89
378.91
1.02 1.01 1.05 1.01 1.09 1.02 2.12 1.03 2.03 2.21 2.66 5.60 3.46 6.11 5.23 2.06 1.21 1.29 3.19 3.24 0.95 5.94

B

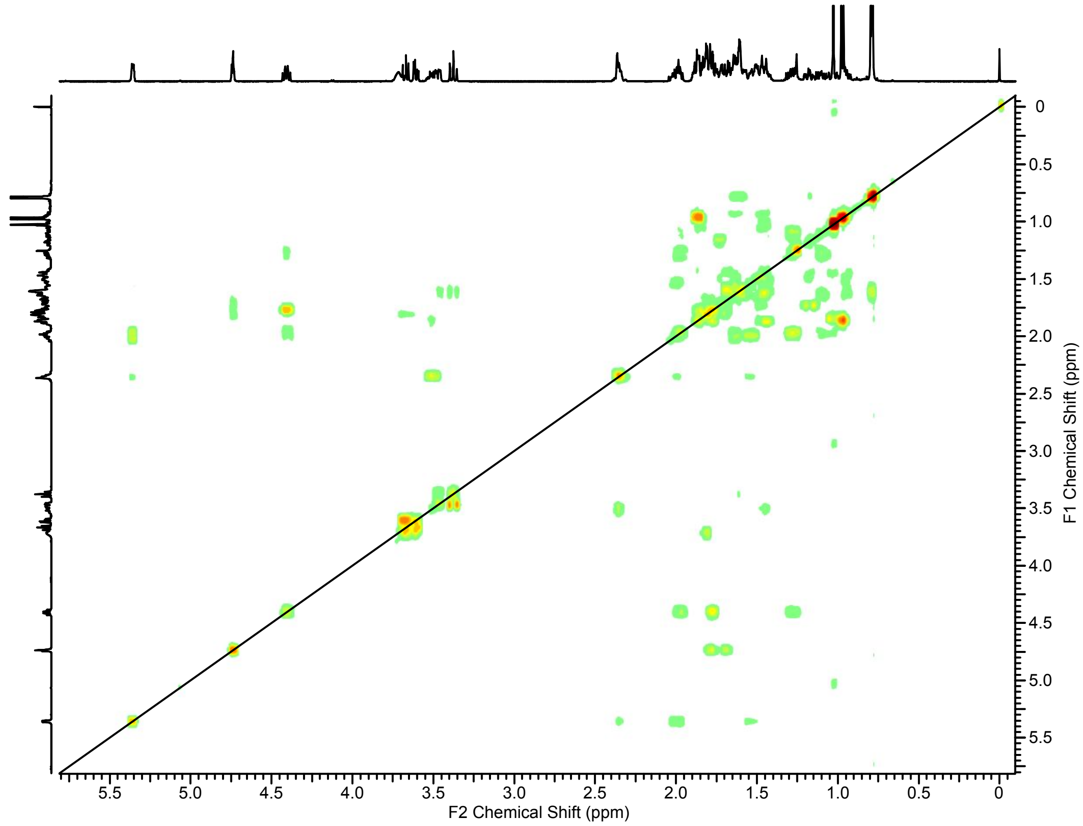

F2 Chemical Shift (ppm)
F1 Chemical Shift (ppm)

C

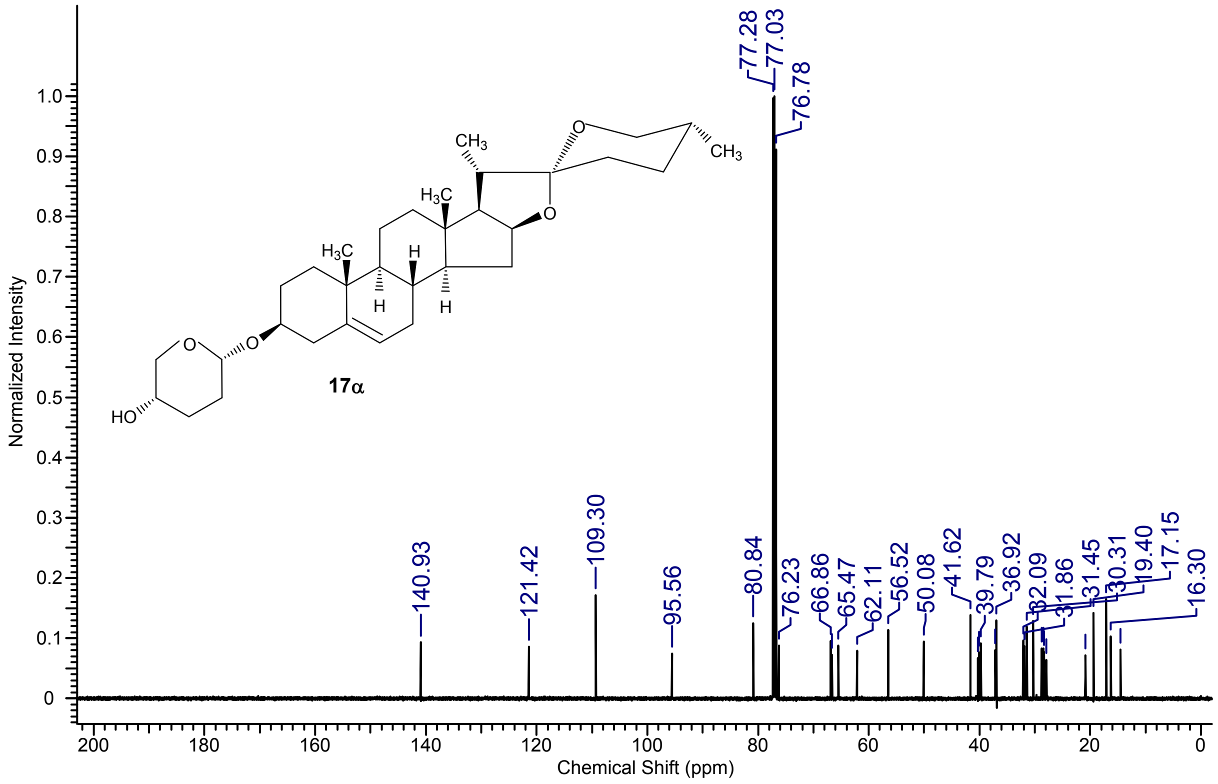

D

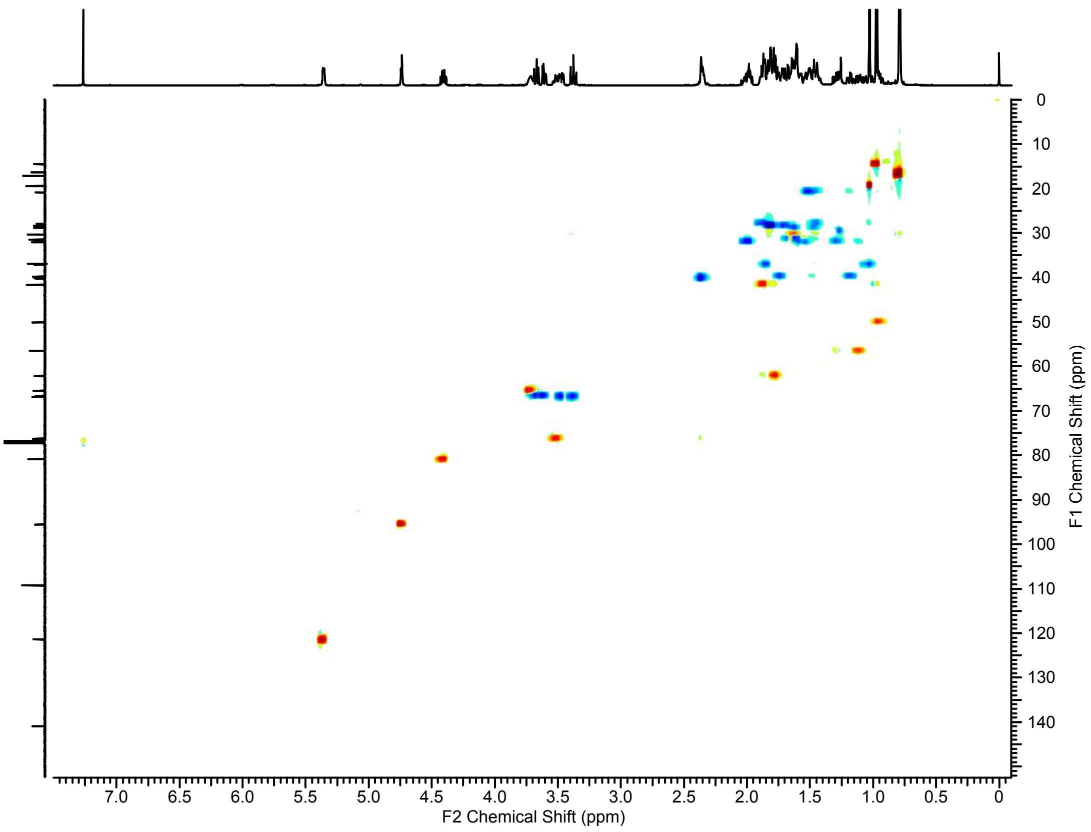

Figure S13. NMR (Chloroform-*d*) spectra of **17α**: ^{1}H NMR (500 MHz) (**A**), gCOSY (**B**), $^{13}C\{^{1}H\}$ (125 MHz) (**C**), gHSQC (**D**).

A

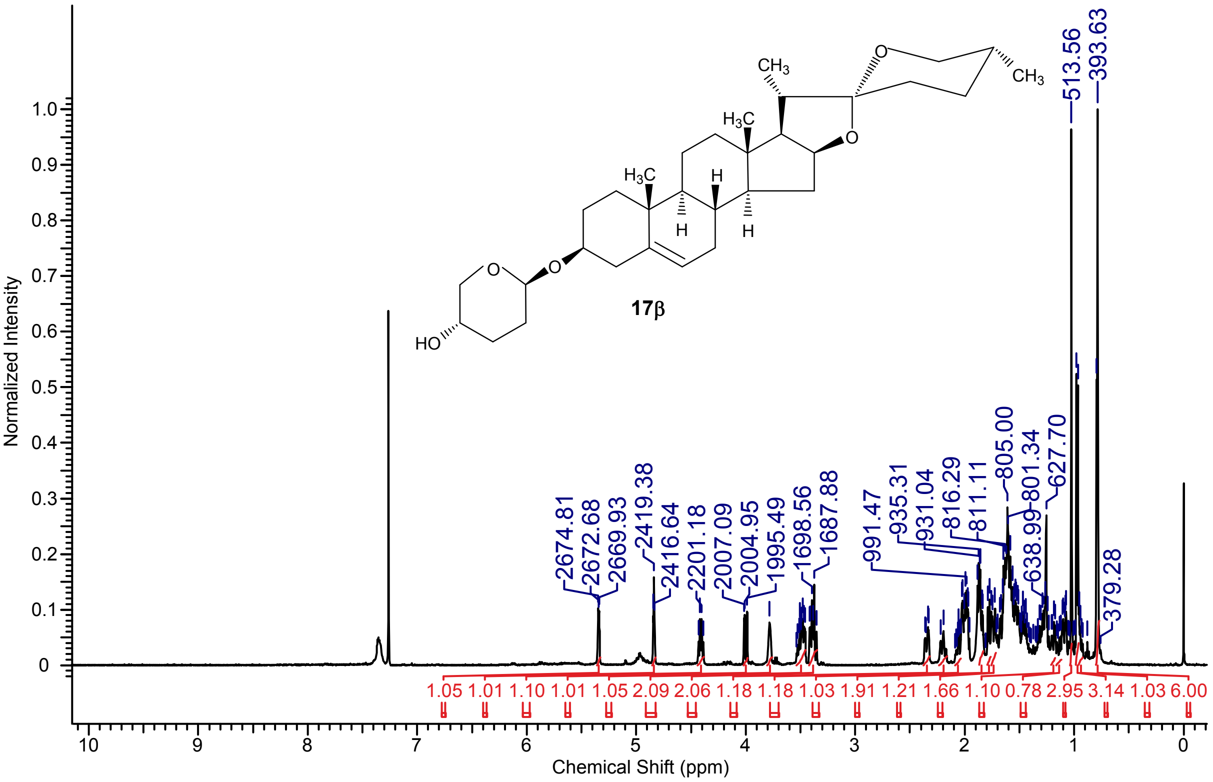
Normalized Intensity
Chemical Shift (ppm)
17β
HO
O
CH3
H3C
H
2674.81
2672.68
2669.93
2419.38
2416.64
2201.18
2007.09
2004.95
1995.49
1698.56
1687.88
991.47
935.31
931.04
816.29
811.11
805.00
801.34
638.99
627.70
513.56
393.63
379.28
1.05 1.01 1.10 1.01 1.05 2.09 2.06 1.18 1.18 1.03 1.91 1.21 1.66 1.10 0.78 2.95 3.14 1.03 6.00

B

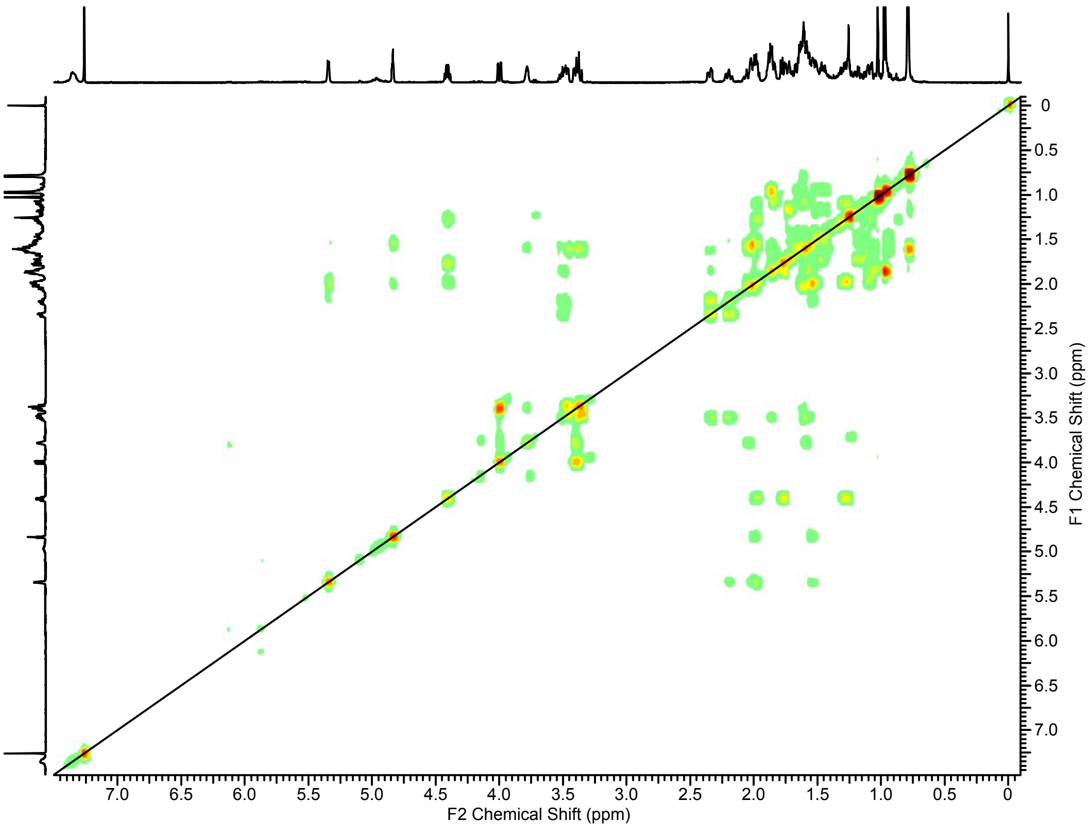
F2 Chemical Shift (ppm)
F1 Chemical Shift (ppm)

C

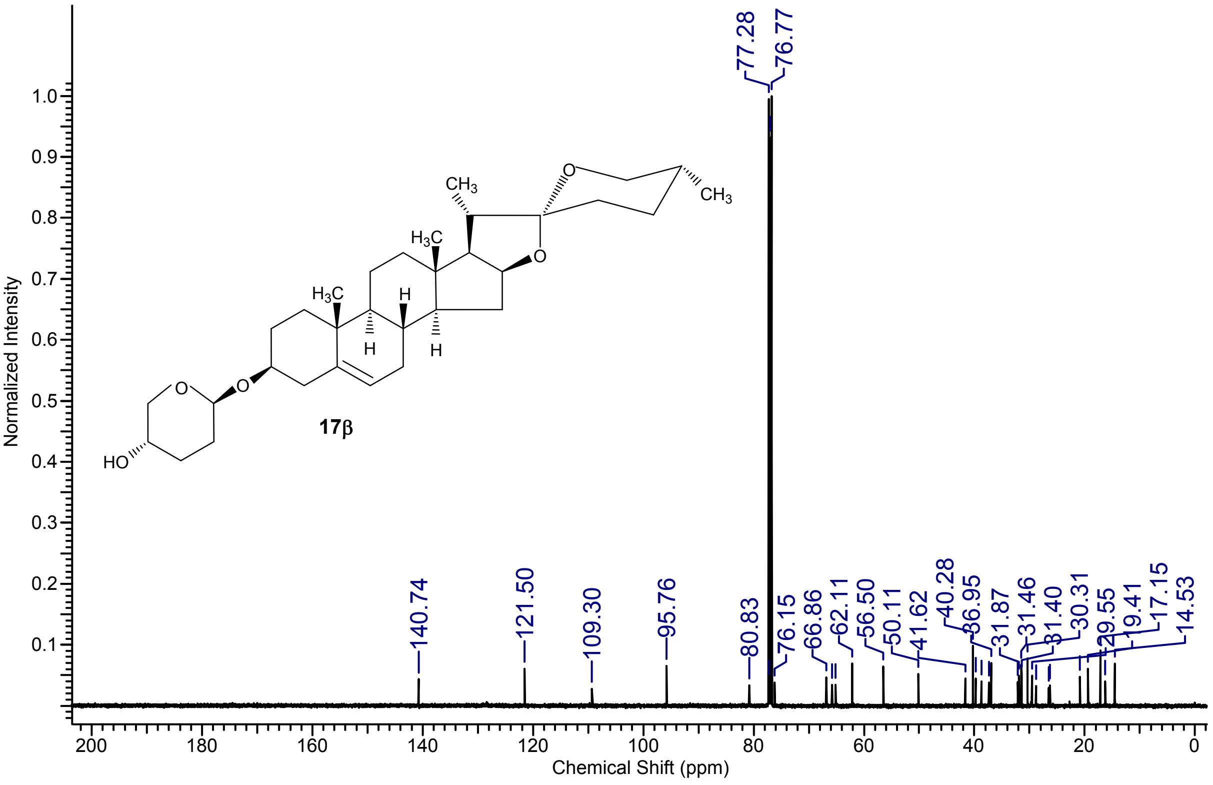

D

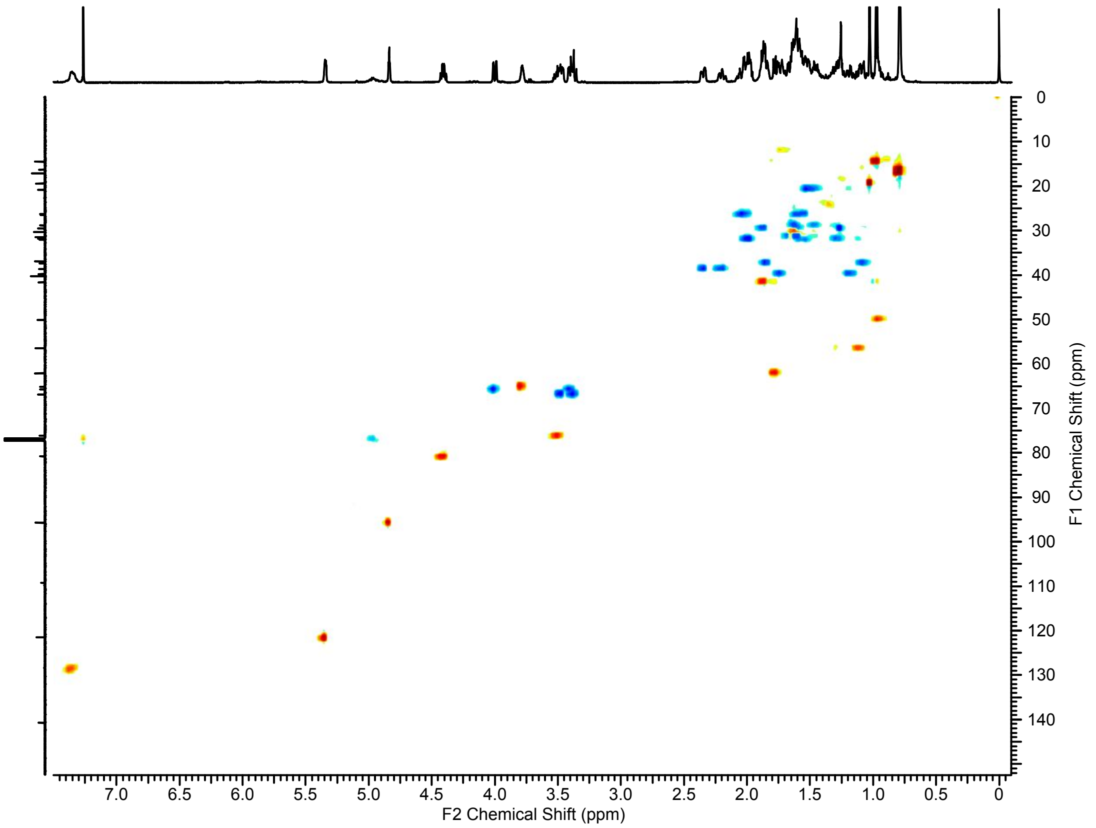

Figure S14. NMR (Chloroform-*d*) spectra of **17β**: ^{1}H NMR (500 MHz) (**A**), gCOSY (**B**), ^{13}C{^{1}H} (125 MHz) (**C**), gHSQC (**D**).

A

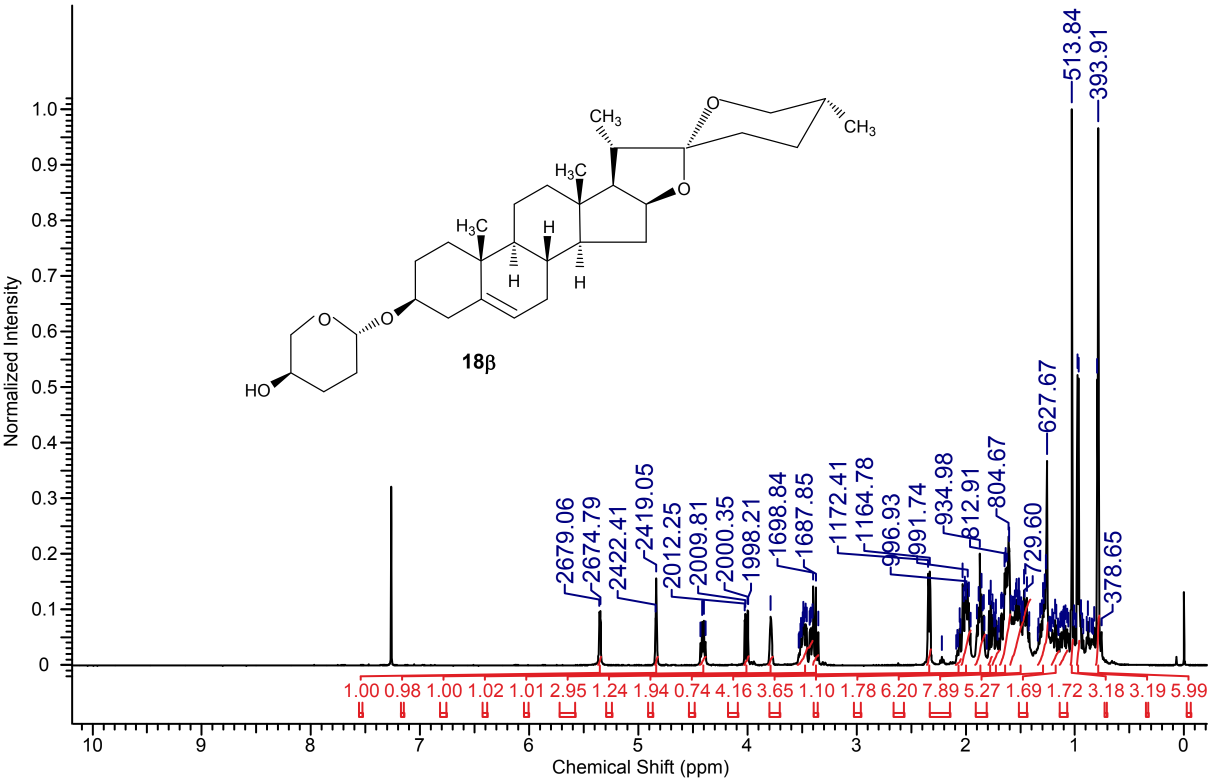

Normalized Intensity
Chemical Shift (ppm)
18β
HO
O
H3C
H
CH3
1.00 0.98 1.00 1.02 1.01 2.95 1.24 1.94 0.74 4.16 3.65 1.10 1.78 6.20 7.89 5.27 1.69 1.72 3.18 3.19 5.99
513.84
393.91
627.67
378.65

B

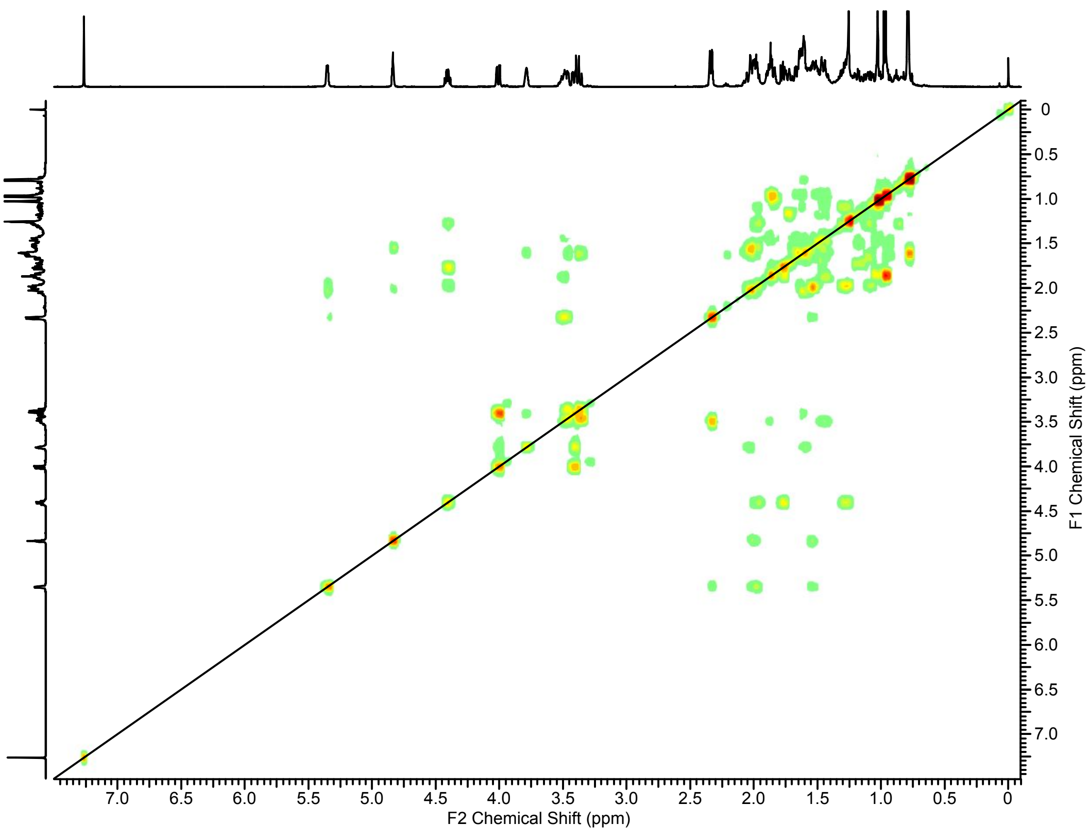

F2 Chemical Shift (ppm)
F1 Chemical Shift (ppm)

C

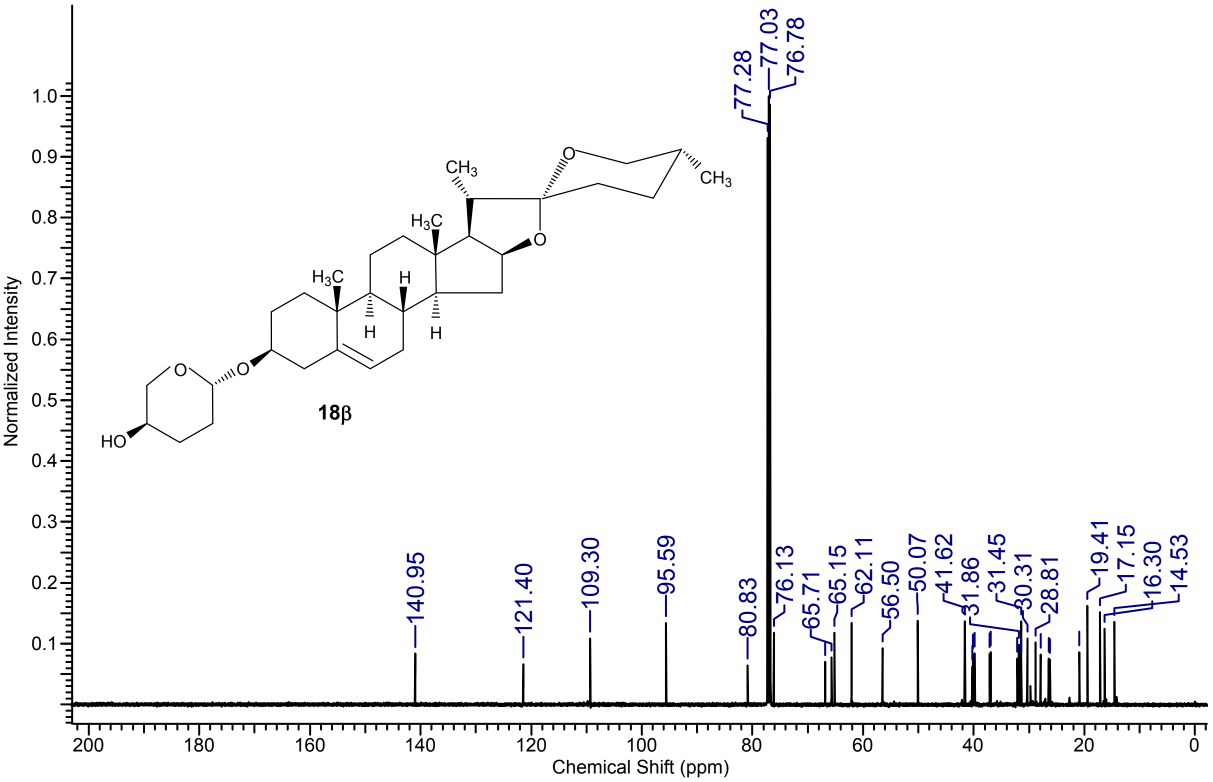

D

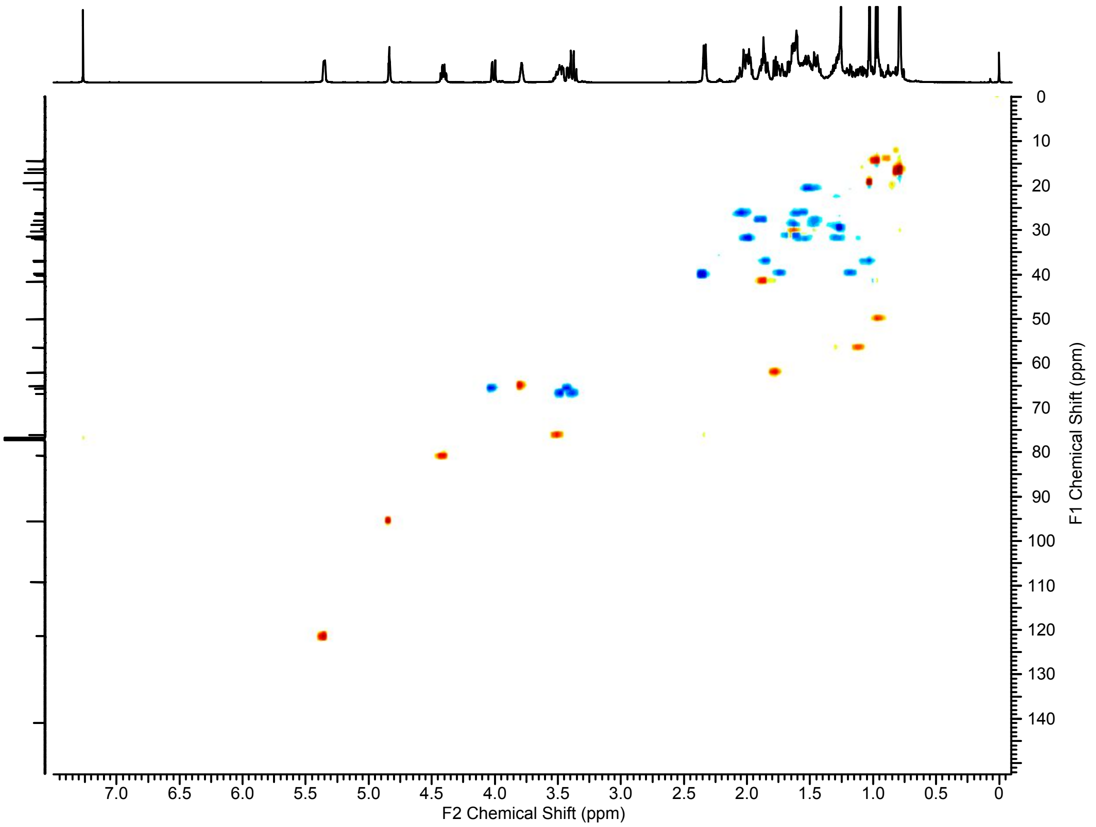

Figure S15. NMR (Chloroform-*d*) spectra of **18β**: ^{1}H NMR (500 MHz) (**A**), gCOSY (**B**), ^{13}C{^{1}H} (125 MHz) (**C**), gHSQC (**D**).

A

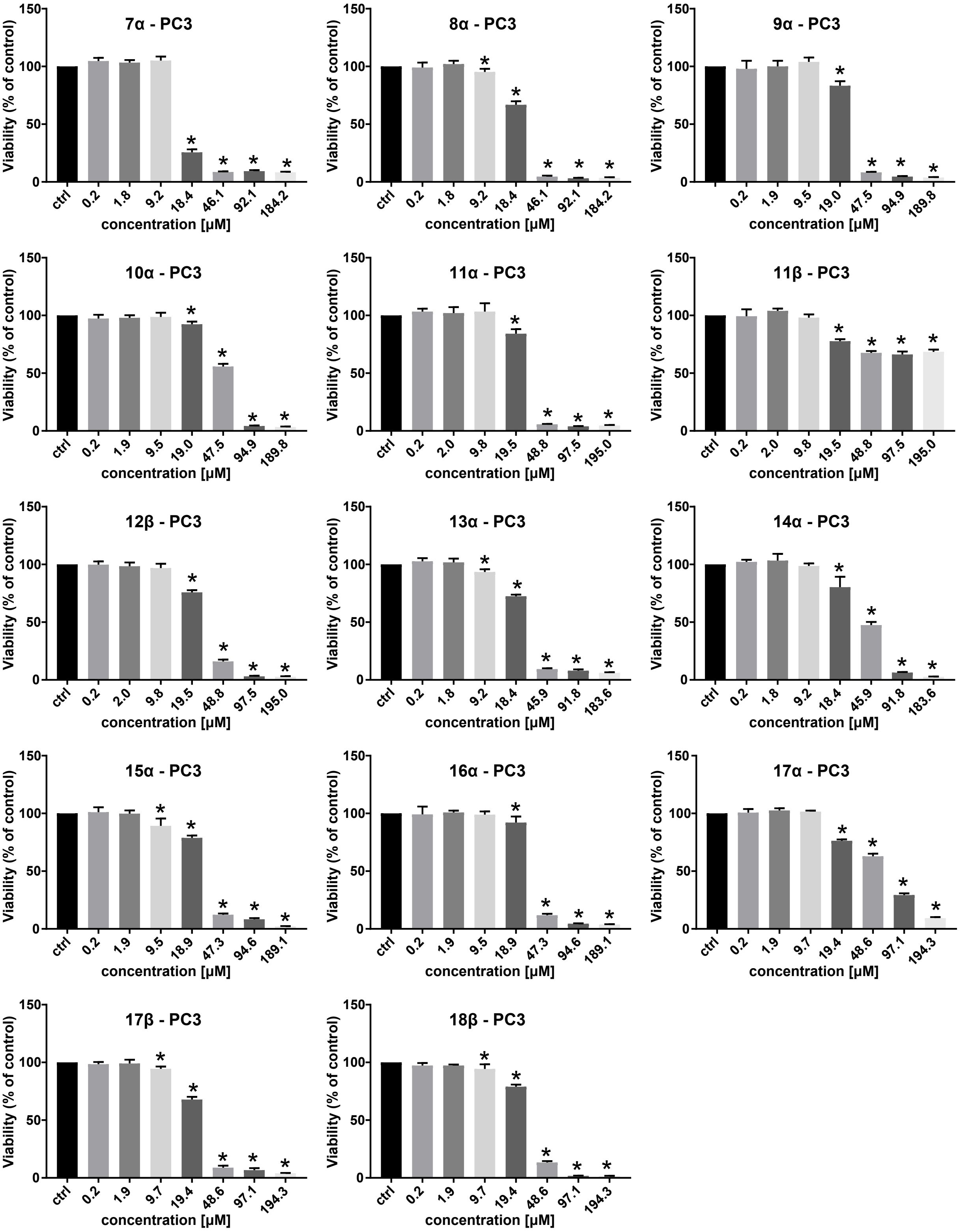

7α - PC3
8α - PC3
9α - PC3
10α - PC3
11α - PC3
11β - PC3
12β - PC3
13α - PC3
14α - PC3
15α - PC3
16α - PC3
17α - PC3
17β - PC3
18β - PC3
Viability (% of control)
concentration [μM]
150
100
50
0
ctrl
0.2 1.8 9.2 18.4 46.1 92.1 184.2
0.2 1.9 9.5 19.0 47.5 94.9 189.8
0.2 2.0 9.8 19.5 48.8 97.5 195.0
0.2 1.8 9.2 18.4 45.9 91.8 183.6
0.2 1.9 9.5 18.9 47.3 94.6 189.1
0.2 1.9 9.7 19.4 48.6 97.1 194.3
*

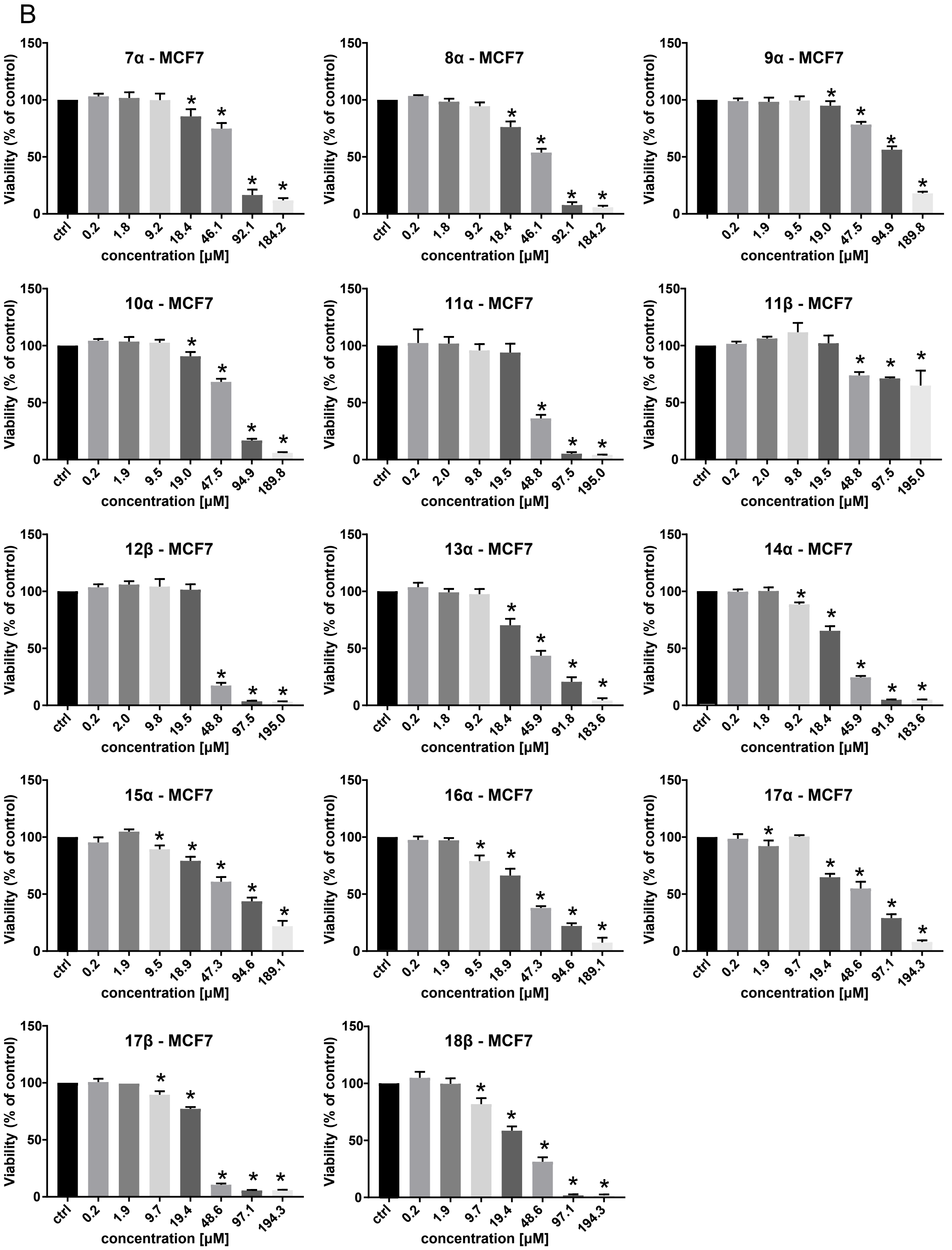

B
7α - MCF7
Viability (% of control)
150
100
50
0
ctrl
0.2
1.8
9.2
18.4
46.1
92.1
184.2
concentration [µM]
8α - MCF7
ctrl
0.2
1.8
9.2
18.4
46.1
92.1
184.2
9α - MCF7
0.2
1.9
9.5
19.0
47.5
94.9
189.8
10α - MCF7
ctrl
0.2
1.9
9.5
19.0
47.5
94.9
189.8
11α - MCF7
ctrl
0.2
2.0
9.8
19.5
48.8
97.5
195.0
11β - MCF7
ctrl
0.2
2.0
9.8
19.5
48.8
97.5
195.0
12β - MCF7
ctrl
0.2
2.0
9.8
19.5
48.8
97.5
195.0
13α - MCF7
ctrl
0.2
1.8
9.2
18.4
45.9
91.8
183.6
14α - MCF7
ctrl
0.2
1.8
9.2
18.4
45.9
91.8
183.6
15α - MCF7
ctrl
0.2
1.9
9.5
18.9
47.3
94.6
189.1
16α - MCF7
ctrl
0.2
1.9
9.5
18.9
47.3
94.6
189.1
17α - MCF7
ctrl
0.2
1.9
9.7
19.4
48.6
97.1
194.3
17β - MCF7
ctrl
0.2
1.9
9.7
19.4
48.6
97.1
194.3
18β - MCF7
ctrl
0.2
1.9
9.7
19.4
48.6
97.1
194.3

C

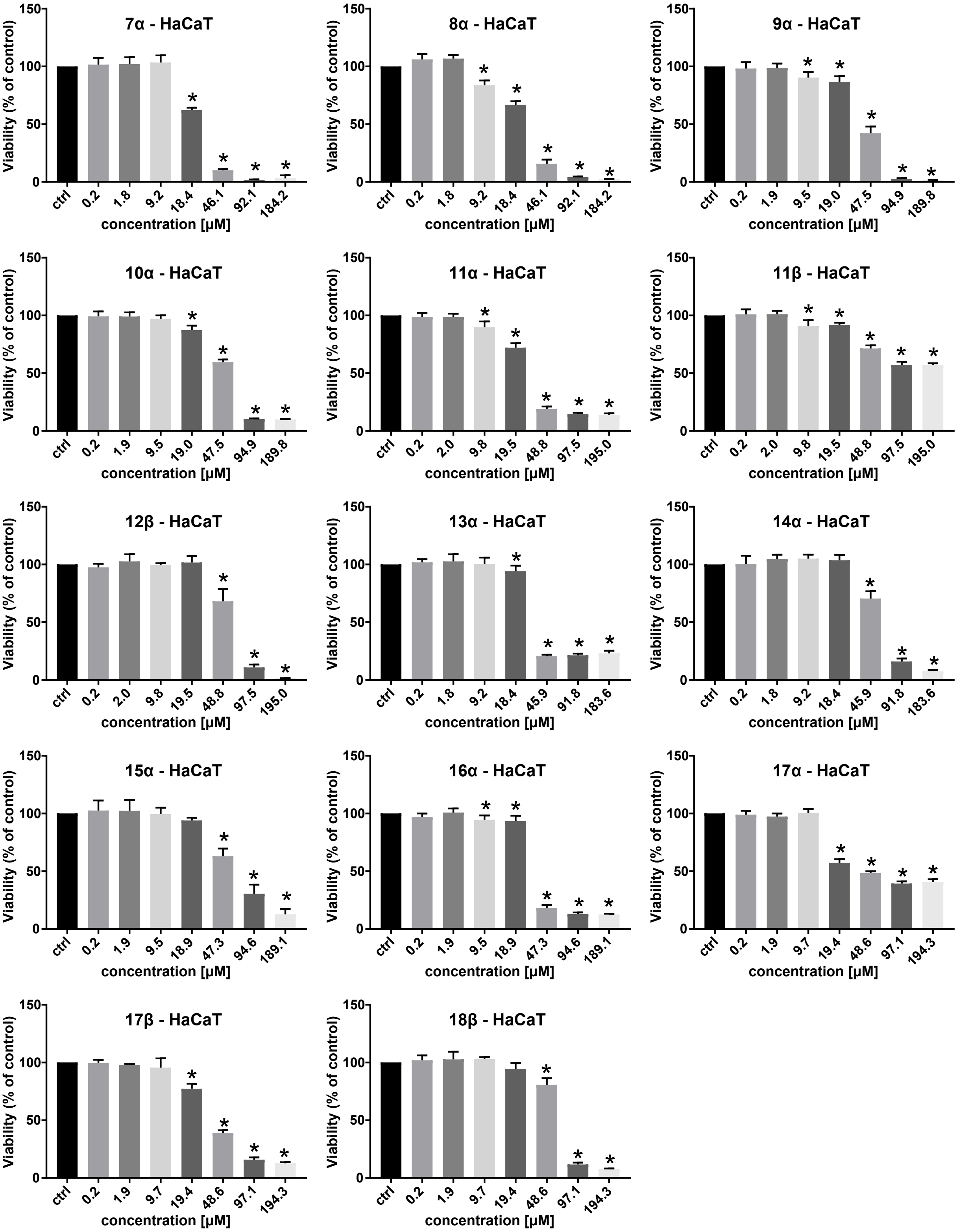

Figure S16. The viability of PC3 (A), MCF7 (B) and HaCaT (C) cells after 48 h treatment with **7α-18β**. The results are shown as the mean ± standard deviation (SD) of three independent experiments performed in triplicate.* A statistically significant difference is present between treated cultures compared with control (culture treated with vehicle), $p < 0.05$.

Table S1. IC_{50} values (μM) of **7α-18β** towards cancer (PC3 and MCF7) and normal (HaCaT) cells together with selectivity indexes (SI)*.

**Comp.**	**IC_{50} [μM]**			**SI**	
	**PC3**	**MCF7**	**HaCaT**	**PC3**	**MCF7**
**7α**	16.84	56.14	20.18	1.20	0.36
**8α**	21.35	44.94	22.24	1.04	0.49
**9α**	25.38	182.67	43.44	1.71	0.24
**10α**	49.74	57.58	50.86	1.02	0.88
**11α**	24.44	40.59	24.48	1.00	0.60
**11β**		inactive		–	–
**12β**	27.85	35.61	57.87	2.08	1.62
**13α**	22.51	38.60	21.62	0.96	0.56
**14α**	41.08	25.02	53.20	1.29	2.13
**15α**	26.53	87.09	56.85	2.14	0.65
**16α**	29.88	36.59	30.60	1.02	0.84
**17α**	73.20	68.25	48.34	0.66	0.71
**17β**	23.20	26.27	33.88	1.46	1.29
**18β**	28.87	26.85	59.86	2.07	2.23

* The SI (selectivity index) represents the ratio of IC_{50} for normal cell line to IC_{50} for cancer cell line after 48 h of treatment.

Table S2. Minimum inhibitory concentrations [μg/ml] of **7α-18β** determined on G(+) bacteria.

	S. aureus **ATCC 6538**	***S. aureus*** **ATCC 43300**	***S. aureus*** **ATCC 25923**	***S. aureus*** **ATCC 12598**	***S. aureus*** **ATCC 33591**	***S. epidermidis*** **ATCC 14990**	***E. faecium*** **ATCC 29212**
**7α**	256	> 256	> 256	> 256	> 256	256	256
**8α**	> 256	> 256	> 256	> 256	> 256	> 256	> 256
**9α**	> 256	> 256	> 256	> 256	> 256	> 256	> 256
**10α**	> 256	> 256	> 256	> 256	> 256	> 256	> 256
**11α**	256	> 256	> 256	> 256	> 256	256	> 256
**11β**	> 256	> 256	> 256	> 256	> 256	> 256	> 256
**12β**	256	> 256	> 256	> 256	> 256	> 256	> 256
**13α**	> 256	> 256	> 256	> 256	> 256	> 256	> 256
**14α**	64	128	64	256	256	> 256	>256
**15α**	256	> 256	256	> 256	> 256	> 256	> 256
**16α**	> 256	> 256	> 256	> 256	> 256	> 256	> 256
**17α**	128	128	256	256	256	> 256	> 256
**17β**	256	> 256	> 256	> 256	> 256	> 256	> 256
**18β**	> 256	> 256	> 256	> 256	> 256	> 256	> 256

Table S3. Total energies (a.u.), Gibbs free energies (a.u.), the lowest frequencies and Cartesian coordinates of optimized structures **1'-5'** and **1''-5''**.

1' E = -764.650756844
G = -764.467621
Lowest frequency: 31.5285

C	0.421829	0.197274	-0.891149
C	0.318031	0.382042	0.433516
C	1.494004	0.221205	1.348198
C	2.684475	-0.464605	0.678143
O	2.773138	-0.169656	-0.761142
C	1.727214	-0.014874	-1.445117
O	1.797576	1.524941	1.815491
C	2.578713	1.549927	2.952855
O	3.002501	0.533387	3.417946
C	2.698927	-1.974610	0.866130
O	1.411695	-2.593842	0.804169
C	0.939917	-2.962253	-0.411185
O	1.416408	-2.534882	-1.432127
C	2.781246	2.943883	3.444849
C	-0.207010	-3.910931	-0.297431
H	1.907245	0.044904	-2.516413
H	-0.406153	0.311928	-1.577185
H	-0.616461	0.695139	0.887834
H	1.200486	-0.413811	2.191462
H	3.621370	-0.067224	1.062830
H	3.155748	3.572468	2.636334
H	1.821396	3.352148	3.766670
H	3.479449	2.931295	4.277138
H	3.054280	-2.159133	1.880571
H	3.377613	-2.434884	0.145425
H	-0.672180	-4.039990	-1.270778
H	0.167151	-4.871118	0.064525
H	-0.926508	-3.544087	0.435534

1'' E = -764.657371234
G = -764.470872
Lowest frequency: 44.4363

C	0.039627	-0.075375	0.081197
C	0.090574	-0.104579	1.417673
C	1.386422	-0.180405	2.125802
C	2.594889	-0.153820	1.182051
C	2.325575	-0.632844	-0.232509
O	1.093016	-0.146711	-0.748603
O	2.936834	1.278474	1.165889
C	2.371054	1.855102	2.161461
C	2.597969	3.279131	2.454430
C	2.394326	-2.153828	-0.294227
O	1.652490	-2.742072	0.772853
C	0.391027	-3.197380	0.473008
C	-0.232445	-3.855753	1.663149
O	1.591218	1.131790	2.853926
O	-0.102540	-3.036111	-0.601215
H	-0.890883	0.014116	-0.466174
H	-0.823041	-0.055367	1.993043
H	1.455300	-0.935525	2.904640
H	3.467348	-0.660628	1.596168
H	3.096067	-0.223859	-0.889773
H	3.601809	3.569055	2.150091
H	1.871386	3.850718	1.866130
H	2.420235	3.470355	3.510966
H	3.428872	-2.477183	-0.166458
H	2.015109	-2.488614	-1.259665
H	-1.152090	-4.348355	1.358799
H	0.459291	-4.569350	2.110111
H	-0.461606	-3.090769	2.409907

2' E = -764.6473594
G = -764.464967
Lowest frequency: 8.4945

C	-0.031661	-0.353766	-0.084286
C	0.300307	-0.166277	1.370968
C	1.804637	-0.262196	1.616801
O	2.485912	-1.231625	0.743852
C	2.052482	-1.449091	-0.414140

2'' E = -764.6597158
G = -764.472254
Lowest frequency: 34.6490

C	0.024334	-0.257216	-0.027029
C	0.172110	0.183104	1.380141
C	1.609456	0.067796	1.903173
C	2.654977	0.068903	0.808716
O	2.321792	-0.847654	-0.230483

C	0.850993	-0.883794	-0.948557	C	1.058649	-0.782347	-0.692712
O	-0.545563	-0.957569	2.197377	O	1.582987	-1.244835	2.579297
C	-0.424493	-2.317577	2.129167	C	0.365431	-1.591437	2.768668
O	0.420853	-2.826958	1.447710	C	0.024567	-2.798877	3.539379
C	2.191861	-0.599147	3.046447	C	4.072430	-0.274475	1.290277
O	3.525819	-0.222972	3.327267	O	4.977571	0.811682	1.143085
C	3.727604	1.119003	3.469611	C	4.759343	1.869104	1.952904
C	5.120337	1.423330	3.914762	O	3.787506	1.922219	2.664572
O	2.846902	1.904853	3.249714	O	-0.517693	-0.834580	2.258426
C	-1.437798	-3.000072	2.985858	C	5.836696	2.897754	1.838360
H	2.655297	-2.153909	-0.984706	H	0.967394	-1.204153	-1.686817
H	0.629907	-1.047470	-1.994588	H	-0.951060	-0.215398	-0.490610
H	-1.032746	-0.078207	-0.402978	H	-0.317139	1.121847	1.627781
H	0.035720	0.857203	1.651980	H	1.872772	0.798299	2.666266
H	2.239094	0.695385	1.320619	H	2.644504	1.080702	0.383149
H	-1.261880	-4.071977	2.963912	H	-1.051449	-2.949853	3.542520
H	-2.437058	-2.773630	2.609236	H	0.403315	-2.681010	4.557497
H	-1.377253	-2.619601	4.006273	H	0.537273	-3.652295	3.089272
H	2.136771	-1.676566	3.201354	H	4.463267	-1.066773	0.655769
H	1.498512	-0.087523	3.717737	H	4.067637	-0.606010	2.330778
H	5.294304	2.493758	3.849101	H	5.562616	3.775221	2.417184
H	5.239358	1.092540	4.948506	H	6.770240	2.476591	2.216333
H	5.838531	0.872831	3.307141	H	5.993039	3.157123	0.790907

3' E = -536.787192903
G = -536.641972
Lowest frequency: 19.6513

C	0.050714	-0.187162	-0.147781
C	0.002199	0.050105	1.336041
C	1.380131	-0.036209	2.003097
O	2.310692	-0.941829	1.299238
C	2.196804	-1.179906	0.072709
C	1.132842	-0.700850	-0.754556
O	-1.017331	-0.734184	1.941165
C	-0.833827	-2.089968	1.970559
O	0.167722	-2.582524	1.532801
H	1.298889	-0.498001	2.986204
C	-1.999038	-2.786182	2.589785
H	2.977461	-1.829410	-0.320496
H	1.182474	-0.894929	-1.817338
H	-0.847950	0.051021	-0.709347
H	-0.349835	1.074335	1.489549
C	2.081918	1.307857	2.074081
H	-2.875193	-2.651813	1.952269
H	-1.771320	-3.843428	2.693195

3'' E = -536.796622300
G = -536.646131
Lowest frequency: 89.7409

O	0.069453	-0.267749	0.073494
C	0.120665	-0.055354	1.568837
C	1.638171	0.038666	1.775819
O	2.108359	0.370075	0.419095
C	1.179434	0.105344	-0.421395
C	2.074594	1.105037	2.759085
O	1.410612	2.344677	2.515165
C	0.092538	2.294109	2.268547
C	-0.584591	1.209267	1.873580
C	1.838020	0.644850	4.192680
C	1.383857	0.254229	-1.870831
H	3.132021	1.315150	2.594288
H	-0.385377	3.253748	2.426860
H	-1.654103	1.262076	1.728319
H	-0.332685	-0.954522	1.979641
H	2.100551	-0.918895	2.017873
H	2.427642	0.077378	-2.122705
H	0.714913	-0.416860	-2.406556

	H	-2.224905	-2.342110	3.559649		H	1.126195	1.286990	-2.130723
	H	3.082538	1.192477	2.489004		H	2.151059	1.437377	4.871030
	H	2.152335	1.771949	1.087147		H	0.783374	0.428927	4.377271
	H	1.510473	1.967205	2.729383		H	2.426687	-0.247955	4.410281
**4'**	E = -536,788460778				**4''**	E = -536,797889200			
	G = -536,641587					G = -536,648453			
	Lowest frequency: 52.0262					Lowest frequency: 52.3046			
	C	0.091199	0.378893	-0.047267		O	-0.011558	0.020904	0.003939
	C	-0.011326	0.293807	1.451347		C	-0.002433	0.002282	1.516479
	C	1.354291	0.062930	2.106000		C	1.510526	-0.003396	1.783784
	O	2.457750	0.731915	1.385742		O	2.056322	-0.409911	0.478922
	C	2.399991	0.896076	0.142294		C	1.142456	-0.303364	-0.413171
	C	1.268538	0.565765	-0.668333		C	1.941064	-0.973026	2.866177
	O	-0.804979	1.347785	1.976281		O	1.343804	-2.254220	2.686578
	C	-0.329177	2.624713	1.861298		C	0.022547	-2.266498	2.445515
	C	-1.289799	3.617134	2.425598		C	-0.680486	-1.234513	1.963810
	H	1.593570	-0.996432	1.972739		C	3.440478	-1.163030	2.942815
	O	0.742656	2.840481	1.369249		C	1.436596	-0.540631	-1.835847
	H	3.287378	1.363944	-0.281631		H	1.549624	-0.554809	3.804323
	H	1.365524	0.648167	-1.742467		H	-0.429754	-3.220450	2.690777
	H	-0.836359	0.319989	-0.609378		H	-1.749732	-1.322174	1.833724
	H	-0.597826	-0.597974	1.690360		H	-0.501016	0.928342	1.791966
	C	1.464563	0.473436	3.553021		H	1.917633	0.989444	1.978770
	H	-2.247253	3.536379	1.908799		H	2.122403	0.237918	-2.181028
	H	-1.465129	3.392449	3.479228		H	1.948145	-1.500717	-1.930670
	H	-0.879375	4.616741	2.313908		H	0.518774	-0.524781	-2.417103
	H	2.434506	0.178293	3.951163		H	3.672655	-1.849927	3.755400
	H	1.349496	1.551985	3.658559		H	3.821049	-1.581722	2.010809
	H	0.682615	-0.026730	4.126465		H	3.935854	-0.210792	3.139340
**5'**	E = -497.473972252				**5''**	E = -497.485481639			
	G = -497.353783					G = -497.362243			
	Lowest frequency: 61.3559					Lowest frequency: 57.6294			
	C	0.037704	-0.041663	-0.029874		O	0.001545	0.044832	0.008695
	C	0.071040	0.118610	1.486943		C	-0.000904	0.013429	1.518645
	O	1.372918	-0.113789	2.101912		C	1.510381	0.014889	1.797526
	C	2.207599	-0.879827	1.557593		O	2.069023	-0.374766	0.493487
	C	2.021892	-1.514942	0.290068		C	1.159633	-0.276167	-0.403461
	C	1.039818	-1.057342	-0.505746		C	1.927592	-0.971265	2.860612
	O	0.130713	1.158116	-0.783445		O	1.352328	-2.250840	2.682773
	C	1.251856	1.925955	-0.614669		C	0.031563	-2.265900	2.431289
	C	1.225383	3.133747	-1.489000		C	-0.672181	-1.233352	1.951725
	O	2.102016	1.602538	0.166235		C	1.443963	-0.550051	-1.820697
	H	3.127297	-0.996316	2.128416		H	-0.415984	-3.224728	2.665012

H	2.763780	-2.231013	-0.036599	H	-1.739765	-1.327160	1.813471
H	0.958764	-1.381110	-1.539217	H	-0.507842	0.933107	1.800064
H	-0.959194	-0.415307	-0.277760	H	1.911992	1.006065	2.009244
H	-0.213839	1.117806	1.808383	H	3.008048	-1.104601	2.862261
H	-0.580768	-0.620630	1.955957	H	1.610659	-0.564724	3.828375
H	0.335737	3.725327	-1.267309	H	1.312083	-1.626747	-1.975755
H	2.123664	3.719891	-1.316659	H	0.733528	-0.016225	-2.449005
H	1.164741	2.826073	-2.534098	H	2.473845	-0.285928	-2.053411
